# Transcriptomic Analysis of Grape (*Vitis vinifera* L.) Leaves after Exposure to Ultraviolet C Irradiation

**DOI:** 10.1371/journal.pone.0113772

**Published:** 2014-12-02

**Authors:** Huifen Xi, Ling Ma, Guotian Liu, Nian Wang, Junfang Wang, Lina Wang, Zhanwu Dai, Shaohua Li, Lijun Wang

**Affiliations:** 1 Key Laboratory of Plant Resources and Beijing Key Laboratory of Grape Science and Enology, Institute of Botany, the Chinese Academy of Sciences, Beijing, P.R. China; 2 University of Chinese Academy of Sciences, Beijing, P.R. China; 3 Key Laboratory of Plant Germplasm Enhancement and Specialty Agriculture, Wuhan Botanical Garden, Chinese Academy of Sciences, Wuhan, P.R.China; 4 INRA, ISVV, UMR 1287 EGFV, Villenave d'Ornon, France; Tulane University Health Sciences Center, United States of America

## Abstract

**Background:**

Only a small amount of solar ultraviolet C (UV-C) radiation reaches the Earth's surface. This is because of the filtering effects of the stratospheric ozone layer. Artificial UV-C irradiation is used on leaves and fruits to stimulate different biological processes in plants. Grapes are a major fruit crop and are grown in many parts of the world. Research has shown that UV-C irradiation induces the biosynthesis of phenols in grape leaves. However, few studies have analyzed the overall changes in gene expression in grape leaves exposed to UV-C.

**Methodology/Principal Findings:**

In the present study, transcriptional responses were investigated in grape (*Vitis vinifera* L.) leaves before and after exposure to UV-C irradiation (6 W·m^−2^ for 10 min) using an Affymetrix *Vitis vinifera* (Grape) Genome Array (15,700 transcripts). A total of 5274 differentially expressed probe sets were defined, including 3564 (67.58%) probe sets that appeared at both 6 and 12 h after exposure to UV-C irradiation but not before exposure. A total of 468 (8.87%) probe sets and 1242 (23.55%) probe sets were specifically expressed at these times. The probe sets were associated with a large number of important traits and biological pathways, including cell rescue (i.e., antioxidant enzymes), protein fate (i.e., HSPs), primary and secondary metabolism, and transcription factors. Interestingly, some of the genes involved in secondary metabolism, such as stilbene synthase, responded intensely to irradiation. Some of the MYB and WRKY family transcription factors, such as VvMYBPA1, VvMYB14, VvMYB4, WRKY57-like, and WRKY 65, were also strongly up-regulated (about 100 to 200 fold).

**Conclusions:**

UV-C irridiation has an important role in some biology processes, especially cell rescue, protein fate, secondary metabolism, and regulation of transcription.These results opened up ways of exploring the molecular mechanisms underlying the effects of UV-C irradiation on grape leaves and have great implications for further studies.

## Introduction

In nature, solar radiation comprises electromagnetic radiation of different wavelengths and broadly classified as ultraviolet radiation (UV≈200–400 nm), photosynthetically active radiation (PAR≈400–700 nm), and far red radiation (FR≈700–780 nm). Approximately 7–9% of all solar radiation that reaches the Earth's surface is in the UV range. UV radiation is broadly classified based on wavelength as UV-A radiation (320–400 nm), which cannot be absorbed by the stratospheric ozone layer and is fully transmitted to the Earth's surface. UV-B radiation (280–320 nm), which is filtered through the ozone layer and, therefore makes up only a small amount of the radiation that reaches the Earth's surface; and UV-C radiation (200–280 nm), which is the most hazardous range of UV light, but it is physiologically insignificant because these wavelengths are almost completely absorbed by the atmosphere [1]–[3]. It is therefore important to study the effects of UV radiation on plants in detail. The current knowledge regarding the ecophysiological impact of UV radiation on plants has come largely from field experiments involving natural and artifical UV radiation [4]–[5]. UV-B and UV-C may penetrate plant tissues, damage proteins and membranes, and block replication and transcription of DNA, but UV-A has not been found to have any deleterious effect [5]–[7].

Although more studies have focused on UV-B and UV-A than on UV-C, some recent studies have reported that artificial UV-C has many regulatory effects on plant morphology, physiology, and biochemistry [8]–[13]. UV-C irradiation has been shown to increase the accumulation of flavonoids, triterpene, and resveratrol compounds in lettuce, *Quillaja brasiliensis*, and peanut leaves [14]–[15]. It also led to a decrease in pea fresh weight and in the concentration of leaf pigments and free proline in pea plants. This was accompanied by an increase in malondialdehyde [16]. UV-C radiation decreased soluble carbohydrates, reducing sugar, chlorophyll, and proline concentrations and increasing the concentrations of UV-absorbing pigments, soluble proteins, and glucosinolate in canola leaves (*Brassica napus* L.) [17]. It increased jasmonate and polyamine concentrations in leaves of apple seedlings and scoparone content in citrus leaves [10], [18]. The production of these compounds is associated with other inducible defenses, such as cell wall modification, defense enzymes, and antioxidant activity. Pre-storage treatment of table grapes, tomatoes, mangoes, and citrus fruit with low doses of UV-C can reduce postharvest decay [19]–[21]. UV-C was found to promote the expression of an array of genes [8]–[11], [22].

Because they are one of the world's most important commercial crops, grapevines are cultivated worldwide. They are used as raw materials of many consumer products such as juices, liquors, and wines [23]. UV-C exposure has been shown to efficiently induce the biosynthesis of resvertrol and its derivates in grapevine organs, including leaves and berries [24]–[27]. It is here speculated that a large of change in gene expression and metabolism should appear in grape leaves exposed to UV-C irradiation, based on results reported in these plants. However, none of these studies have analyzed the overall changes in gene expression or metabolism induced by UV-C in grape leaves and berries. DNA microarrays permit an overall view of gene expression involved in response to a particular stimulus in a rapid, efficient, and cost-effective manner [28]–[30]. Here, we focus on changes in gene expression of grape leaves in response to UV-C irradiation with Affymetrix *Vitis vinifera* (Grape) Genome Array, in order to understand the molecular basis of the response of grapevines to UV-C irradiation.

## Materials and Methods

### Plant materials and treatments

Vines of *V. vinifera* ‘Hongbaladuo’ were used in these experiments. They were grown in the vineyard at the Institute of Botany, Chinese Academy of Sciences, Beijing. According to the method described by Wang et al., healthy, mature (30-day old) leaves of similar size were detached from the shoot at 08:00–09:00 a.m. [27]. Leaf petioles were immediately inserted into the water in a bucket, and were then rapidly transferred from water buckets to triangular flasks containing ddH_2_O. All leaves were incubated in the dark at 25°C for half an hour. Then the leaf abaxial surfaces were exposed for 10 min to 6 W·m^−2^ UV-C irradiation provided by a UV-C lamp (Model ZW30S26W, Beijing Lighting Research Institute, China). The leaves were kept in the flasks until sampling. Samples were collected at 0, 6, and 12 h after the initiation of treatments. Each treatment was performed three times, and each replication consisted of six leaves. After sampling, the leaves were ground into powder in liquid nitrogen and stored at −80°C until analysis.

### RNA extraction, amplification, labeling, and hybridization

Total RNA was extracted from grape leaves using Trizol reagent (Invitrogen, Carlsbad, CA, U.S.) according to the manufacturer's instructions and digested with DNase I at 37°C for 15 min to remove any contaminating DNA. The RNA was cleaned with an RNeasy Kit (Qiagen, Hilden, Germany). RNA quantity and quality were determined using spectrophotometry and 1% formaldehyde denaturing gel electrophoresis. Samples with bright bands of ribosomal 28S to 18S RNA in a ratio >1.5∶1 were used for microarray analysis [28]–[30]. An Affymetrix Gene-Chip *V. vinifera* (Grape) Genome Array, which contains 15,700 probe sets covering 14,000 *V. vinifera* transcripts and 1,700 transcripts from other *Vitis* species, was used for microarray analysis. Hybridization, data capture, and analysis were performed by CapitalBio Corporation (Beijing, China), a service provider authorized by Affymetrix Inc. (Santa Clara, CA, U.S.). Briefly, 200 ng of total RNA was used for cDNA synthesis. This produced biotin-tagged cRNA with a MessageAmp^™^ Premier RNA Amplification Kit (Ambion). A 10 µg fragmented cRNA with control was hybridized to each GeneChip array at 45°C for 16 h (Affymetrix Gene Chip Hybridization Oven 640) according to the manufacturer's instructions. After hybridization, the GeneChip arrays were washed and stained with streptavidin phycoerythrinonan (SAPE) with an Affymetrix Fluidics Station 450 followed by scanning with an Affymetrix GeneChip Scanner 3000 7G. Microarray data processing produced microarray image files (CEL).

### Statistical analysis

The signal intensities of each feature were background adjusted and normalized via the quantile normalization performed by Robust Multichip Analysis (RMA) [31]. For defining differentially expressed (DE) probe sets, two group comparisons were performed for all probe sets between 0 and 6 h and between 0 and 12 h were performed via significance analysis of microarrays (SAM 2.10) [32]. Two filtering criteria were used: (1) *P*-value <0.05; (2) Fold change 12 h/0 h (6 h/0 h) ≧2 or ≤0.5. The lower confidence bound (LCB) of the 95% confidence interval of the fold changes was used [33]. The reliability of the comparison criteria was assessed by checking the false discovery rate (FDR) when permuting samples 1000 times. Probe sets that satisfied the criteria given above were chosen for further analysis. The DE prob sets were clustered with Gene Cluster 3.0. After log_2_ transformation, hierarchical clustering was performed on genes and arrays. Gene annotation and determination of functional categories were performed using data at PLEXdb (http://www.plexdb.org/) and based upon the findings reported by Deluc et al. [34].

### Validation of microarray data with real-time quantitative PCR (qRT-PCR)

Total RNA extraction was the same as that used for microarray analysis, as described above. The total RNA was treated with DNase I (Promega) to avoid DNA contamination. One microgram of RNA was reverse transcribed using the Superscript II reverse transcriptase (Invitrogen) with an oligo(dT)15 primer according to the manufacturer's instructions (Tiangen Biotech, Beijing, China). qRT-PCR experiments were conducted using Real Master Mix (SYBR Green) (Tiangen Biotech, Beijing, China). Reactions were carried out on a Step One Plus Real-Time PCR system (Life Technologies Corporation). The following standard thermal profile was used for all PCR experiments: 94°C for 5 min; 40 cycles of 95°C for 15 s, and 60°C for 60 s. Fluorescence signals were captured at the end of each cycle, and the melting curve analysis was performed from 68°C to 95°C to confirm the specificity of the PCR reaction. Probe set-specific primers were designed using Primer 5 software (Additional file S1). The amplification curves were analyzed with Biogazelle qbase^+^ software and the amplification efficiency of the primers was 90–110%. The data from the microarrays showed the actin gene (XM_002282480.2, 1606368_s_at) to be expressed stably in response to UV-C, the actin gene was used as the internal control to normalize all the qRT-PCR data. Analyses of qRT-PCR data was performed using the classic (1+E)^−ΔΔCT^ method (C_T_ is the threshold cycles of one gene, E is the amplification efficiency). ΔC_T_ is equal to the difference in threshold cycles for target (X) and reference (R) (C_T_,_X_–C_T,R_), while the ΔΔC_T_ is equal to the difference of ΔC_T_ for control (C, 0 h) and treatment (T, 6 or 12 h) (ΔC_T,T_–ΔC_T,C_). The amplification system (e.g., primer and template concentrations) was optimized, and the efficiency was close to 1. The amount of target, normalized to an endogenous reference and relative to a calibrator, was determined as follows: Amount of target  = 2^−ΔΔCT^.

### Gene ontology (GO) enrichment analysis of DE probe sets

The GO enrichement analysis of DE probe sets was performed with an online AgriGo tool (http://bioinfo.cau.edu.cn/agriGO) [35]. Specifically, the ‘Parametric Analysis of Gene Set Enrichment (PAGE)’ tool was used for analysis, and ‘vitis vinifera’ was entered as the species. A FDR multi-test adjustment was performed and the *P*-value <0.01. The other relative parameters were set as default.

## Results

### Expression and validation of differentially expressed probe sets (DE probe sets)

**Figure 1:**
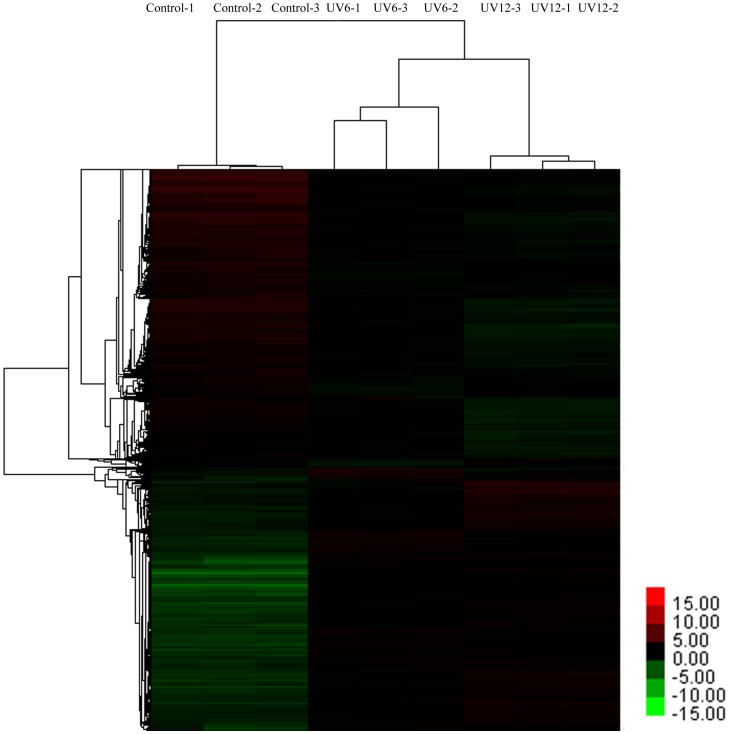
Average linkage hierarchical clustering analysis of the log_2_ signal values of the 5274 DE probe sets after UV-C. Control-1, control-2, and control-3 are three replications before UV-C treatment; UV6-1, UV6-2, and UV6-3 are three replications 6 h after UV-C treatment; UV12-1, UV12-2, and UV12-3 are three replications 12 h after UV-C treatment.

**Figure 2:**
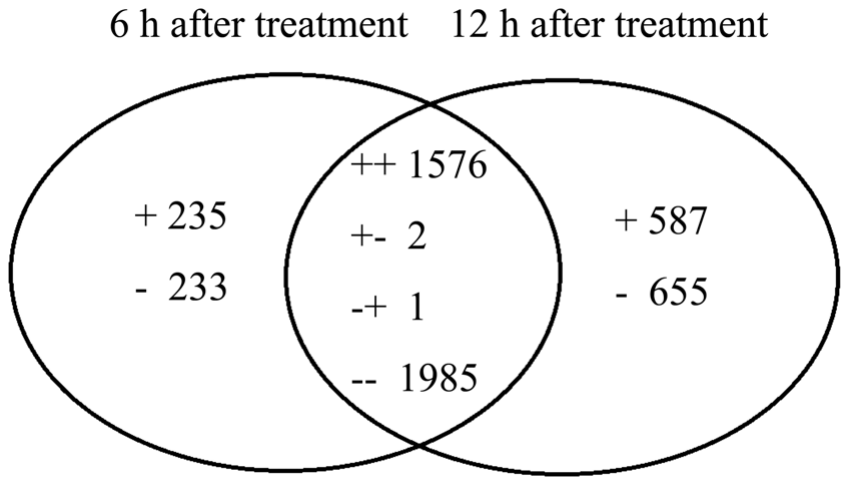
Venn diagram of differentially expressed transcripts (both identified and unknown) that were up- and down-regulated 6 and 12 h after UV-C treatment. The symbols “+” and “−” indicate up- and down-regulated transcripts. A total of 5274 transcripts were significantly (*P*<0.05) affected by UV-C treatment. There were 235 unique up-regulated transcripts 6 h after UV-C treatment; 587 unique up-regulated transcripts 12 h after UV-C treatment; 233 unique down-regulated transcripts 6 h after UV-C treatment; 655 unique down-regulated transcripts 12 h after UV-C treatment; 1576 transcripts were up-regulated both 6 and 12 h after UV-C treatment; 1985 transcripts were down-regulated both 6 and 12 h after UV-C treatment; 2 transcripts were up-regulated 6 h after UV-C treatment but down-regulated 12 h after UV-C treatment; 1 was down-regulated 6 h after UV-C treatment but up-regulated 12 h after UV-C treatment.

An Affymetrix *V. vinifera* Genome Array with 15,700 probe sets was used evaluate the transcriptomic changes in grape leaves in response to UV-C. The array data were averaged for three biological replicates and filtered as described in the Materials and methods section. We investigated the transcriptomic change in grape leaves at 6 and 12 h after the initiation of UV-C treatment, and compared it with control (i.e., before UV-C treatment, at 0 h after the initiation of UV-C treatment). A hierarchical clustering was prepared to represent the transcripts of all the DE probe sets at 3 replicates to compare the UV-C responsive transcriptomes (Figure 1). These results indicated that UV-C led to an intense change in the transcriptome. However, only slight differences were observed between 6 and 12 h after exposure to UV-C irradiation. According to the filtering criteria, a total of 5274 (about 33.59% of total probe sets) were defined to be DE probe sets at 6 or 12 h after UV-C treatment. These included 3564 probe sets that appeared at both 6 and 12 h after UV-C treatment, which represented 67.59% of the total DE probe sets. Among DE probe sets, 468 probe sets (8.88%) and 1242 probe sets (23.55%) were differentially expressed specifically at 6 and 12 h respectively (Figure 2). Among the total 5274 DE probe sets, 1576 showed an up-regulated trend and 1985 showed a down-regulated trend at both 6 and 12 h post-exposure to UV-C irradiation, and 3 showed the opposite trends at both points in time. Furthermore, 235 probe sets were uniquely up-regulated at 6 h after UV-C treatment and 233 were down-regulated. There were 587 probe sets that were uniquely up-regulated 12 h after UV-C treatment, and 655 probe sets were down-regulated.

In order to further confirm the results obtained from the microarray analyses, qRT-PCR assays were conducted on 25 probe sets sequences using specific primers (Additional file S1). The qRT-PCR profiles were analyzed using three biological replicates. Linear regression analyses displayed highly significant correlations (r^2^ = 0.951 for 6 h, and r^2^ = 0.933 for 12 h) between qRT-PCR and microarray results for the 25 evaluated probe sets (Additional file S2), confirming the validity of the microarray results.

### Functional analysis of probe sets responsive to UV-C irradiation

The results of AgriGO enrichment analysis showed 5274 DE probe sets to be enriched in 8 biological processes (Table 1). In general, the up-regulated DE-probe sets were found to be involved in amino acid and derivative metabolic and secondary metabolic processes and response to stimulus. The down-regulated DE-probe sets were enriched in photosynthetic processes. To further determine the pattern of regulation of UV-C response-related probe sets, the probe sets were annotated in PLEXdb (http://www.plexdb.org) and Munich Information Center for Protein (MIPS) sequences, and classified based on MIPS. A total of 2990 probe sets (about 56.70% of total DE probe sets) were functionally annotated and analyzed further. An additional 1002 probe sets (about 19% of total DE probe sets) matched genes with unknown functions or had unclear classifications (unclassified), and 1266 (about 24% of total DE probe sets) probe sets did not have any BLAST hits in public, non-redundant databases. Expression patterns and functional categories of the 2990 annotated probe sets are shown in Additional files S3–S6 and Table 2, respectively. Common probe sets were compared and the probe sets that were specifically expressed at 6 or 12 h after exposure to UV-C irradiation were analyzed based on functional classification.

**Table 1:** Functional enrichment analysis of DE probe sets.

GO Term	Onto	Number	Description	Z-score	
				6 h	12 h
GO:0006519	P	320	Cellular amino acid and derivative metabolic process	6.8	7.4
GO:0006950	P	290	Response to stress	6.6	6.3
GO:0009607	P	73	Response to biotic stimulus	5	4.7
GO:0019748	P	126	Secondary metabolic process	4.7	4.3
GO:0050896	P	472	Response to stimulus	4.7	4.8
GO:0051704	P	64	Multi-organism process	4.3	4.3
GO:0009056	P	212	Catabolic process	4.3	4.9
GO:0015979	P	81	Photosynthesis	−4.4	−7.5

Z-score is the statistical value in PAGE calculation. P represents biological processes. The positive.

values of Z-score indicate the corresponding biology process is up-regulated; The negative.

values of Z-score indicate the corresponding biology process is dowm-regulated.

**Table 2:** Functional categories of probe sets and expression pattern.

	Commonly regulated at 6 and 12 h		Uniquely regulated at 6 h		Uniquely regulated at 12 h	
	up	down	up	down	up	down
Metabolism	206	262	19	25	70	90
Energy	26	71	1	4	14	47
Storage protein	3	1	1	0	0	0
Cell cyecle and DNA processing	8	29	0	8	12	4
Transcription	90	125	24	12	21	33
Protein synthesis	20	59	1	2	92	15
Protein fate	110	109	12	16	46	50
Protein with binding function	32	35	2	2	13	18
Protein activity regulation	7	0	0	0	1	0
Transport regulation	126	122	21	18	41	38
Signal transduction	99	76	19	5	28	23
Cell rescue	105	70	9	11	6	30
Interaction with cellular environment	4	3	0	0	0	0
Plant/fungal specific systemic Sensing and response	42	66	4	4	6	18
Transposable elements	3	5	0	1	0	0
Cell fate	10	18	1	0	0	2
Development	3	4	1	1	4	3
Biogenesis of cellular component	17	47	7	4	7	9
Total	911	1102	122	113	361	380

### Common responsive probe sets 6 and 12 h post-exposure to UV-C irradiation

There were 911 annotated probe sets that were up-regulated at both 6 and 12 h after exposure to UV-C treatment compared with pre-exposure. They were divided into groups according to their putative involvement in different cellular events (Additional file S3). Of these, 206 (22.61%) were involved in metabolism, which contained the most probe sets. Among these probe sets, 41 were assigned to lower categories of secondary metabolism, including 13 probe sets representing stilbene synthase (up-regulated 8–700-fold). There were 126, 110, and 105 probe sets involved in transport regulation, protein fate, and cell rescue, respectively. Of these, 99 were related to signal transduction. These included serine/threonine kinase, leucine-like receptor kinase, chitin elicitor receptor kinase, N-acetyl-1-glutamate kinase, mitogen-activated protein kinase, MAP kinase, and MAPK kinase. Some 20 probe sets were involved in G-protein mediated signal transduction, and 24 probe sets were involved in Ca^2+^-mediated signal transduction. There were 90 probe sets associated with transcription, such as MYB family transcription factors, WRKY family transcription factors, and zinc finger family proteins. The probe set representing *VvMYB*14 (NM_001281203.1) was up-regulated by about 206-fold 6 h after exposure to UV-C irradiation, and it was still up-regulated 211-fold at 12 h. *WRKY57-like* (XM_002275540.1) and *ethylene responsive element binding factor* (XR_077949.1) were also strongly up-regulated 170- and 117-fold, respectively. Additional file S4 shows 1102 probe sets to be down-regulated at both 6 and 12 h after UV-C irradiation. Of these, 262 were involved in metabolism. Likewise, 48 probe sets were associated with secondary metabolism, including probe sets involved the in biosynthesis of alkaloids, porphyrins, lignins, and flavonoids. There were 125 probe sets associated with transcription, including MYB family genes, WRKY family genes, and others. *VvMYBPA1*, which regulates tannin biosynthesis, was among these probe sets. It was down-regulated by 2.7 and 3.13-fold 6 and 12 h after exposure to UV-C irradiation, respectively. There were 122 and 109 probe sets involved in transport regulation and protein fate, respectively. Moreover, 76 probe sets were involved in signal transduction, which included several protein kinases, probe sets associated with G-protein, small GTPase, Ca^2+^, fatty acid derivatives, polyphosphoinositde, and receptor enzyme-mediated signal transduction, and probe sets involved in transmembrane receptor protein tyrosine kinase and serine/threonine kinase signaling pathways. There was only one annotated gene that showed an opposite trend at 6 and 12 h after UV-C treatment. The probe set representing serine carboxy peptidase was up-regulated at 6 h and down-regulated at 12 h after the treatment.

### Probe sets specifically up- and down-regulated 6 h post-exposure to UV-C irradiation

As shown in Additional file S5, there were a total of 122 up-regulated probe sets identified, which were uniquely responsive to UV-C at 6 h post-exposure to UV-C irradiation. Of these, 24 probe sets were involved in transcription regulation, including two MYB family transcription factors, one WRKY DNA-binding protein, two bHLH DNA-binding proteins, and three zinc finger family proteins. There were 21 probe sets associated with transporters and 19 associated with metabolism. There were 19 signal transduction-related probe sets, including one NAD kinase, two MAP kinases, one shaggy-related kinase, one ras-related protein, three calcium-binding EF-hand family proteins, and one calcium-dependent protein kinase.

There were 113 down-regulated probe sets responding to UV-C uniquely at 6 h in grape leaves (Additional file S5). Of these, 25 probe sets were metabolism-related and involved in amino acid, pyrimidine nucleotide, phosphate, carbohydrate compounds, lipids, fatty acids, isoprenoid metabolism, and biosynthesis of phenylpropanoids. There were 18 probe sets associated with down-regulated transporters, including three sec14p-like phosphatidylinositol transfer family proteins, two sugar transporters, one amino acid transporter, and two H(+)-ATPases. Transcript regulatory probe sets included one GRA family transcription factor, one GAGA-binding transcriptional activator, and one transcriptional co-activator p15 (PC4) family protein.

### Probe sets specifically up- or down-regulated 12 h post-exposure to UV-C irradiation

In Additional file S6, a total of 361 up-regulated probe sets were found to respond to UV-C uniquely at 12 h. Of these, 25 probe sets were found to be associated with metabolism. There were 41, 28, and 21 probe sets involved in transport regulation, signal transduction, and transcription, respectively. There were 92 probe sets related to protein synthesis. These included probe sets associated with ribosomal proteins and translation-initiation-related proteins. Similarly, 46 probe sets were found to be related to protein fate. These contribute to protein folding and stabilization, targeting, sorting and translocation, and modification and degradation.

In contrast, 380 probe sets were down-regulated (Additional file S6). Of these, 90 probe sets were associated with metabolism and 65 were involved in protein synthesis and protein fate. There were 47 energy-related probe sets down-regulated at 12 h after treatment, and 38 down-regulated probe sets were found to be transporter-related, including the three phosphate translocator related proteins, three antiporters, one transport ATPase, and several ABC transporters.

## Discussion

UV-C irradiation disrupts cellular homeostasis in plants, severely retarding growth and development, sometimes causing death. Plants exposed to UV-C irradiation exhibit a characteristic set of cellular and metabolic responses [8]–[13]. The results of the present work showed that some genes were repressed and others were triggered after exposure to UV-C irradiation. In the leaves of *Vitis vinifera*, biological functions and regulatory networks of genes were coordinated and mobilized in response to UV-C irradiation. Based on these results, the discussion section focuses on the following factors.

### Antioxidant enzymes and defense response

**Figure 3:**
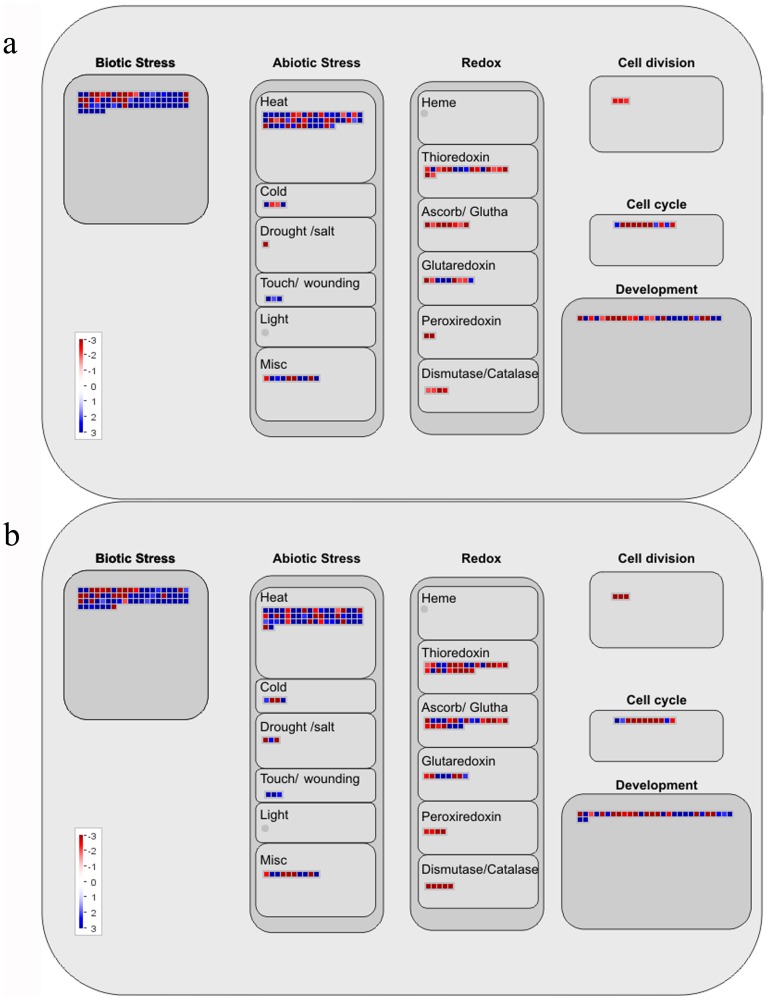
MapMan visualization of changes in the celluar responses pathway at (a) 6 h and (b) 12 h after UV-C treatment. Red box represent up-regulated probsets; Blue boxesrepresent down-regulated probsets.

The generation of reactive oxygen species (ROS) is a common feature of plant responses to different environmental stresses [36]. Oxidative stress due to UV-B exposure has previously been shown to increase the activity levels of different antioxidant enzymes, such as superoxide dismutase, ascorbate peroxidase, glutathione-S-transferases, glutathione-reductase, peroxidases. and catalases [37]–[39]. Although UV-C irradiation can cause DNA lesions and other damage to plants in a manner similar to UV-B, the response of plants to UV-C may manifest in different pathways [6], [40]. There is little available information regarding enzymatic antioxidant defense response to UV-C irradiation. In the present study, at 6 and 12 h after exposure to UV-C irradiation, the expression of two peroxidase probe sets and two thylakoid *ascorbate peroxidase* probe sets increased significantly. The expression of a stromal *ascorbate peroxidase* probe set was found to increase slightly at 6 and 12 h. The expression of most genes involved in oxygen and radical detoxification, such as superoxide dismutase, peroxidase, dehydroascorbate reductase, glutathione S-transferase, ascorbate peroxidase, glutathione peroxidase, and thioredoxin peroxidase, showed decreased expression (Table 3, Figure 3). This indicated that the present UV-C intensity could be stronger for grape leaves, or that the responses to UV-C may differ from UV-B in grape leaves. This is worth studying further. In addition, other genes contributing to stress defense, such as *ß-1,3-glucanase* and *chitinase* probe sets, were greatly up-regulated at 6 (67.98-fold) and 12 h (273.06-fold) after exposure to UV-C irradiation (Table 3, Figure 3).

**Table 3:** Genes involved in stress defense.

Probe set ID	Fold change at 6 h	Fold change at 12 h	Annotation
1609321_at	9.85	21.65	Peroxidase superfamily protein
1615967_at	3.41	8.64	Peroxidase precursor
1621336_at	1.83	2.70	Stromal ascorbate peroxidase
1609231_at	1.73	2.48	Thylakoidal ascorbate peroxidase
1620826_s_at	1.65	2.30	Thylakoidal ascorbate peroxidase
1616657_at	0.49	0.56	Superoxide dismutase [Cu-Zn]
1622739_at	0.48	0.48	Peroxidase superfamily protein
1609478_s_at	0.84	0.46	Class III peroxidase
1611203_at	0.64	0.45	Dehydroascorbate reductase 1
1615206_s_at	0.51	0.45	Glutathione S-transferase
1611993_at	0.61	0.43	Ascorbate peroxidase 3
1608089_at	0.50	0.42	Glutathione peroxidase
1611871_at	0.63	0.41	Dehydroascorbate reductase
1620356_x_at	0.60	0.35	Glutathione S-transferase
1618599_at	0.37	0.33	Superoxide dismutase
1609324_at	0.58	0.32	Glutathione S-transferase
1619210_at	0.47	0.29	Superoxide dismutase [Cu-Zn]
1614776_a_at	0.51	0.28	Superoxide dismutase [Cu-Zn]
1617515_at	0.48	0.25	L-ascorbate peroxidase
1614361_at	0.53	0.24	Peroxidase superfamily protein
1608433_at	0.29	0.11	Thioredoxin superfamily protein
1614204_at	0.30	0.09	Thioredoxin superfamily protein
1613132_s_at	0.15	0.07	Peroxidase superfamily protein
1612707_at	0.14	0.07	Superoxide dismutase
1618920_at	0.07	0.05	Peroxidase superfamily protein
1608586_at	0.08	0.04	Peroxidase superfamily protein
1613461_s_at	241.76	341.8	Class IV chitinase
1611710_at	169.87	263.66	Class IV chitinase
1613999_x_at	55.75	78.07	Chitinase A
1617192_at	107.00	68.16	Class IV chitinase
1608864_s_at	43.94	55.17	Acidic endochitinase precursor
1618373_at	25.89	35.31	Chitinase A
1607557_at	5.18	31.96	Class IV chitinase
1621319_s_at	4.24	17.00	Class IV chitinase
1613871_at	13.92	11.82	Chitinase
1617430_s_at	4.50	7.05	Basic chitinase
1620505_at	3.50	4.67	Chitinase class I
1608262_at	2.15	3.66	Class I extracellular chitinase
1611876_s_at	3.33	3.13	Acidic endochitinase precursor
1606625_at	3.76	2.61	Class IV chitinase
1612050_at	2.08	1.00	Chitinase A
1621583_at	0.34	0.23	Chitinase-like protein 2
1619916_s_at	55.41	273.06	ß -1,3-glucanase 3
1615595_at	67.98	114.52	ß -1,3-glucanase
1620063_at	39.61	75.38	ß -1,3-glucanase 1
1610722_at	8.03	10.36	ß -1,3-glucanase 1
1618425_at	7.36	6.66	ß -1,3-glucanase 3
1619828_at	0.94	0.38	ß -1,3-glucanase 2
1618409_at	0.29	0.17	ß -1,3-glucanase-like protein

### Secondary metabolism related to phenylpropanoid (PAL) pathway

**Figure 4:**
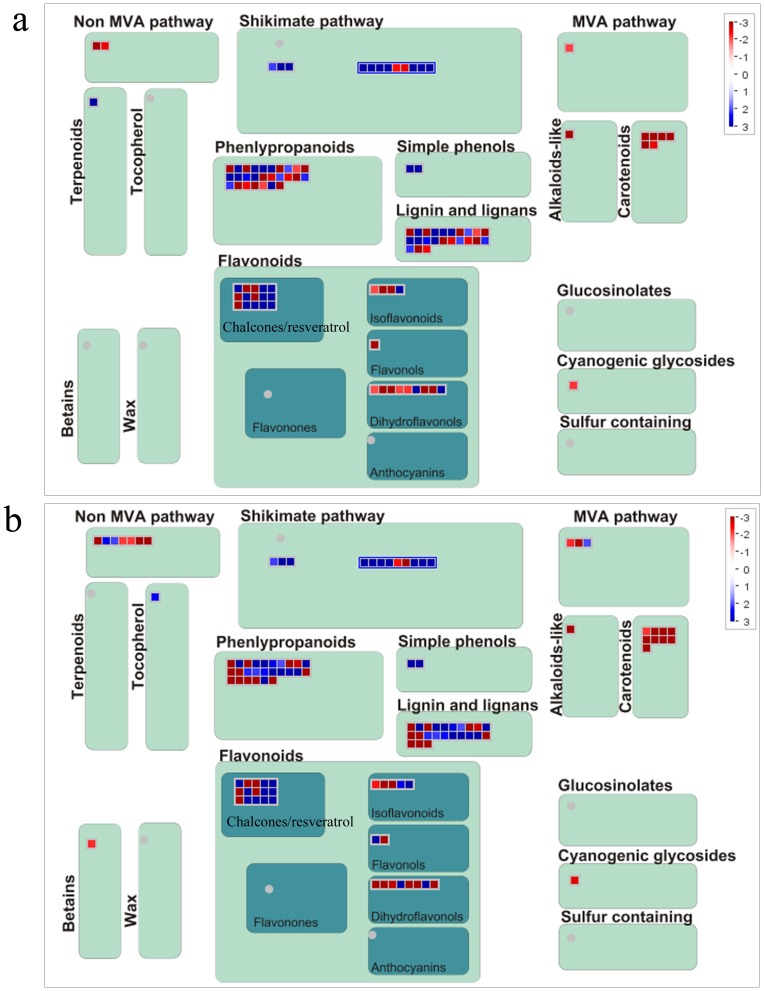
MapMan visualization of changes in the secondary metabolism pathway at (a) 6 h and (b) 12 hafter UV-C treatment. Red boxesrepresent up-regulated probsets; Blue boxesrepresent down-regulated probsets. Probsets for *PAL, C4H* and *4CL* are covered in phenlypropanoids pathway; probsets for *CHS*, *STS* and *resveratrol O-glucosyltransferase* are covered in the chalcones/resveratrol pathway.

UV-C displayed a significant inductive effect on secondary metabolism in grapevine leaves, especially on the PAL pathway (Table 4, Figure 4). Phenol is an important nonenzymatic compound. It functions by scavenging ROS to protect plants. Flavonoids are antioxidant molecules that act as free radical scavengers and contribute to the protection of plant components (such as chloroplasts and other organelles) from damage caused by UV-B irradiation [41]–[42]. However, whether flavonoids provide a similar response to UV-C irradiation is not clear. The results of the microarray performed here showed that four probe sets representing *CHS*, encoding the key enzyme for the synthesis of flavonoids, did not increase after UV-C treatment. In contrast, they were down-regulated about 2 to 10-fold (Table 4). Except anthocyanidin 3-O-glucosyltransferase and flavonol synthase were up-regulated 75 and 10-fold respectively, most of enzymes, such as flavanone 3-hydroxylase, dihydroflavonol-4-reductase, leucoanthocyanidin reductase 2, and anthocyanidin reductase, were down-regulated (Table 4, Figure 4). These enzymes were involved in isoflavonoid, dihydroflavonol and flavonol biosythsis.

**Table 4:** Genes involved in the biosynthesis of resveratrol and flavonoids.

Probe set ID	Fold change at 6 h	Fold changeat 12 h	Annotation
1609696_x_at	539.05	753.74	Stilbene synthase 1
1610850_at	515.92	707.8	Stilbene synthase 1
1620964_s_at	465.19	646.25	Stilbene synthase 1
1611190_s_at	360.37	489.72	Stilbene synthase 1
1610824_s_at	371.21	387.27	Stilbene synthase 2
1612804_at	227.87	236.81	Resveratrol synthase
1622638_x_at	224.51	224.8	Resveratrol synthase
1609697_at	306.96	200.09	Stilbene synthase 4
1608009_s_at	182.29	153.71	Resveratrol synthase 2
1616575_at	7.55	27.51	Resveratrol synthase
1614621_at	23.26	26.37	Stilbene synthase 1
1610070_at	11.91	17.84	Stilbene synthase
1606750_at	6.84	8.28	Stilbene synthase 3
1619011_at	0.49	0.48	Chalcone synthases
1606663_at	0.23	0.17	Chalcone synthase
1607732_at	0.11	0.09	Chalcone synthase
1617019_at	0.09	0.09	Chalcone synthase
1610206_at	6.47	7.9	Phenylalanine ammonia-lyase 2
1619642_at	0.28	0.31	Phenylalanine ammonia-lyase
1613113_at	0.26	0.22	Phenylalanine ammonia-lyase
1610821_at	10.71	20.52	Cinnamate-4-hydroxylase
1616191_s_at	7.72	14.31	Cinnamate-4-hydroxylase
1609307_at	8.96	7.22	4-coumarate:CoA ligase 1
1606753_at	1.44	2.64	4-coumarate–CoA ligase-like
1619320_at	3.19	2.03	4-coumarate:CoA ligase 3
1607228_at	1.19	3.25	Resveratrol/hydroxycinnamic acid O-glucosyltransferase
1608579_at	1.02	2.43	Resveratrol/hydroxycinnamic acid O-glucosyltransferase
1620342_at	133.44	211.13	Caffeic acid O-methyltransferase
1607475_s_at	56.11	90.11	Caffeic acid O-methyltransferase
1621563_x_at	16.04	13.2	Caffeic acid O-methyltransferase
1612124_at	16.1	13.07	Caffeic acid O-methyltransferase
1616434_s_at	6.53	6.09	Caffeic acid O-methyltransferase
1619682_x_at	3.32	2.99	Caffeic acid O-methyltransferase
1614191_s_at	0.78	2.24	Caffeic acid O-methyltransferase-like
1619450_s_at	2.55	2.89	Caffeic acid 3-O-methyltransferase
1615085_at	0.69	2.12	Caffeic acid 3-O-methyltransferase 1
1615401_at	89.65	75.24	Anthocyanidin 3-O-glucosyltransferase
1618155_at	2.94	2.15	Anthocyanidin 3-O-glucosyltransferase 6
1621051_at	2.56	1.64	Anthocyanidin 3-O-glucosyltransferase 2
1609876_at	0.33	0.43	Anthocyanidin 5,3-O-glucosyltransferase
1618389_at	0.47	0.38	Anthocyanin 5-O-glucoside-4″′-O-malonyltransferase
1614045_at	29.06	37.72	Ferulic acid 5-hydroxylase 1
1608791_at	1.78	10.38	Flavonol synthase
1618551_at	0.07	0.05	Flavonol synthase
1610780_at	4.14	3.71	Shikimate kinase 1
1617079_at	0.42	0.42	Shikimate kinase like 1
1612989_at	0.39	0.25	Shikimate kinase like 1
1614643_at	1.92	3.27	Caffeoyl-CoA O-methyltransferase
1611897_s_at	0.48	0.36	Caffeoyl-CoA O-methyltransferase
1607939_at	0.54	0.33	Caffeoyl-CoA O-methyltransferase
1607607_s_at	0.46	0.55	Flavanone 3-hydroxylase
1607739_at	0.13	0.19	Flavanone 3-hydroxylase
1611847_at	0.09	0.09	Flavonoid 3′,5′-hydroxylase
1607760_at	0.07	0.06	Flavonoid 3′,5′-hydroxylase
1616437_at	0.3	0.54	Dihydroflavonol-4-reductase
1615174_s_at	0.49	0.43	Leucoanthocyanidin reductase 2
1612134_at	0.64	0.29	Anthocyanidin reductase
1619986_s_at	0.13	0.17	UDP-glucose:flavonoid 3-O-glucosyltransferase
1621418_at	0.22	0.08	UDP-glucose flavonoid 3-O-glucosyltransferase
1615481_at	0.09	0.1	Cytochrome B5 isoform D
1614485_at	0.05	0.08	Putative anthranilate N-hydroxycinnamoyl/benzoyltransferase
1618112_at	0.14	0.14	Putative anthocyanidin-3-glucoside rhamnosyltransferase
1612436_s_at	13.45	13.86	Isoflavone reductase-like protein 3
1611389_at	0.19	0.12	Isoflavone reductase-like protein 6
1610923_a_at	1.31	2.56	Isoflavone reductase-like protein 5
1618991_s_at	0.23	0.22	Isoflavone reductase-like protein 6
1617421_at	8.05	10.78	Isoflavone reductase-like protein 3
1615912_at	10.09	10.89	Chalcone-flavanone isomerase family protein
1620424_at	0.21	0.18	Chalcone-flavanone isomerase family protein
1616977_at	4.98	3.87	Putative iron/ascorbate-dependent oxidoreductase
1609765_s_at	0.09	0.11	Leucoanthocyanidin dioxygenase
1614441_at	75.67	174.22	Leucoanthocyanidin dioxygenase-like
1607805_s_at	14.12	3.84	Flavonoid 3′-monooxygenase

Res is a nonflavonoid phenol present in the tissues and organs of several plant families, such as Arachaceae, Vitaceae, and Pinaceae [43]. Previous studies have reported the biological activity of this polyphenol, i.e., estrogenic activity, cardiovascular protective effects, neuroprotective capacity, and cancer chemopreventive activity [44]. The ability of UV-C to induce Res accumulation in grape leaves and berries has attracted special attention [24], [45]–[46]. Cantos et al. irradiated grape berries with UV-C, and the resveratrol concentration was 11 times higher than that of controls. Bonomelli et al. showed that grape leaves treated with UV-C irradiation accumulated Res and that the concentration reached 400 µg/g DW even though no Res was detected in leaves exposed to natural sunlight alone. Some studies have indicated that the accumulation of Res is caused by up-regulation of *STS* expression [24], [26], [47]. The results of the microarray performed here show 13 *STS* probe sets to be up-regulated by 8.28- to 753.74-fold at 6 and 12 h after exposure to UV-C irradiation (Table 4, Figure 4). The different levels of expression of *STS* probe sets here suggest that different *STS* genes are regulated differently in response to UV-C irradiation. Dai et al. reported that, in grape leaves, individual *STS* genes respond differentially to powdery mildew infection [48]. In the present study, the expression of one *PAL* probe set, two *C4H* probe sets, three *4CL* probe sets, and two *resveratrol O-glucosyltransferase* probe sets was also up-regulated (Table 4, Figure 4). It is here suggested that, in grape leaves, although both flavonoids and Res are produced through the same phenylpropanoid pathway, they respond to UV-C in different ways. Some transcription factors may up-regulate the expression of *PAL*, *C4H*, *4CL*, *STS*, and *resveratrol O-glucosyltransferase*.

### Transcription factors (TFs)

**Figure 5:**
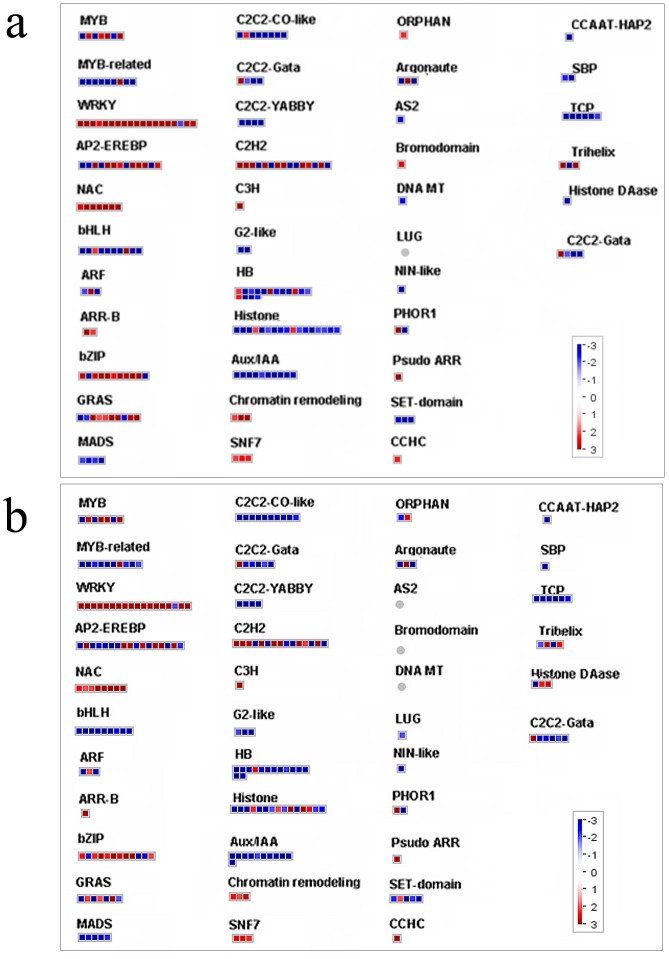
MapMan visualization ofchanges in the transcription factor pathway at (a) 6 h and (b) 12 h after UV-C treatment. Red boxesrepresent up-regulated probsets; Blue boxesrepresent down-regulated probsets.

The regulation of gene expression plays a fundamental role in plant response to environmental stimuli. Transcription factors (TFs) belonging to the MYB, ERF, bZIP, and WRKY families have been linked to a suite of mechanisms leading to defense and stress responses [49]–[50]. For this reason, the present discussion focused more on differentially expressed probe sets belonging to these gene families (Table 5, Figure 5). A few members of the R2R3-MYB family have been implicated in plant stress response to cold, UV-B, and wounding. These have been preliminarily shown to regulate plant secondary metabolism [51]. R2R3-MYBs have been established as positive and negative regulators of the biosynthetic enzymes required for the production of phenylpropanoids and flavonoids [52]–[53]. For example, *AtMYB4* encodes a protein similar to *Am*MYB308, which represses one of the key targets genes encoding C4H. Some studies have reported that R2R3-MYBs are involved in the regulation of flavonoid biosynthesis. In grape berries, the MYB proteins act together with bHLH protein and probably WDR protein to control anthocyanin and/or proanthocyanidin synthesis [54]–[58]. In the present experiment, 18 probe sets representing MYB transcription factors were expressed very differently in grape leaves after exposure to UV-C irradiation, especially, *Vv*MYB14 and *Vv*MYB4 (XM_002285157.2). These were up-regulated 211.7-fold and 113.5-fold, respectively, in UV-C treated grape leaves (Table 5, Figure 5). It is possible that *VvMYB14* and *VvMYB4* regulate resveratrol and flavonoid synthesis. In a previous study, VvMYBPA1 was found to specifically regulate proanthocyanidin (PA) synthesis, positively regulating both of the PA branch enzymes leucoanthocyanidin reductase (LAR) and anthocyanidin reductase (ANR). This causes the formation of PA and may regulate the entire general flavonoid pathway, inducing the promoters of the general flavonoid pathway genes *CHI* (*chalconeisomerase*), *F3′5 ′H* (*flavonoid 3′,5′-hydroxylase*) and *LDOX* (*leucoanthocyanidin dioxygenase*) [58]. The down-regulation of *VvMYBPA1* suggests a decrease in the biosynthesis of PAs and even total flavonoids. In this study, the gene *VvMYBPA1* was down-regulated more than 2.5-fold at both 6 and 12 h after exposure to UV-C irradiation (Table 5, Figure 5). *LAR* and *ANR* were down-regulated, but the expression levels of various members of *CHS*, *CHI*, *F3′5 ′H*, and *LDOX* were either up- or down-regulated (Table 4, Figure 4).

**Table 5:** Transcription factors responsive to UV-C irradiation.

Probe set ID	Fold change at 6 h	Fold change at 12 h	Annotation
1620319_s_at	206.42	211.77	VvMyb14
1618260_s_at	73.61	133.5	*Vitis vinifera* transcription factor Myb4-like
1622064_at	71.49	91.66	VvMyb14
1613545_at	3.53	4.91	MYB transcription factor
1619386_at	2.57	1.71	MYB transcription factor R3 type
1618514_at	1.36	3.12	MYB transcription factor
1609021_at	0.54	2.78	Vitis vinifera transcription factor Myb59-like
1612264_at	0.32	0.44	MYB transcription factor
1610512_at	0.31	0.43	MYB transcription factor
1617092_at	0.28	0.26	MYB transcription factor
1613239_at	0.26	0.22	MYB transcription factor
1614416_at	0.19	0.14	MYB transcription factor
1617998_at	0.18	0.22	*Vitis vinifera* R2R3 Myb transcription factor
1618884_at	0.09	0.08	MYB transcription factor
1613486_at	0.09	0.07	*Vitis vinifera* transcription factor MYB1R1-like
1611920_at	0.04	0.04	MYB transcription factor
1616094_at	0.37	0.32	VvMYBPA1
1621872_s_at	0.13	0.14	*Vitis vinifera* transcription factor MYB1R1-like
1613407_at	9.94	6.29	*Vitis vinifera* probable WRKY transcription factor 33-like
1610775_s_at	170.22	156.51	*Vitis vinifera* probable WRKY transcription factor 57-like
1607465_at	52.85	39.2	*Vitis vinifera* probable WRKY transcription factor 57-like
1622778_at	43.54	79.12	*Vitis vinifera* WRKY-type DNA binding protein 1 mRNA
1606659_s_at	39.34	30.29	*Vitis vinifera* probable WRKY transcription factor 65-like
1609130_at	31.36	9.12	*Vitis vinifera* probable WRKY transcription factor 48-like
1609636_at	22.79	14.78	*Vitis vinifera* probable WRKY transcription factor 33-like
1614806_s_at	17	11.83	*Vitis vinifera* probable WRKY transcription factor 40-like
1610064_at	12.84	12.68	*Vitis vinifera* probable WRKY transcription factor 33-like
1611285_s_at	11.04	4.93	*Vitis vinifera* WKRY protein
1622399_at	10.72	4.46	*Vitis vinifera* WKRY protein
1616623_at	8.14	5.05	*Vitis vinifera* probable WRKY transcription factor 28-like
1611550_at	6.68	7.04	*Vitis vinifera* probable WRKY transcription factor 46-like
1612649_s_at	4.68	3.99	*Vitis vinifera* probable WRKY transcription factor 7-like
1611650_at	4.55	3.66	*Vitis vinifera* probable WRKY transcription factor 7-like
1619424_at	2.85	7.28	*Vitis vinifera* probable WRKY transcription factor 11-like
1613318_at	2.64	1.75	*Vitis vinifera* WKRY protein
1622333_at	2.51	0.93	*Vitis vinifera* probable WRKY transcription factor 23-like
1620175_at	0.48	0.49	*Vitis vinifera* WRKY transcription factor 44-like
1619311_at	117.45	74.79	Ethylene responsive element binding factor 1
1619585_at	13.1	12.88	Ethylene-responsive factor-like protein 1
1621552_at	7.02	12.62	Ethylene-responsive transcriptional coactivator-like protein
1609683_at	3.33	6.54	Ethylene-responsive element binding protein
1610300_at	2.42	3.41	Ethylene responsive element binding protein
1606975_at	2.08	1.19	Putative ethylene response factor ERF3a
1608511_at	0.74	0.27	Ethylene responsive element binding factor 5
1617671_s_at	3.01	1.8	Ethylene response factor domain protein 9
1611910_s_at	0.69	0.25	Similar to putative ethylene response factor

The WRKY proteins share a DNA binding domain, which contains an invariant WRKYGQK sequence. The WRKY TFs super-family is involved in a diverse set of biological functions including pathogen defense, abiotic stress responses, and plant development [59]. In this study, 18 probe sets representing WRKY factors showed increased expression in response to UV-C treatment, especially WRKY57-like (Table 5, Figure 5). This suggests that these factors protect the grapevines from potentially damaging UV-C irradiation.

It has been established that ethylene and ethylene response factor (ERF) proteins play important regulatory roles in plant pathogen resistance and abiotic stress [60]–[61]. In grapevines, ethylene plays an important role in berry development and ripening, including the regulation of gene expression for anthocyanin biosynthesis and accumulation [62]. It was here found that several genes, annotated as ERFs and ethylene responsive proteins, are strongly expressed (the maximum may reach 75-fold) post-exposure to UV-C treatment (Table 5, Figure 5). In this way, ethylene may affect the response of leaves to UV-C irradiation.

## Conclusions

In conclusion, the study identified UV-C irradiation-regulated genes using Affymetrix Grape Genome Array and qRT-PCR techniques. The leaf transcriptome of the grapevines was affected by UV-C irradiation. The responsive probe sets were found to belong to a large number of important factors and biological pathways, such as cell rescue (i.e., antioxidant enzymes), protein fate (i.e., HSPs), secondary metabolism (i.e., *STS* were up-regulated 750-fold), transcription factors, and signal transduction. These results may provide novel insight into the grape leaf response to UV-C and may have considerable implications for further study and application.

## Supporting Information

Additional file S1 **Probe set-specific primers for RT-PCR.**
(DOC)

Additional file S2 **Linear correlation analysis (r^2^ = 0.951 for 6 h and r^2^ = 0.933 for 12 h) between qRT-PCR and microarray results for 25 probe sets.** X: log_2_ fold change value from qRT-PCR data; Y: log_2_ fold change value from microarray data.
(DOCX)

Additional file S3 **Probe sets commonly up-regulated at 6 and 12 h after exposure to UV-C irradiation.**
(DOCX)

Additional file S4 **Probe sets commonly down-regulated at 6 and 12 h after exposure to UV-C irradiation.**
(DOCX)

Additional file S5 **Probe sets specifically up- and down-regulated at 6 h after exposure to UV-C treatment.**
(DOCX)

Additional file S6 **Probe sets specifically up- and down-regulated at 12 h after exposure to UV-C treatment.** This information is available free of charge via the Internet at http://pubs.acs.org.
(DOCX)
