# Supplementary material for: Transcriptomic Analysis of Grape (Vitis vinifera L.) Leaves after Exposure to Ultraviolet C Irradiation
Source: PLoS One. 2014 Dec 2;9(12):e113772. doi: 10.1371/journal.pone.0113772 (PMC4252036; doi:10.1371/journal.pone.0113772)
Supplement: Additional file S1 — Probe set-specific primers for RT-PCR. (DOC) [file pone.0113772.s001.doc]

**Additional file S1** Gene-specific primers for RT-PCR

| Probe set ID | Gene name description | Function categories |  | Primer |
| --- | --- | --- | --- | --- |
| 1608705_at | UDP-glucosyltransferas | Metabolism | F | TCCACAACCCCTTCTTCATTC |
|  |  |  | R | GCAGTTGGATGTTTCTTGACG |
| 1618478_at | UDP-arabinose 4-epimerase 1 | Metabolism | F | CATCAAGAAGCCATAGCCAG |
|  |  |  | R | GTTTTCAGCGACCCAAGTAAG |
| 1607475_s_at | Caffeic acid O-methyltransferase | Metabolism | F | TCCACCACAATGACTTTTCC |
|  |  |  | R | GATATGTTCACAAGCGTTCC |
| 1610724_at | Fructose-bisphosphate aldolase | Engery | F | GAAAGGGGTGGATATGCTGT |
|  |  |  | R | GGATGGATCTGTAAAATGGC |
| 1613407_at | WRKY33-like | Transcription | F | GGGGAGTTTTGGATTCTCAG |
|  |  |  | R | CCTTAGGCTCTTCCTTGGTTC |
| 1620319_s_at | VvMyb14 | Transcription | F | CAAGCCCCTTCCCTCATATTTC |
|  |  |  | R | GTGCTGTCATTGGCAGTAG |
| 1607465_at | WRKY57-like | Transcription | F | CGTGGTCACAACTTATGAAGGC |
|  |  |  | R | CATCTGGCTCAAGATATGCTC |
| 1618260_s_at | Myb4-like | Transcription | F | CCCCATCTTCTCACTAACCG |
|  |  |  | R | TGGCATGGAGTTCTGGTATG |
| 1613141_at | NAC domain containing protein 42 | Transcription | F | CTTGTAGGGTTTTATCTGCGGC |
|  |  |  | R | ATCTCCCATGTTAGTGCTGACTCT |
| 1621076_at | 60S ribosomal protein L6 | Protein aynthesis | F | GTTGATTATCTTGGCTGGGAG |
|  |  |  | R | TTTACACGCCTTAGAGGAACAC |
| 1619528_s_at | Heat shock protein 70 | Protein fate | F | ACTTGAGATTTCTTGGTTGGG |
|  |  |  | R | CGTTGCTTATGGTGCTGCTGT |
| 1618590_at | ABC-2 type transporter family protein | Transport regulation | F | AAGAGGCAGAAAACCAGAAGG |
|  |  |  | R | GCAATAAAGGCTGAAGGAACAC |
| 1606881_at | Mitogen-activated protein kinase 3 | Signal transduction | F | GTCACAAGACGCACAATAGGC |
|  |  |  | R | GAAGGTTTCACCCAGTAGAGG |
| 1609397_at | GTP cyclohydrolase II | Signal transduction | F | TACTGTGGAAGCCAATGAAGAGC |
|  |  |  | R | GTGGGACTCTGCCAGAAACTG |
| 1620074_at | Calmodulin-like 11 | Signal transduction | F | GTAAAGGAAACAGACGCAGAAG |
|  |  |  | R | TCATCACATGCCTCAACTCAG |
| 1610447_at | Phospholipase C 2 | Signal transduction | F | CCTCCAGTGTTTTCGTTTTC |
|  |  |  | R | TCCCCTCCAGATTTCTATGC |
| 1620080_at | Receptor kinase 2 | Signal transduction | F | AGGTTCAGTTTTGCGAGTGC |
|  |  |  | R | CCTCCCATCCCTGTTTAGATC |
| 1618920_at | Peroxidase superfamily protein | Cell rescue | F | ATTCCTCCCAGAGCTAAAAGC |
|  |  |  | R | CAGCAAATCCACCCCTAATG |
| 1618599_at | Superoxide dismutase | Cell rescue | F | AATACCAACCAAACCCAAGG |
|  |  |  | R | GGAGGAAAAGAGCAGAGGAAG |
| 1616933_at | Glutathione S-transferase | Cell rescue | F | CCTTTGAAGACGGAGACCTG |
|  |  |  | R | ATCTGGGCATATCAGTTGAGTCC |
| 1613871_at | Chitinase | Cell rescue | F | CAGAGGAGGATTCTAAGCGTG |
|  |  |  | R | ATGGATACTGGGTGTTGGTCTC |
| 1611710_at | Class IV chitinase | Cell rescue | F | ATTCGTATTCTGGGTTCGGC |
|  |  |  | R | GAGAGGCACCATTGATTTCTTC |
| 1622360_at | Nitrilase 4 | Plant/fungal specific systemic sensing and response | F | TGGGTAGCCACAATGAGACAC |
|  |  |  | R | GGGTAATCTTTCCTCCGACAG |
| 1622147_at | Ethylene-forming enzyme | Plant / fungal specific systemic sensing and response | F | ACAGGAAGTGTATGGAGCAGAGG |
|  |  |  | R | GTAGATGGCGGAGGAAGAAG |
| 1607001_at | Tubulin beta-1 chain | Biogenesis of cellular component | F | GGAGGCTGAGAATTGTGATTGC |
|  |  |  | R | GATGGGAAGACGGAGAAAGTG |
| Reference gene | Actin 1 |  | F | CTTGCATCCCTCAGCACCTT |
|  |  |  | R | TCCTGTGGACAATGGATGGA |
