# Supplementary material for: Transcriptomic Analysis of Grape (Vitis vinifera L.) Leaves after Exposure to Ultraviolet C Irradiation
Source: PLoS One. 2014 Dec 2;9(12):e113772. doi: 10.1371/journal.pone.0113772 (PMC4252036; doi:10.1371/journal.pone.0113772)
Supplement: Additional file S2 — Linear correlation analysis (r2 = 0.951 for 6 h and r2 = 0.933 for 12 h) between qRT-PCR and microarray results for 25 probe sets. X: log2 fold change value from qRT-PCR data; Y: log2 fold change value from microarray data. (DOCX) [file pone.0113772.s002.docx]

**Additional file S2**  Linear correlation analysis (r^2^ = 0.951 for 6 h and r^2^ = 0.933 for 12 h) between qRT-PCR and microarray results for 25 genes. X: log_2_ fold change value from qRT-PCR data; Y: log_2_ fold change value from microarray data.

**12 h**
