# Supplementary material for: Transcriptomic Analysis of Grape (Vitis vinifera L.) Leaves after Exposure to Ultraviolet C Irradiation
Source: PLoS One. 2014 Dec 2;9(12):e113772. doi: 10.1371/journal.pone.0113772 (PMC4252036; doi:10.1371/journal.pone.0113772)
Supplement: Additional file S3 — Probe sets commonly up-regulated at 6 and 12 h after exposure to UV-C irradiation. (DOCX) [file pone.0113772.s003.docx]

**Additional file S3** Genes up-regulated commonly at 6 and 12 h after UV-C treatment

|  |  | Fold change | |  |
| --- | --- | --- | --- | --- |
| Category | Probe set ID | 6 h | 12 h | Gene name description |
| Metabolism | 1609696_x_at | 539.05 | 753.74 | Stilbene synthase 1 |
|  | 1610850_at | 515.92 | 707.8 | Stilbene synthase 1 |
|  | 1620964_s_at | 465.19 | 646.25 | Stilbene synthase 1 |
|  | 1610824_s_at | 371.21 | 387.27 | Stilbene synthase 2 |
|  | 1611190_s_at | 360.37 | 489.72 | Stilbene synthase 1 |
|  | 1609697_at | 306.96 | 200.09 | Stilbene synthase 4 |
|  | 1612804_at | 227.87 | 236.81 | Resveratrol synthase |
|  | 1622638_x_at | 224.51 | 224.8 | Resveratrol synthase |
|  | 1608009_s_at | 182.29 | 153.71 | Resveratrol synthase |
|  | 1622369_at | 135.88 | 116.6 | RmlC-like cupins superfamily protein |
|  | 1620342_at | 133.44 | 211.13 | O-methyltransferase |
|  | 1609234_at | 112.25 | 205.59 | Polyphenol oxidase |
|  | 1615401_at | 89.65 | 75.24 | Anthocyanidin 3-O-glucosyltransferase |
|  | 1614680_at | 82.45 | 157.12 | Isopropyl malate isomerase large subunit 1 |
|  | 1614441_at | 75.67 | 174.22 | 2-oxoglutarate (2OG) and Fe(II)-dependent oxygenase superfamily protein |
|  | 1615595_at | 67.98 | 114.52 | Class I beta-1,3-glucanase |
|  | 1616445_at | 57.62 | 61.09 | Cinnamoyl CoA reductase-like protein |
|  | 1607475_s_at | 56.11 | 90.11 | O-methyltransferase 1 |
|  | 1619916_s_at | 55.41 | 273.06 | Beta-1,3-glucanase 3 |
|  | 1622651_at | 54.16 | 170.5 | Polyphenol oxidase |
|  | 1612393_s_at | 52.16 | 122.6 | D-isomer specific 2-hydroxyacid dehydrogenase family protein |
|  | 1611611_at | 45.79 | 359.23 | Germin 3 |
|  | 1611027_at | 41.78 | 42.43 | Beta-fructofuranosidase 5 |
|  | 1620063_at | 39.61 | 75.38 | Beta-1,3-glucanase 1 |
|  | 1622396_at | 32.57 | 29.37 | 12-oxophytodienoate reductase 2 |
|  | 1620628_at | 32.1 | 33.32 | Neutral invertase |
|  | 1622012_at | 31.33 | 15.95 | Plant invertase/pectin methylesterase inhibitor superfamily protein |
|  | 1616944_at | 31.31 | 37.95 | Serine hydrolase |
|  | 1607926_at | 31.11 | 17.58 | Auxin-responsive family protein |
|  | 1614045_at | 29.06 | 37.72 | Ferulic acid 5-hydroxylase 1 |
|  | 1617740_at | 28.63 | 9.28 | Ripening-related P-450 enzyme |
|  | 1618373_at | 25.89 | 35.31 | Acidic endochitinase precursor |
|  | 1620469_at | 25.61 | 102.47 | S-adenosyl-L-methionine: 2,7,4'-trihydroxyisoflavanone 4'-O- methyltransferase |
|  | 1607430_at | 25.58 | 28.14 | Myo-inositol oxygenase 5 |
|  | 1617293_s_at | 25.51 | 15.33 | Methylenetetrahydrofolate reductase family protein |
|  | 1610382_at | 25.28 | 4.65 | Jasmonic acid carboxyl methyltransferase |
|  | 1619034_at | 24.81 | 55.65 | Cytochrome P450 |
|  | 1609744_at | 24.56 | 18.09 | Acyl-CoA N-acyltransferases (NAT) superfamily protein |
|  | 1614621_at | 23.26 | 26.37 | Stilbene synthase 1 |
|  | 1617361_at | 23.1 | 22.7 | Phosphoserine aminotransferase |
|  | 1621543_x_at | 21.81 | 19.33 | 12-oxophytodienoate reductase 2 |
|  | 1607713_s_at | 21.76 | 267.54 | Carboxyesterase 17 |
|  | 1617466_at | 21.27 | 17.63 | Threonine aldolase 2 |
|  | 1610356_at | 18.81 | 10.67 | Similar to AT5g12010/F14F18_180 |
|  | 1619357_at | 18.77 | 17.84 | 3-deoxy-D-arabino-heptulosonate-7-phosphate synthase |
|  | 1618478_at | 18.48 | 14.06 | UDP-arabinose 4-epimerase 1 |
|  | 1608225_s_at | 16.88 | 42.78 | Alpha-hydroxynitrile lyase |
|  | 1609712_at | 16.54 | 4.29 | Cytochrome P450 |
|  | 1612124_at | 16.1 | 13.07 | Caffeic acid O-methyltransferase |
|  | 1621563_x_at | 16.04 | 13.2 | Caffeic acid O-methyltransferase |
|  | 1622542_a_at | 15.43 | 25.58 | Oxalyl-CoA decarboxylase (Fragment) |
|  | 1621068_at | 15.37 | 46.99 | Protease/crotonase family protein |
|  | 1610806_at | 15.04 | 43.94 | Laccase/Diphenol oxidase family protein |
|  | 1610059_at | 14.92 | 12.94 | Threonine aldolase 1 |
|  | 1610410_at | 14.64 | 25.52 | UDP-Glycosyltransferase superfamily protein |
|  | 1616188_at | 14.31 | 12.64 | CXE carboxylesterase |
|  | 1607805_s_at | 14.12 | 3.84 | Cytochrome P450 superfamily protein |
|  | 1609985_at | 13.94 | 14.25 | ATP-citrate lyase A-2 |
|  | 1609932_at | 13.85 | 11.61 | Arogenate dehydratase 6 |
|  | 1621307_at | 13.49 | 10.34 | Prephenate dehydratase 1 |
|  | 1612436_s_at | 13.45 | 13.86 | Isoflavone reductase-like protein 3 |
|  | 1610008_s_at | 12.86 | 29.3 | 3-hydroxyisobutyryl-coenzyme A hydrolase |
|  | 1620283_s_at | 12.78 | 13.54 | Alpha-amylase |
|  | 1611211_at | 12.66 | 14.7 | 3-deoxy-D-arabino-heptulosonate-7-phosphate synthase |
|  | 1612706_at | 12.54 | 16.36 | Laccase/Diphenol oxidase family protein |
|  | 1607262_at | 12.28 | 6.16 | Alpha/beta-Hydrolases superfamily protein |
|  | 1606577_at | 12.19 | 11.7 | Hydroperoxide lyase 1 |
|  | 1614207_at | 12.18 | 55.2 | Glutamine-dependent asparagine synthase 1 |
|  | 1612482_at | 12.02 | 14.02 | Pantothenate kinase 2 |
|  | 1619065_at | 11.91 | 9.79 | Ribosome-binding factor A |
|  | 1610070_at | 11.91 | 17.84 | Stilbene synthase |
|  | 1613947_at | 11.58 | 22.5 | Non-specific phospholipase C3 |
|  | 1608705_at | 11.56 | 22.81 | UDP-glucosyltransferase |
|  | 1617286_at | 11.51 | 28.16 | ATP-dependent caseinolytic (Clp) protease |
|  | 1618362_s_at | 11.11 | 11.65 | ATP-citrate lyase A-2 |
|  | 1610821_at | 10.71 | 20.52 | Cinnamate-4-hydroxylase |
|  | 1616966_at | 10.7 | 13.61 | Glutamine-fructose-6-phosphate transaminase |
|  | 1615912_at | 10.09 | 10.89 | Chalcone-flavanone isomerase family protein |
|  | 1621983_s_at | 9.9 | 5.79 | Beta-1,6-N-acetylglucosaminyltransferase family protein |
|  | 1615399_at | 9.5 | 11.64 | L-O-methylthreonine resistant 1 |
|  | 1611135_at | 9.48 | 24.01 | Methyl esterase 17 |
|  | 1611682_at | 9.33 | 10.48 | ACT-like superfamily protein |
|  | 1616853_at | 9.31 | 7.74 | AMP-dependent synthetase and ligase family protein |
|  | 1621305_s_at | 9.15 | 8.4 | Indole-3-butyric acid response 1 |
|  | 1609307_at | 8.96 | 7.22 | 4-coumarate:CoA ligase 1 |
|  | 1614440_at | 8.81 | 7.8 | Class-II DAHP synthetase family protein |
|  | 1616014_at | 8.8 | 19.57 | FAD/NAD(P)-binding oxidoreductase family protein |
|  | 1607663_at | 8.74 | 5.62 | Pyridoxal phosphate (PLP)-dependent transferases superfamily protein |
|  | 1615062_a_at | 8.49 | 11.69 | L-O-methylthreonine resistant |
|  | 1607505_s_at | 8.42 | 22.92 | Aldehyde dehydrogenase 2B4 |
|  | 1621405_at | 8.36 | 7.06 | 3-deoxy-d-arabino-heptulosonate 7-phosphate synthase |
|  | 1618322_at | 8.19 | 11.46 | Aspartate aminotransferase |
|  | 1608624_at | 8.09 | 11.16 | Cellulose synthase like E1 |
|  | 1617421_at | 8.05 | 10.78 | Isoflavone reductase-like protein 3 |
|  | 1615657_at | 8.03 | 9.19 | PHD zinc finger superfamily protein |
|  | 1610722_at | 8.03 | 10.36 | Beta-1,3-glucanase 1 |
|  | 1613159_at | 7.73 | 4.65 | Indole-3-butyric acid response 1 |
|  | 1616191_s_at | 7.72 | 14.31 | Cinnamate-4-hydroxylase |
|  | 1619990_at | 7.68 | 44.74 | Dehydroquinate dehydratase |
|  | 1616575_at | 7.55 | 27.51 | Resveratrol synthase |
|  | 1618425_at | 7.36 | 6.66 | Beta-1,3-glucanase 3 |
|  | 1619268_at | 7.19 | 11.98 | Aspartate aminotransferase |
|  | 1611488_at | 7.04 | 7.37 | Alpha/beta-Hydrolases superfamily protein |
|  | 1611895_at | 7.03 | 8.44 | Chorismate mutase 1 |
|  | 1621397_at | 6.93 | 18.75 | Aspartate aminotransferase 3 |
|  | 1620412_at | 6.9 | 6.26 | Acetyl-CoA carboxylase 1 |
|  | 1615714_at | 6.84 | 8.72 | UDP-Glycosyltransferase superfamily protein |
|  | 1606750_at | 6.84 | 8.28 | Stilbene synthase 3 |
|  | 1616783_at | 6.78 | 17.67 | Aldehyde dehydrogenase 2B4 |
|  | 1610126_at | 6.76 | 15.93 | Aspartate aminotransferase 3 |
|  | 1611875_at | 6.74 | 6.62 | Methionine gamma-lyase |
|  | 1615803_s_at | 6.57 | 6.48 | Alpha/beta-Hydrolases superfamily protein |
|  | 1616434_s_at | 6.53 | 6.09 | Caffeic acid O-methyltransferase |
|  | 1610206_at | 6.47 | 7.9 | Phenylalanine ammonia-lyase 2 |
|  | 1610468_at | 6.37 | 10.62 | Phosphoglycerate dehydrogenase-like protein |
|  | 1608156_at | 6.34 | 7.55 | Trehalase 1 |
|  | 1620732_at | 6.32 | 7.59 | Glutamine-fructose-6-phosphate transaminase |
|  | 1619698_at | 6.19 | 10.46 | UDP-Glycosyltransferase superfamily protein |
|  | 1622736_at | 6.13 | 9.73 | D-3-phosphoglycerate dehydrogenas |
|  | 1610218_s_at | 5.85 | 4.77 | UDP-glucose 6-dehydrogenase family protein |
|  | 1613619_at | 5.73 | 3.48 | Cytochrome P450, family 81 |
|  | 1620905_at | 5.69 | 4.47 | UDP-glucose 6-dehydrogenase family protein |
|  | 1615742_at | 5.56 | 7.37 | RNA 3'-terminal phosphate cyclase/enolpyruvate transferase |
|  | 1620347_at | 5.54 | 6.01 | Glycosyltransferase family 61 protein |
|  | 1607492_at | 5.54 | 7.04 | Ripening-related protein |
|  | 1613514_s_at | 5.34 | 9.37 | Alkaline alpha galactosidase II |
|  | 1610427_at | 5.33 | 40.21 | Myo-inositol oxygenase 1 |
|  | 1609635_s_at | 5.25 | 9.69 | MutT domain protein-like |
|  | 1616513_at | 5.09 | 6.5 | Laccase/Diphenol oxidase family protein |
|  | 1611542_at | 5.07 | 5.8 | Polyphenol oxidase |
|  | 1616977_at | 4.98 | 3.87 | 2-oxoglutarate (2OG) and Fe(II)-dependent oxygenase superfamily protein |
|  | 1618806_at | 4.96 | 15.66 | Triacylglycerol lipase like protein |
|  | 1615365_at | 4.86 | 3.86 | RELA/SPOT homolog 3 |
|  | 1612364_at | 4.59 | 2.77 | Sphingoid base hydroxylase 1 |
|  | 1609270_at | 4.52 | 3.07 | ACT domain repeat 8 |
|  | 1609153_at | 4.39 | 5.27 | AMP-dependent synthetase and ligase family protein |
|  | 1607697_at | 4.38 | 4.33 | 2-oxoglutarate (2OG) and Fe(II)-dependent oxygenase superfamily protein |
|  | 1612836_at | 4.31 | 5.9 | Vacuolar invertase 2 |
|  | 1622855_at | 4.19 | 4.46 | Adenine phosphoribosyl transferase 1 |
|  | 1620664_at | 4.18 | 2.26 | Glycosyl hydrolase family 35 protein |
|  | 1610780_at | 4.14 | 3.71 | Shikimate kinase 1 |
|  | 1606479_at | 4.02 | 6.44 | NAD(P)-linked oxidoreductase superfamily protein |
|  | 1613647_at | 4 | 4.24 | Germin-like protein 5 |
|  | 1622282_at | 3.9 | 13.34 | pfkB-like carbohydrate kinase family protein |
|  | 1609451_at | 3.84 | 4.46 | Pheophytinase |
|  | 1611851_at | 3.84 | 3.79 | Phosphofructokinase 2 |
|  | 1622507_at | 3.54 | 3.61 | NAD(P)-binding Rossmann-fold superfamily protein |
|  | 1610310_at | 3.51 | 5.07 | Diphosphoinositol polyphosphate phosphohydrolase |
|  | 1622752_at | 3.46 | 17.01 | Serine hydrolase |
|  | 1614479_at | 3.46 | 2.74 | Arginine decarboxylas |
|  | 1618096_s_at | 3.43 | 3.6 | Ripening-related protein |
|  | 1616294_s_at | 3.41 | 2.07 | Nudix hydrolase homolog 10 |
|  | 1619682_x_at | 3.32 | 2.99 | Caffeic acid O-methyltransferase |
|  | 1615974_at | 3.32 | 3.86 | Ripening-related protein-like |
|  | 1615478_at | 3.2 | 8.09 | Galacturonic acid reductase |
|  | 1619320_at | 3.19 | 2.03 | 4-coumarate:CoA ligase 3 |
|  | 1607149_at | 3.13 | 5.51 | Aspartate aminotransferase |
|  | 1611872_at | 3.03 | 3.34 | 3-ketoacyl-CoA synthase 11 |
|  | 1614918_at | 3.02 | 3.52 | Glycoside hydrolase family |
|  | 1614153_at | 3.01 | 4.97 | Glucose-6-phosphate 1-dehydrogenase |
|  | 1621826_at | 3 | 5.75 | Similar to BON1-associated protein |
|  | 1617174_at | 2.97 | 9.09 | Carboxyesterase 17 |
|  | 1615182_at | 2.96 | 3.01 | UDP-glucose 6-dehydrogenase family protein |
|  | 1618155_at | 2.94 | 2.15 | UDP-Glycosyltransferase superfamily protein |
|  | 1617911_at | 2.92 | 3.43 | Ribophorin I |
|  | 1617576_at | 2.87 | 4.1 | Alpha/beta-Hydrolases superfamily protein |
|  | 1618329_at | 2.79 | 2.61 | Mannosyl-oligosaccharide 1,2-alpha-mannosidase |
|  | 1608379_at | 2.74 | 7.97 | 2-oxoglutarate (2OG) and Fe(II)-dependent oxygenase superfamily protein |
|  | 1612386_at | 2.7 | 2.09 | Cytochrome P450 |
|  | 1614216_at | 2.61 | 2.34 | Rhamnose biosynthesis 1 |
|  | 1616909_at | 2.61 | 2.64 | Oxysterol-binding family protein |
|  | 1609867_s_at | 2.58 | 3.04 | Haloacid dehalogenase-like hydrolase (HAD) superfamily protein |
|  | 1614285_at | 2.56 | 6.27 | Cytochrome P450 |
|  | 1619450_s_at | 2.55 | 2.89 | O-methyltransferase 1 |
|  | 1621834_at | 2.54 | 2.34 | Lipid phosphate phosphatase 3 |
|  | 1613188_at | 2.54 | 4.1 | Alpha-amylase-like 3 |
|  | 1616399_s_at | 2.53 | 2.19 | Arginine decarboxylase |
|  | 1611460_at | 2.48 | 3.01 | UDP-glucose 6-dehydrogenase family protein |
|  | 1612389_at | 2.45 | 6.08 | NADH glutamate dehydrogenase |
|  | 1609475_at | 2.42 | 2.54 | Pyridoxal-5'-phosphate-dependent enzyme family protein |
|  | 1608630_at | 2.4 | 2.25 | UDP-glucose 6-dehydrogenase family protein |
|  | 1618595_at | 2.38 | 9.57 | NAD(P)-binding Rossmann-fold superfamily protein |
|  | 1617124_at | 2.38 | 2.89 | Chloroplast beta-amylase |
|  | 1610836_at | 2.37 | 4.61 | Acyl-CoA oxidase 1 |
|  | 1615772_s_at | 2.36 | 3.3 | UDP-xylose synthase 4 |
|  | 1617090_at | 2.32 | 2.19 | Enoyl-CoA hydratase/isomerase A |
|  | 1607457_at | 2.29 | 4 | Glutamate decarboxylase |
|  | 1620891_at | 2.29 | 2.89 | Chloroplast beta-amylase |
|  | 1617324_at | 2.27 | 3.42 | Pyridoxal phosphate (PLP)-dependent transferases superfamily protein |
|  | 1618782_at | 2.27 | 4.33 | Citrate synthase |
|  | 1613260_at | 2.22 | 2.01 | Enoyl-CoA hydratase/isomerase A |
|  | 1606798_at | 2.21 | 2.89 | Glutamine synthetase cytosolic isozyme 1 |
|  | 1608650_at | 2.16 | 3.27 | Copper amine oxidase family protein |
|  | 1608968_at | 2.13 | 3.27 | Acyl-CoA N-acyltransferases (NAT) superfamily protein |
|  | 1608863_s_at | 2.13 | 3.49 | Pyridoxal phosphate (PLP)-dependent transferases superfamily protein |
|  | 1612913_at | 2.13 | 3.55 | Citrate synthase 3 |
|  | 1611453_at | 2.11 | 2.39 | Mitochondrial ferredoxin 1 |
|  | 1616326_at | 2.11 | 2.11 | Prephenate dehydratase 1 |
|  | 1609402_at | 2.11 | 3.41 | Sucrose synthase 3 |
|  | 1619039_at | 2.09 | 2.19 | Carnitine racemase like protein |
|  | 1620557_at | 2.09 | 3.61 | Phosphoglycerate mutase, |
|  | 1616059_at | 2.06 | 2.17 | Ubiquitin family protein |
|  | 1616519_s_at | 2.02 | 2.1 | Indole-3-butyric acid response 10 |
|  | 1613917_s_at | 2.02 | 2.63 | Cytidine/deoxycytidylate deaminase family protein |
|  | 1618720_at | 2.02 | 2.15 | Similar to glycoside hydrolase family 47 protein |
|  | 1616766_at | 2.01 | 3.86 | Acyl-CoA oxidases |
| Engery | 1607193_at | 143.97 | 114.67 | Alternative oxidase |
|  | 1619850_at | 67.09 | 77.63 | Aconitase 3 |
|  | 1615827_at | 64.48 | 108.24 | Alternative oxidase 1B |
|  | 1615814_at | 19.47 | 54.78 | Glyceraldehyde-3-phosphate dehydrogenase |
|  | 1617922_at | 18.83 | 14.14 | PLAT/LH2 domain-containing lipoxygenase family protein |
|  | 1610509_at | 10.83 | 18.89 | Alpha/beta-Hydrolases superfamily protein |
|  | 1606774_at | 5.11 | 12.23 | Pyruvate kinase, cytosolic isozyme |
|  | 1612546_at | 3.22 | 7.77 | Malate dehydrogenase |
|  | 1622391_at | 3.21 | 3.18 | Protein kinase superfamily protein |
|  | 1613796_at | 3.11 | 5.35 | Pyruvate dehydrogenase complex E1 alpha subunit |
|  | 1610724_at | 2.99 | 4.18 | Fructose-bisphosphate aldolase |
|  | 1621359_at | 2.89 | 3.57 | Pyruvate dehydrogenase E1 beta subunit isoform 2 |
|  | 1609000_at | 2.86 | 4.46 | Transketolase family protein |
|  | 1606716_at | 2.59 | 5.14 | Dihydrolipoamide acetyltransferase, long form protein |
|  | 1614221_at | 2.3 | 3.73 | Aldolase-type TIM barrel family protein |
|  | 1621402_a_at | 2.25 | 3.59 | Cytochrome c-2 |
|  | 1621034_at | 2.24 | 2.08 | Phosphoenolpyruvate carboxykinase 1 |
|  | 1616757_at | 2.23 | 3.05 | Cytochrome c-2 |
|  | 1609684_at | 2.21 | 4.82 | Similar to pyruvate decarboxylase |
|  | 1617448_at | 2.21 | 5.61 | Malate dehydrogenase |
|  | 1611103_at | 2.17 | 3.46 | Phosphoenolpyruvate carboxylase 1 |
|  | 1614105_at | 2.13 | 2.94 | Pyruvate dehydrogenase complex E1 alpha subunit |
|  | 1621682_s_at | 2.09 | 7.14 | Pyruvate decarboxylase 1 |
|  | 1611322_at | 2.09 | 5.88 | Pyruvate decarboxylase 1 |
|  | 1617606_at | 2.02 | 2.01 | Aconitase 3 |
|  | 1620652_at | 2.01 | 2.59 | Ubiquinol-cytochrome C reductase complex 6.7 kDa protein |
| Storage protein | 1607320_s_at | 44.98 | 166.76 | RmlC-like cupins superfamily protein |
|  | 1610600_at | 14.68 | 7.72 | Seed specific protein Bn15D18B |
|  | 1610403_s_at | 8.22 | 3.26 | Alpha carbonic anhydrase 7 |
| Cell cyecle and DNA processing | 1614635_at | 10.88 | 8.73 | P-loop containing nucleoside triphosphate hydrolases superfamily protein |
|  | 1614535_at | 3.4 | 3.57 | DYNAMIN-like 1E |
|  | 1612220_at | 2.43 | 3.32 | Cyclin C-like protein |
|  | 1606487_at | 2.31 | 4.01 | ATPase |
|  | 1619063_at | 2.27 | 2.98 | Histone H2A protein 9 |
|  | 1616336_at | 2.24 | 2.63 | Root hair initiation protein root hairless 1 (RHL1) |
|  | 1616140_at | 2.18 | 2.11 | Cyclin family protein |
|  | 1608321_at | 2.13 | 2.46 | Chromatin remodeling 8 |
| Transcription | 1620319_s_at | 206.42 | 211.77 | R2R3 Myb14 transcription factor |
|  | 1610775_s_at | 170.22 | 156.51 | WRKY57-like |
|  | 1619311_at | 117.45 | 74.79 | Ethylene responsive element binding factor 1 |
|  | 1621876_at | 103.45 | 196.82 | NAC domain protein NAC1 |
|  | 1618260_s_at | 73.61 | 133.5 | Vitis vinifera transcription factor Myb4-like |
|  | 1622064_at | 71.49 | 91.66 | R2R3 Myb14 transcription factor |
|  | 1607465_at | 52.85 | 39.2 | WRKY DNA-binding protein 75 |
|  | 1622778_at | 43.54 | 79.12 | WRKY DNA-binding protein 75 |
|  | 1606659_s_at | 39.34 | 30.29 | WRKY DNA-binding protein 65 |
|  | 1611013_at | 36.64 | 34.1 | C2H2 and C2HC zinc fingers superfamily protein |
|  | 1609555_at | 35.63 | 37.96 | NAC (No Apical Meristem) domain Transcriptional regulator superfamily protein |
|  | 1609172_at | 33.13 | 48.79 | NAC (No Apical Meristem) domain Transcriptional regulator superfamily protein |
|  | 1609130_at | 31.36 | 9.12 | WRKY DNA-binding protein 48 |
|  | 1613136_at | 27.55 | 8.98 | Similarity to protein At2g34600 |
|  | 1614332_s_at | 23.8 | 26.11 | C2H2 and C2HC zinc fingers superfamily protein |
|  | 1609636_at | 22.79 | 14.78 | WRKY DNA-binding protein 33 |
|  | 1613141_at | 20.46 | 37 | NAC domain containing protein 42 |
|  | 1614806_s_at | 17 | 11.83 | WRKY DNA-binding protein 40 |
|  | 1619585_at | 13.1 | 12.88 | Ethylene-responsive factor-like protein 1 |
|  | 1610064_at | 12.84 | 12.68 | WRKY DNA-binding protein 33 |
|  | 1611285_s_at | 11.04 | 4.93 | WRKY DNA-binding protein 11 |
|  | 1622399_at | 10.72 | 4.46 | WRKY DNA-binding protein 11 |
|  | 1609265_at | 10.58 | 12.8 | GATA transcription factor 5 |
|  | 1613770_s_at | 9.96 | 6.44 | Jasmonate-zim-domain protein 1 |
|  | 1613407_at | 9.94 | 6.29 | WRKY DNA-binding protein 33 |
|  | 1622863_at | 8.81 | 20.86 | Zinc finger protein |
|  | 1616623_at | 8.14 | 5.05 | WRKY DNA-binding protein 71 |
|  | 1609403_at | 7.08 | 13.09 | Nudix hydrolase homolog 2 |
|  | 1621552_at | 7.02 | 12.62 | Ethylene-responsive transcriptional coactivator-like protein |
|  | 1611550_at | 6.68 | 7.04 | Similar to AtWRKY41 |
|  | 1612620_at | 6.31 | 8.82 | BTB and TAZ domain protein 1 |
|  | 1618998_at | 5.73 | 3.82 | RAV transcription factor |
|  | 1616263_s_at | 5.71 | 5.53 | Similarity to protein ref:NP_566215.1 (A.thaliana) |
|  | 1613740_at | 4.99 | 5.8 | C2H2 and C2HC zinc fingers superfamily protein |
|  | 1613707_at | 4.85 | 9.82 | Basic region/leucine zipper motif 53 |
|  | 1609107_at | 4.76 | 3.79 | Salt tolerance zinc finger |
|  | 1612649_s_at | 4.68 | 3.99 | WRKY DNA-binding protein 15 |
|  | 1618771_s_at | 4.65 | 3.34 | Related to ABI3/VP1 2 |
|  | 1608918_at | 4.61 | 6.34 | Zinc finger protein 622 |
|  | 1611569_at | 4.6 | 2.07 | GRAS family transcription factor |
|  | 1609812_at | 4.55 | 4.9 | Zinc finger (CCCH-type) family protein |
|  | 1611650_at | 4.55 | 3.66 | WRKY DNA-binding protein 15 |
|  | 1617254_at | 4.46 | 2.01 | NAC domain containing protein 38 |
|  | 1613270_at | 4.25 | 4.55 | Nonsense-mediated mRNA decay NMD3 family protein |
|  | 1609662_at | 4.14 | 3.28 | Scarecrow-like 1 |
|  | 1607082_at | 4.11 | 6.32 | BTB and TAZ domain protein 1 |
|  | 1617798_at | 4.05 | 3.13 | Basic-leucine zipper (bZIP) transcription factor family protein |
|  | 1614931_at | 4.01 | 4.32 | Homeodomain-like superfamily protein |
|  | 1615100_at | 3.67 | 2.82 | Transcription initiation factor IIB |
|  | 1609933_at | 3.65 | 4.62 | C2H2 and C2HC zinc fingers superfamily protein |
|  | 1618763_at | 3.61 | 3.25 | Basic region/leucine zipper motif 53 |
|  | 1612485_at | 3.54 | 2.32 | TGA-type basic leucine zipper protein TGA2.2 |
|  | 1609177_at | 3.54 | 2.75 | Helicase domain-containing protein |
|  | 1613545_at | 3.53 | 4.91 | MYB transcription factor GmMYB62 |
|  | 1622559_at | 3.49 | 2.3 | Basic region/leucine zipper motif 60 |
|  | 1608328_s_at | 3.35 | 3.25 | Basic region/leucine zipper motif 53 |
|  | 1619573_at | 3.33 | 2.92 | Salt tolerance zinc finger |
|  | 1609683_at | 3.33 | 6.54 | Ethylene-responsive element binding protein |
|  | 1617518_at | 3.04 | 3.73 | C2H2 and C2HC zinc fingers superfamily protein |
|  | 1606655_at | 3.03 | 3.42 | Argonaute family protein |
|  | 1615964_at | 3 | 2.09 | NAC domain containing protein 57 |
|  | 1608182_at | 2.99 | 2.59 | WUSCHEL related homeobox 13 |
|  | 1619855_at | 2.89 | 3.58 | SUPERMAN-like zinc finger protein |
|  | 1619492_at | 2.87 | 5.39 | Splicing factor-like protein |
|  | 1622676_s_at | 2.85 | 2.55 | Tubby like protein 10 |
|  | 1618534_at | 2.85 | 2.66 | G/HBF-1 |
|  | 1619424_at | 2.85 | 7.28 | WRKY DNA-binding protein 21 |
|  | 1621515_at | 2.82 | 2.41 | Basic-leucine zipper (bZIP) transcription factor family protein |
|  | 1617778_at | 2.77 | 3.65 | RNA 3'-terminal phosphate cyclase-like protein |
|  | 1621920_at | 2.73 | 2.61 | Transcription factor AP2D23-like |
|  | 1609054_s_at | 2.73 | 3.2 | Heat shock transcription factor A1E |
|  | 1614289_at | 2.67 | 2.15 | Salt tolerance zinc finger |
|  | 1620621_at | 2.61 | 2.47 | NAC (No Apical Meristem) domain Transcriptional regulator superfamily protein |
|  | 1608315_at | 2.56 | 2.89 | DREB and EAR motif protein 2 |
|  | 1618932_s_at | 2.53 | 2.75 | Transcription initiation factor IIB-2 |
|  | 1611583_at | 2.52 | 2.79 | Integrase-type DNA-binding superfamily protein |
|  | 1618356_a_at | 2.47 | 2.31 | Transcription initiation factor IIB |
|  | 1607383_at | 2.45 | 3.44 | DNA-directed RNA polymerase |
|  | 1609796_at | 2.44 | 2.65 | RNA polymerase I specific transcription initiation factor RRN3 protein |
|  | 1610300_at | 2.42 | 3.41 | Ethylene responsive element binding protein |
|  | 1621816_at | 2.41 | 2.77 | Elongator protein 2 |
|  | 1609514_at | 2.39 | 2.34 | Basic region/leucine zipper motif 53 |
|  | 1612570_at | 2.34 | 2.43 | Transcription elongation factor (TFIIS) family protein |
|  | 1607063_s_at | 2.31 | 2.7 | Similar to splicing factor-like protein |
|  | 1607120_at | 2.21 | 2.56 | NAC domain containing protein 2 |
|  | 1615701_at | 2.18 | 2.37 | Splicing factor-like protein |
|  | 1608709_at | 2.14 | 2.15 | GRAS family transcription factor |
|  | 1618784_at | 2.12 | 2.16 | Polynucleotidyl transferase, ribonuclease H-like superfamily protein |
|  | 1616996_at | 2.01 | 2.53 | DEAD-box protein 3 |
|  | 1607362_at | 2 | 2.51 | Ribosomal RNA processing Brix domain protein |
| Protein synthesis | 1615947_at | 6.01 | 7.12 | Glutamyl/glutaminyl-tRNA synthetase |
|  | 1620210_at | 4.95 | 7.07 | Glutamyl/glutaminyl-tRNA synthetase |
|  | 1622565_at | 4.36 | 4.22 | Glutamyl-tRNA reductase |
|  | 1611812_at | 4.3 | 4.29 | Eukaryotic translation initiation factor 2 beta subunit |
|  | 1616704_at | 3.82 | 4.91 | Class II aminoacyl-tRNA and biotin synthetases superfamily protein |
|  | 1613168_at | 3.49 | 4.36 | Glycyl-tRNA synthetase / glycine--tRNA ligase |
|  | 1620427_at | 3.06 | 4.73 | Ribosomal protein L1p/L10e family |
|  | 1621076_at | 3.02 | 4.75 | 60S ribosomal protein L6 |
|  | 1620143_at | 2.57 | 2.66 | Elongation factor G/III/V family protein |
|  | 1609146_at | 2.54 | 2.49 | Translation initiation factor IF2/IF5 |
|  | 1607562_at | 2.37 | 2.58 | Ribosomal protein L12/ ATP-dependent Clp protease adaptor protein ClpS family protein |
|  | 1622436_at | 2.26 | 3.95 | Ribosomal protein L7Ae/L30e/S12e/Gadd45 family protein |
|  | 1619176_at | 2.23 | 2.16 | Translation initiation factor IF2/IF5 |
|  | 1609822_at | 2.2 | 3.58 | Ribosomal protein S8e family protein |
|  | 1613631_at | 2.18 | 3.38 | Ribosomal protein L7Ae/L30e/S12e/Gadd45 family protein |
|  | 1608093_at | 2.18 | 3.33 | Ribosomal protein S14p/S29e family protein |
|  | 1622158_s_at | 2.13 | 2.97 | Ribosomal protein S28 |
|  | 1617446_at | 2.07 | 3.06 | Ribosomal protein L7Ae/L30e/S12e/Gadd45 family protein |
|  | 1612990_at | 2.03 | 3.1 | Ribosomal protein L11 family protein |
|  | 1616219_at | 2 | 3.76 | 40S ribosomal protein S24 |
| Protein fate | 1611445_at | 62.01 | 51.97 | Protein kinase-like protein |
|  | 1606741_at | 22.12 | 14.01 | Plant U-box 22 |
|  | 1612310_at | 20.99 | 24.96 | Heat shock factor |
|  | 1615800_at | 17.38 | 11.12 | Heat shock transcription factor |
|  | 1620702_a_at | 14.91 | 17.02 | Haloacid dehalogenase-like hydrolase (HAD) superfamily protein |
|  | 1610524_s_at | 13.66 | 16.56 | Heat shock cognate protein 70-1 |
|  | 1618355_at | 12.23 | 12.9 | Protein kinase family protein |
|  | 1620231_s_at | 11 | 13.31 | RING-H2 finger protein ATL4M |
|  | 1611374_at | 10.37 | 6.32 | Ubiquitin 11 |
|  | 1606705_at | 10.04 | 9.61 | Chaperone DnaJ-domain superfamily protein |
|  | 1616357_at | 9.15 | 13.31 | Protein kinase superfamily protein |
|  | 1608429_at | 9.02 | 24.26 | BCL-2-associated athanogene 5 |
|  | 1617053_at | 8.81 | 7 | Chloroplast thylakoidal processing peptidase |
|  | 1619542_at | 8.29 | 28.61 | Receptor-like protein kinase 1 |
|  | 1613630_s_at | 8.24 | 9.24 | Heat shock cognate protein 70-1 |
|  | 1609554_at | 8.14 | 58.36 | Heat shock protein 18.2 |
|  | 1609949_at | 7.67 | 8.27 | Heat shock cognate protein 70-1 |
|  | 1620823_at | 7.35 | 3.21 | Protein kinase superfamily protein |
|  | 1621565_s_at | 7.12 | 3.52 | CHY-type/CTCHY-type/RING-type Zinc finger protein |
|  | 1620348_at | 6.86 | 31.53 | Mitochondrion-localized small heat shock protein 23.6 |
|  | 1615895_at | 6.8 | 4.52 | Serine/threonine kinase |
|  | 1618804_at | 6.49 | 10.64 | RING/U-box superfamily protein |
|  | 1609252_at | 5.92 | 4.92 | Ubiquitin conjugating enzyme |
|  | 1609576_at | 5.91 | 5 | Protein phosphatase 2C family protein |
|  | 1614126_a_at | 5.8 | 5.71 | Cysteine proteinases superfamily protein |
|  | 1607899_at | 5.36 | 7.15 | Chaperone DnaJ-domain superfamily protein |
|  | 1622773_at | 5.36 | 5.55 | RING-H2 subgroup RHE protein |
|  | 1614205_at | 5.35 | 6.06 | RING/FYVE/PHD zinc finger superfamily protein |
|  | 1613463_a_at | 5.27 | 6.36 | Chaperone DnaJ-domain superfamily protein |
|  | 1616223_at | 5.19 | 4.26 | RING/U-box superfamily protein |
|  | 1620812_at | 5.08 | 5.74 | Chloroplast thylakoidal processing peptidase |
|  | 1608041_at | 4.99 | 3.67 | Signal recognition particle receptor alpha subunit family protein |
|  | 1615192_x_at | 4.8 | 4.06 | Ubiquitin 11 |
|  | 1616813_at | 4.75 | 6.59 | Chaperone DnaJ-domain superfamily protein |
|  | 1609766_at | 4.73 | 5.25 | Aspartic proteinase nepenthesin II |
|  | 1611662_at | 4.69 | 7.53 | P-loop containing nucleoside triphosphate hydrolases superfamily protein |
|  | 1609665_a_at | 4.69 | 3.34 | Protein phosphatase 2C family protein |
|  | 1609190_at | 4.49 | 4.35 | RING-H2 subgroup RHE protein |
|  | 1621296_at | 4.42 | 3.41 | Chloroplast thylakoidal processing peptidase-like protein |
|  | 1613079_at | 4.32 | 6.21 | Phosphotyrosine protein phosphatases superfamily protein |
|  | 1611674_at | 4.31 | 4.07 | Zinc finge |
|  | 1615076_at | 4.11 | 3.99 | Protein phosphatase 2C family protein |
|  | 1611192_at | 4.09 | 7.6 | 17.6 kDa class II heat shock protein |
|  | 1614391_at | 4.05 | 2.52 | Protein phosphatase 2C family protein |
|  | 1619976_at | 4.03 | 3.5 | RING/U-box superfamily protein |
|  | 1619061_at | 3.99 | 5 | Protein phosphatase 2A regulatory B subunit family protein |
|  | 1622184_at | 3.84 | 2.48 | RING/U-box superfamily protein |
|  | 1622807_at | 3.75 | 2.27 | RING/U-box superfamily protein |
|  | 1618862_at | 3.5 | 2.23 | E3 ubiquitin ligase PUB14 |
|  | 1621388_at | 3.4 | 2.52 | RING/U-box superfamily protein |
|  | 1606734_at | 3.38 | 3.63 | Serine/threonine/tyrosine kinase |
|  | 1612175_s_at | 3.33 | 3.22 | Chaperone DnaJ-domain superfamily protein |
|  | 1607392_at | 3.26 | 3.94 | Heat shock factor 3 |
|  | 1613124_s_at | 3.22 | 2.3 | Polyubiquitin (UBQ10) |
|  | 1619236_at | 3.18 | 4.41 | Mitochondrial HSO70 2 |
|  | 1622107_at | 3.16 | 2.75 | Protein kinase superfamily protein |
|  | 1615565_x_at | 3.16 | 2.63 | Ubiquitin 11 |
|  | 1617313_a_at | 3.05 | 2.05 | Serine/threonine-protein phosphatase PP1 |
|  | 1618638_at | 3.05 | 5.2 | Protein kinase superfamily protein |
|  | 1609336_s_at | 3.04 | 4.13 | Serine/threonine kinase GDBrPK |
|  | 1619931_s_at | 2.99 | 4.83 | Heat shock protein 90 |
|  | 1619202_s_at | 2.91 | 8.5 | Heat shock protein 70 (Hsp 70) family protein |
|  | 1607002_at | 2.89 | 11.28 | Heat shock protein 70 (Hsp 70) family protein |
|  | 1619528_s_at | 2.88 | 14.54 | Heat shock protein 70 (Hsp 70) family protein |
|  | 1618619_at | 2.87 | 4.86 | Heat shock protein 81-2 |
|  | 1609088_at | 2.84 | 3.43 | Heat shock factor 3 |
|  | 1614730_at | 2.84 | 4.19 | Non-ATPase subunit 9 |
|  | 1606558_at | 2.83 | 2.83 | RING/U-box superfamily protein |
|  | 1611080_at | 2.82 | 5.38 | Chaperone DnaJ-domain superfamily protein |
|  | 1608500_at | 2.81 | 3.73 | Protein kinase family protein |
|  | 1609460_at | 2.74 | 3.26 | Serine/threonine kinase GDBrPK |
|  | 1614508_at | 2.73 | 7.39 | Peptidase S24/S26A/S26B/S26C family protein |
|  | 1613678_at | 2.66 | 2.16 | Phosphatidic acid phosphatase (PAP2) family protein |
|  | 1608762_at | 2.58 | 4.91 | Heat shock factor |
|  | 1618009_at | 2.56 | 3.84 | Heat shock protein 81.4 |
|  | 1619684_at | 2.55 | 2.56 | Aspartyl protease family protein |
|  | 1608052_s_at | 2.54 | 4.52 | Similar to heat shock protein 90 |
|  | 1610570_at | 2.54 | 2.45 | Calcium-dependent protein kinase 18 |
|  | 1612385_at | 2.53 | 4.98 | Heat shock protein 18.2 |
|  | 1606447_at | 2.52 | 2.28 | Serine/threonine protein phosphatase |
|  | 1611726_at | 2.51 | 3.01 | Protein kinase superfamily protein |
|  | 1607382_at | 2.51 | 3.67 | Protein kinase superfamily protein |
|  | 1622257_at | 2.51 | 2.15 | Casein kinase 1-like protein 2 |
|  | 1614354_at | 2.49 | 4.12 | pfkB-like carbohydrate kinase family protein |
|  | 1619038_at | 2.49 | 4.7 | Uridine kinase-like 4 |
|  | 1620721_at | 2.46 | 2.06 | Ubiquitin protein ligase 5 |
|  | 1620385_s_at | 2.45 | 4.47 | Similar to ubiquitin fusion protein |
|  | 1612044_s_at | 2.44 | 3.37 | P-loop containing nucleoside triphosphate hydrolases superfamily protein |
|  | 1613325_at | 2.42 | 2.34 | Metacaspase 1 |
|  | 1614318_at | 2.42 | 3.94 | Xylem bark cysteine peptidase 3 |
|  | 1613560_at | 2.41 | 3.6 | Prolyl oligopeptidase family protein |
|  | 1618072_at | 2.4 | 3.85 | Dual-specificity phosphatase-like protein |
|  | 1614861_at | 2.38 | 2.19 | Aspartyl protease family protein |
|  | 1607207_at | 2.32 | 2.87 | Protein kinase superfamily protein |
|  | 1609392_s_at | 2.32 | 2.26 | Sumo conjugation enzyme 1 |
|  | 1621762_at | 2.28 | 2.54 | Ubiquitin-conjugating enzyme 13 |
|  | 1615682_s_at | 2.28 | 2.44 | Rotamase cyclophilin 5 73 |
|  | 1610033_s_at | 2.25 | 2.14 | Chaperonin-like RbcX protein |
|  | 1617431_at | 2.25 | 4.74 | Subtilisin-like protease |
|  | 1619965_at | 2.21 | 2.39 | RING/U-box superfamily protein |
|  | 1607595_at | 2.2 | 2.13 | Similar to serine/threonine protein phosphatase 1 |
|  | 1616979_s_at | 2.19 | 2.67 | Ubiquitin carrier protein |
|  | 1618567_at | 2.19 | 2.14 | Ubiquitin carrier protein |
|  | 1613526_at | 2.18 | 3.54 | Heat shock protein 81-3 |
|  | 1608448_at | 2.15 | 7.9 | Chaperone protein htpG family protein |
|  | 1610440_s_at | 2.11 | 3.21 | Pyruvate kinase family protein |
|  | 1620007_at | 2.09 | 3.02 | PAM domain (PCI/PINT associated module) protein |
|  | 1614382_at | 2.06 | 2.03 | P-loop containing nucleoside triphosphate hydrolases superfamily protein |
|  | 1620037_at | 2 | 2.61 | Heat shock factor 3 |
|  | 1617846_at | 2 | 2.01 | Ubiquitin carrier protein |
| Protein with binding function | 1610057_at | 18.23 | 24.5 | Poly(A)-binding protein C-terminal interacting protein 6 |
|  | 1614725_at | 13.58 | 11.87 | WRKY DNA-binding protein 70 |
|  | 1614505_s_at | 8.05 | 8.05 | F-box family protein |
|  | 1620207_at | 7.56 | 20.19 | Yippee family putative zinc-binding protein |
|  | 1610784_at | 6.25 | 8.72 | Acyl activating enzyme 5 |
|  | 1607718_at | 5.83 | 2.91 | Sequence-specific DNA binding transcription factors |
|  | 1619241_at | 5.67 | 5.48 | PLATZ transcription factor family protein |
|  | 1621329_at | 5.17 | 4.58 | Cold, circadian rhythm, and rna binding 2 |
|  | 1615828_at | 4.81 | 3.19 | Transducin/WD40 repeat-like superfamily protein |
|  | 1611502_at | 4.62 | 2.48 | Sequence-specific DNA binding transcription factors |
|  | 1606895_at | 4.22 | 6.98 | BTB/POZ domain-containing protein |
|  | 1622850_at | 4.21 | 5.63 | 2-oxoglutarate (2OG) and Fe(II)-dependent oxygenase superfamily protein |
|  | 1608488_at | 4.2 | 5.91 | AT-hook DNA-binding family protein |
|  | 1618075_at | 3.82 | 2.58 | Calcium-binding EF-hand family protein |
|  | 1612078_at | 3.38 | 3.21 | S-ribonuclease binding protein 1 |
|  | 1608464_at | 3.35 | 2.04 | Zinc finger protein-related |
|  | 1607270_at | 3.26 | 3.17 | Plantacyanin |
|  | 1619024_at | 3.09 | 3.61 | BTB-POZ and MATH domain 2 |
|  | 1617561_at | 3.04 | 4.21 | 2-oxoglutarate (2OG) and Fe(II)-dependent oxygenase superfamily protein |
|  | 1621554_s_at | 2.89 | 3.2 | Cold, circadian rhythm, and rna binding 2 |
|  | 1607637_at | 2.87 | 3.92 | Nucleic acid-binding, OB-fold-like protein |
|  | 1622835_at | 2.76 | 2.99 | BTB-POZ and MATH domain 2 |
|  | 1616828_s_at | 2.73 | 2.95 | Glycine-rich RNA-binding protein |
|  | 1620881_at | 2.68 | 2.98 | RNA-binding protein 47B |
|  | 1608317_at | 2.53 | 2.1 | NPR1-like protein 3 |
|  | 1621975_at | 2.53 | 4.41 | AT hook motif DNA-binding family protein |
|  | 1617424_at | 2.45 | 3.66 | Similarity to RNA binding protein |
|  | 1611553_s_at | 2.36 | 2.88 | Evolutionarily conserved C-terminal region 2 |
|  | 1619181_at | 2.3 | 3.67 | TUDOR-SN protein 2 |
|  | 1614397_at | 2.3 | 3.66 | Copper amine oxidase family protein |
|  | 1622047_at | 2.14 | 2.73 | AMP-dependent synthetase and ligase family protein |
|  | 1620524_at | 2.09 | 2.52 | RING/U-box superfamily protein |
| Protein activity regulation | 1609395_at | 14.95 | 12.19 | Cystatin-like protein |
|  | 1616279_at | 14.35 | 16.42 | RCD one 2 |
|  | 1622203_at | 8.25 | 232.89 | Kunitz family trypsin and protease inhibitor protein |
|  | 1608916_at | 6.09 | 6.82 | Similar to RCD one 2 |
|  | 1615360_at | 5.78 | 5.27 | Cystatin/monellin superfamily protein |
|  | 1611666_s_at | 4.74 | 11.11 | Serine protease inhibitor, potato inhibitor I-type family protein |
|  | 1606758_at | 3.12 | 5.03 | Cystatin B |
| Transport regulation | 1613922_s_at | 71.66 | 20.76 | HCO3- transporter family |
|  | 1611820_at | 65.46 | 92.33 | DC1 domain-containing protein |
|  | 1622455_at | 62.8 | 32.89 | Putative nitrate transporter NRT1-3 |
|  | 1610800_at | 54.59 | 77.45 | Transmembrane amino acid transporter family protein |
|  | 1620065_at | 51.47 | 84.56 | Sulfate transporter |
|  | 1611957_s_at | 45.6 | 10.89 | Amino acid transporter 1 |
|  | 1615540_s_at | 44.73 | 51.15 | Peptide transporter 3 |
|  | 1610363_at | 41.86 | 47.15 | Multidrug resistance protein 17 |
|  | 1618422_s_at | 36.34 | 21.77 | Heavy metal transport/detoxification superfamily protein |
|  | 1612194_at | 33.25 | 7.99 | Amino acid transporter 1 |
|  | 1617470_s_at | 29.93 | 22.15 | Lysine histidine transporter 1 |
|  | 1621817_at | 20.92 | 32.18 | Protein disulfide isomerase (PDI)-like protein 2 |
|  | 1610741_at | 20.64 | 16.42 | Heavy metal transport/detoxification superfamily protein |
|  | 1611925_at | 19.19 | 10.24 | RING/U-box superfamily protein |
|  | 1621261_at | 17.6 | 9.38 | Exocyst subunit exo70 family protein H4 |
|  | 1607088_at | 15.8 | 15.5 | Mitochondrial substrate carrier family protein |
|  | 1613837_at | 15.61 | 10.06 | Transmembrane amino acid transporter family protein |
|  | 1613840_at | 12.47 | 10.75 | Pleiotropic drug resistance 12 |
|  | 1609917_at | 12.08 | 14.06 | Mitochondrial dicarboxylate carrier protein |
|  | 1621156_at | 11.7 | 13.31 | Mitochondrial phosphate transporter |
|  | 1619168_at | 11.59 | 11.89 | Uncoupling protein 5 |
|  | 1610394_at | 11.19 | 19.42 | Ferretin 1 |
|  | 1610839_at | 11.15 | 10.56 | Peroxisomal adenine nucleotide carrier 1 |
|  | 1621683_x_at | 10.75 | 8.53 | Heavy metal transport/detoxification superfamily protein |
|  | 1613603_at | 10.6 | 6.91 | Transmembrane amino acid transporter family protein |
|  | 1609986_at | 10.59 | 17.15 | Equilibrative nucleoside transporter 6 |
|  | 1609370_at | 10.09 | 10.27 | Transmembrane amino acid transporter family protein |
|  | 1622786_at | 9.81 | 9.77 | Soluble N-ethylmaleimide-sensitive factor adaptor protein 33 |
|  | 1609405_at | 9.36 | 8.68 | Mitochondrial substrate carrier family protein |
|  | 1616929_at | 9.19 | 7.13 | Multidrug resistance-associated protein 3 |
|  | 1615919_s_at | 9.07 | 4.86 | Itrate transmembrane transporters |
|  | 1622620_at | 8.09 | 4.19 | ATP-binding cassette A2 |
|  | 1615479_at | 8.07 | 8.04 | GOLD family protein |
|  | 1610949_s_at | 7.57 | 7.15 | Heavy metal transport/detoxification superfamily protein |
|  | 1611326_at | 7.51 | 15.52 | Sugar transporte |
|  | 1613114_at | 7.36 | 4.4 | Calcium ion binding / peptidase |
|  | 1608365_at | 7.23 | 8.77 | Syntaxin of plants 121 |
|  | 1618589_s_at | 6.56 | 3.75 | Actinorizal nodulin AgNOD-GHRP |
|  | 1618367_at | 6.37 | 6.46 | Phosphate transporter 3;1 |
|  | 1609260_at | 6.36 | 9.59 | Mitochondrial substrate carrier family protein |
|  | 1613997_at | 6.03 | 13.19 | Autoinhibited Ca2+-ATPase 11 |
|  | 1608605_at | 5.72 | 5.15 | Major facilitator superfamily protein |
|  | 1617662_at | 5.63 | 12.11 | Atypical CYS HIS rich thioredoxin 4 |
|  | 1621674_at | 5.61 | 4.12 | Small and basic intrinsic protein 2;1 |
|  | 1610502_at | 5.53 | 4.09 | Chloride channel D |
|  | 1607681_at | 5.47 | 3.99 | Phosphate transporter 1;4 |
|  | 1609534_at | 5.38 | 6.39 | Translocase of inner mitochondrial membrane 23 |
|  | 1611039_s_at | 5.33 | 5.05 | SNARE domain containing protein |
|  | 1608566_s_at | 5.27 | 5.29 | Heavy metal transport/detoxification superfamily protein |
|  | 1619017_at | 5.25 | 4.8 | Mitochondrial inner membrane protein |
|  | 1619820_s_at | 5.12 | 4.37 | Atypical CYS HIS rich thioredoxin 2 |
|  | 1619233_at | 5.1 | 3.91 | Cation efflux family protein |
|  | 1612675_at | 4.98 | 5.41 | ADP,ATP carrier protein, mitochondrial |
|  | 1610027_at | 4.83 | 12.77 | D-isomer specific 2-hydroxyacid dehydrogenase family protein |
|  | 1619984_at | 4.79 | 4.93 | Amino acid permease 3 |
|  | 1615110_at | 4.71 | 4.29 | Atypical CYS HIS rich thioredoxin 2 |
|  | 1608940_at | 4.68 | 4.23 | Proline transporter 1 |
|  | 1613896_at | 4.52 | 6.95 | Major facilitator superfamily protein |
|  | 1620683_at | 4.45 | 3.76 | Atypical CYS HIS rich thioredoxin 2 |
|  | 1622191_at | 4.44 | 5.47 | Sulfate transporter 1;3 |
|  | 1611892_s_at | 4.27 | 3.72 | Proline transporter 1 |
|  | 1621567_at | 4.22 | 4.23 | Translocase inner membrane subunit 17-2 |
|  | 1618013_at | 4.11 | 5.18 | Staurosporin and temperature sensitive 3-like A |
|  | 1619989_s_at | 3.85 | 4.17 | ADP,ATP carrier protein, mitochondrial |
|  | 1608666_s_at | 3.77 | 6.75 | Pyrimidine nucleotide sugar transmembrane transporter |
|  | 1608259_at | 3.76 | 4.21 | Zinc transporter |
|  | 1618016_at | 3.63 | 3.04 | Phosphate/phosphoenolpyruvate translocator protein-like |
|  | 1616901_at | 3.46 | 5.18 | Phosphate transporter 3;1 |
|  | 1618607_s_at | 3.44 | 4.67 | ZRT/IRT-like protein 2 |
|  | 1621072_at | 3.42 | 5.9 | Purine permease 1 |
|  | 1612288_at | 3.37 | 5.17 | YELLOW STRIPE like 1 |
|  | 1611889_at | 3.34 | 3.21 | Emp24/gp25L/p24 family/GOLD family protein |
|  | 1613916_at | 3.27 | 3.3 | Oligopeptide transporter 4 |
|  | 1615383_at | 3.21 | 3.27 | UDP-N-acetylglucosamine transporter |
|  | 1620313_at | 3.2 | 4.49 | Phosphate transporter 3;1 |
|  | 1613466_at | 3.2 | 3.24 | Cationic amino acid transporter 2 |
|  | 1620992_at | 3.12 | 3.01 | Heavy metal transport/detoxification superfamily protein |
|  | 1613679_at | 3.07 | 3.16 | SNARE domain containing protein |
|  | 1606833_at | 3.05 | 2.48 | Chloride channel D |
|  | 1608501_at | 3.03 | 2.37 | ATPase E1-E2 type family protein |
|  | 1620565_at | 2.95 | 5.05 | UDP-galactose transporter 3 |
|  | 1614548_at | 2.95 | 4.5 | Purine permease 1 |
|  | 1608643_at | 2.94 | 4.92 | Phosphate/phosphoenolpyruvate translocator |
|  | 1613261_at | 2.92 | 2.95 | Transmembrane amino acid transporter family protein |
|  | 1613578_s_at | 2.89 | 2.98 | NAD+ transporter 1 |
|  | 1615396_at | 2.88 | 7.59 | ABC transporter |
|  | 1621568_at | 2.84 | 3.21 | ENTH/ANTH/VHS superfamily protein |
|  | 1610712_s_at | 2.8 | 3.71 | Mitochondrial phosphate transporter |
|  | 1616603_at | 2.75 | 3.08 | Translocase inner membrane subunit 8 |
|  | 1617051_at | 2.73 | 2.05 | Cytochrome P450 |
|  | 1621648_at | 2.72 | 2.69 | Cellular retinaldehyde binding/alpha-tocopherol transport |
|  | 1620975_at | 2.69 | 2.9 | Vesicle transport protein |
|  | 1614613_at | 2.68 | 3.49 | Plant VAP homolog 12 |
|  | 1614376_s_at | 2.68 | 3.63 | Plant VAP homolog 12 |
|  | 1610358_at | 2.67 | 2.61 | Nucleotide-sugar transporter family protein |
|  | 1621624_s_at | 2.66 | 4.67 | Peroxisomal membrane 22 kDa (Mpv17/PMP22) family protein |
|  | 1609687_at | 2.61 | 2.41 | Epsin N-terminal homology (ENTH) domain-containing protein |
|  | 1621604_at | 2.56 | 2.62 | Target of Myb protein 1 |
|  | 1609266_at | 2.56 | 2.19 | Glutaredoxin family protein |
|  | 1619506_s_at | 2.55 | 3.59 | Translocon-associated protein (TRAP) |
|  | 1618058_at | 2.53 | 6.17 | Putative anthocyanin permease |
|  | 1618142_at | 2.52 | 3.44 | Translocon-associated protein (TRAP) |
|  | 1622085_at | 2.49 | 2.43 | Sec14p-like phosphatidylinositol transfer family protein |
|  | 1621192_at | 2.47 | 2.94 | Thioredoxin superfamily protein |
|  | 1609051_at | 2.46 | 2.32 | Sec14p-like phosphatidylinositol transfer family protein |
|  | 1606997_at | 2.44 | 2.54 | Endomembrane protein 70 protein family |
|  | 1620932_at | 2.44 | 2.7 | Syntaxin of plants 43 |
|  | 1617252_s_at | 2.4 | 2.68 | Target of Myb protein 1 |
|  | 1616827_at | 2.35 | 2.59 | Clathrin adaptor complexes medium subunit family protein |
|  | 1617829_at | 2.34 | 2.39 | Heavy metal transport/detoxification superfamily protein |
|  | 1619602_at | 2.27 | 2.14 | Uncoupling protein 5 |
|  | 1619908_at | 2.26 | 2.03 | Ca-dependent solute carrier-like protein |
|  | 1619996_at | 2.25 | 2.5 | NAD+ transporter 1 |
|  | 1615140_s_at | 2.24 | 2.05 | Putative amino acid transport protein AAP2 |
|  | 1619703_at | 2.23 | 2.49 | Putative aquaporin PIP1-1 |
|  | 1615722_s_at | 2.22 | 2.51 | Plasma membrane intrinsic protein 1;4 |
|  | 1613011_at | 2.21 | 2.66 | ABC transporter |
|  | 1621011_at | 2.17 | 2.83 | Similar to H(+)-transporting ATPase |
|  | 1607472_at | 2.16 | 2.32 | Dynamin-like protein 6 |
|  | 1613240_s_at | 2.15 | 2.21 | Aquaporin PIP1 |
|  | 1609432_at | 2.1 | 2.5 | Preprotein translocase Sec, Sec61-beta subunit protein |
|  | 1607075_at | 2.1 | 2.03 | Syntaxin of plants 81 |
|  | 1610079_at | 2.08 | 2.5 | HA2 \| H(+)-ATPase 2 |
|  | 1617849_at | 2.05 | 2.16 | Multidrug resistance-associated protein 4 |
|  | 1610955_s_at | 2 | 2.6 | Aquaporin TIP2;1 |
|  | 1610875_at | 2 | 2.7 | Similar to coatomer protein epsilon subunit family protein / COPE family protein |
| Signal transduction | 1619208_at | 20.59 | 16.88 | BON association protein 2 |
|  | 1606597_at | 18.54 | 9.61 | Leucine-rich receptor-like protein kinase family protein |
|  | 1612734_at | 16.62 | 16.13 | Receptor kinase-like protein |
|  | 1615687_at | 16.55 | 19.89 | Putative protein kinase |
|  | 1620074_at | 15.14 | 6.3 | Calmodulin-like 11 |
|  | 1613324_at | 14.15 | 16.43 | Leucine-rich repeat transmembrane protein kinase |
|  | 1606881_at | 13.29 | 13.19 | Mitogen-activated protein kinase 3 |
|  | 1615060_at | 13.27 | 5.19 | Choline kinase 1 |
|  | 1621542_at | 13.05 | 16.19 | XB3 ortholog 1 in Arabidopsis thaliana |
|  | 1608006_at | 12.76 | 13.11 | Response regulator |
|  | 1607656_at | 11.64 | 11.24 | MAP kinase-like |
|  | 1614595_at | 10.55 | 11.27 | Mitogen-activated protein kinase |
|  | 1610522_a_at | 10.12 | 9.8 | Putative leucine-rich repeat receptor kinase |
|  | 1608981_at | 10.02 | 7.51 | Alpha/beta-Hydrolases superfamily protein |
|  | 1621727_s_at | 9.56 | 7.08 | Calcium-dependent protein kinase 15 |
|  | 1613675_at | 9.37 | 15.18 | Leucine-rich repeat transmembrane protein kinase |
|  | 1616541_at | 9.06 | 9.26 | U-box domain-containing protein kinase family protein |
|  | 1606667_at | 8.66 | 8.37 | Receptor kinase-like protein |
|  | 1614529_at | 7.88 | 7.22 | C2 domain-containing protein-like |
|  | 1614799_at | 7.61 | 2.4 | Calmodulin-binding protein |
|  | 1615561_at | 7.55 | 7.14 | Protein kinase |
|  | 1620080_at | 7.45 | 4.26 | Receptor kinase 2 |
|  | 1613552_at | 6.46 | 4.45 | Phosphatidylinositol-speciwc phospholipase C4 |
|  | 1608533_at | 5.96 | 17.18 | RAB GTPase homolog A6B |
|  | 1616487_at | 5.41 | 6.29 | Calcium-binding EF-hand family protein |
|  | 1613365_at | 5.34 | 6.36 | RING domain ligase2 |
|  | 1617369_at | 5.19 | 3.73 | Phosphatidylinositol-speciwc phospholipase C4 |
|  | 1621974_at | 5.18 | 4.58 | Mitogen-activated protein kinase phosphatase 1 |
|  | 1609397_at | 5.08 | 4.78 | GTP cyclohydrolase II |
|  | 1606638_at | 4.98 | 5.17 | MAP kinase kinase 5 |
|  | 1608373_at | 4.92 | 4.52 | MAP kinase kinase 5 |
|  | 1608127_s_at | 4.89 | 4.81 | MAP kinase kinase 2 |
|  | 1618587_at | 4.83 | 6.22 | Regulator of gene silencing |
|  | 1613007_at | 4.82 | 4.91 | RING domain ligase2 |
|  | 1617943_at | 4.74 | 5.02 | Leucine-rich repeat transmembrane protein kinase |
|  | 1619634_at | 4.65 | 5.58 | Calcineurin B-like protein 1 |
|  | 1620931_at | 4.63 | 3.06 | Phospholipase C 2 |
|  | 1612132_s_at | 4.56 | 4.82 | Phytochrome-associated protein phosphatase type 2C |
|  | 1621772_at | 4.5 | 3.97 | Calcium-dependent protein kinase family protein |
|  | 1612735_at | 4.47 | 4.91 | S-domain-2 5 |
|  | 1612860_at | 4.46 | 5.4 | ADP-ribosylation factor C1 |
|  | 1616893_s_at | 4.42 | 3.02 | Calmodulin like 23 |
|  | 1612466_at | 4.41 | 3.08 | Calmodulin like 23 |
|  | 1608140_at | 4.35 | 3.05 | Response regulator 6 |
|  | 1618208_s_at | 4.28 | 4.24 | Leucine-rich repeat (LRR) family protein |
|  | 1618364_at | 4.25 | 4.17 | Calcium-dependent lipid-binding (CaLB domain) family protein |
|  | 1608587_at | 4.21 | 2.91 | Calcium-dependent protein kinase 28 |
|  | 1620324_at | 4.05 | 2.62 | Leucine-rich repeat receptor-like protein kinase family protein |
|  | 1617279_at | 4.05 | 3.24 | Calmodulin like 42 |
|  | 1614255_at | 4.04 | 3.09 | Serine/threonine kinase |
|  | 1616321_at | 3.97 | 2.65 | SAUR-like auxin-responsive protein family |
|  | 1614319_at | 3.72 | 5.37 | Lectin-like receptor kinase 7;3 |
|  | 1622764_at | 3.6 | 6.96 | Ras-related small GTP-binding family protein |
|  | 1608400_at | 3.59 | 3.42 | MAP kinase kinase 2 |
|  | 1612784_at | 3.59 | 4.27 | Cysteine-rich RLK (RECEPTOR-like protein kinase) 10 |
|  | 1609827_at | 3.51 | 2.42 | Calcium-binding EF hand family protein |
|  | 1610143_at | 3.41 | 3.9 | Protein kinase superfamily protein |
|  | 1621219_at | 3.37 | 2.23 | Protein phosphatase 2C family protein |
|  | 1617059_at | 3.37 | 2.06 | With no lysine (K) kinase 1 |
|  | 1607044_at | 3.28 | 4.82 | Ras-related protein RGP1 |
|  | 1619027_at | 3.27 | 3.81 | S-locus lectin protein kinase family protein |
|  | 1619077_at | 3.12 | 3.4 | Secretion-associated RAS super family 2 |
|  | 1609131_at | 3.11 | 2.91 | Protein kinase family protein with leucine-rich repeat domain |
|  | 1607367_at | 3.09 | 4 | PR5-like receptor kinase |
|  | 1606757_at | 3.03 | 3.07 | ARF GAP-like zinc finger-containing protein ZIGA3 |
|  | 1607469_at | 3.03 | 4.1 | Calcium-dependent lipid-binding (CaLB domain) family protein |
|  | 1614414_at | 2.99 | 2.2 | Calcium-dependent lipid-binding (CaLB domain) family protein |
|  | 1615909_s_at | 2.97 | 2.32 | With no lysine (K) kinase 4 |
|  | 1620615_at | 2.93 | 3.36 | Integrin-linked protein kinase family |
|  | 1610796_at | 2.93 | 2.47 | Mitogen-activated protein kinase kinase kinase 3 |
|  | 1614582_at | 2.91 | 3.2 | Calreticulin-3 precursor |
|  | 1613364_at | 2.9 | 3.25 | Phosphatidylinositol-4-phosphate 5-kinase family protein |
|  | 1613692_at | 2.86 | 2.06 | Calmodulin like 23 |
|  | 1619728_at | 2.78 | 3.67 | RAB GTPase homolog A4C |
|  | 1620562_at | 2.78 | 2.79 | Calmodulin-binding family protein |
|  | 1621910_a_at | 2.75 | 3.04 | ADP-ribosylation factor |
|  | 1607385_at | 2.74 | 2.01 | Receptor-like protein kinase-like protein |
|  | 1614767_at | 2.73 | 3.81 | Chitin elicitor receptor kinase 1 |
|  | 1611449_at | 2.59 | 2.2 | GTPase activating protein |
|  | 1622351_at | 2.58 | 2.2 | Calmodulin 7 |
|  | 1609774_s_at | 2.56 | 3.21 | RAB homolog 1 |
|  | 1617387_at | 2.51 | 2.06 | MAPK/ERK kinase kinase 1 |
|  | 1609428_at | 2.49 | 4.63 | Leucine-rich repeat protein kinase family protein |
|  | 1613576_s_at | 2.48 | 2.35 | Calmodulin 7 |
|  | 1618445_s_at | 2.46 | 3.28 | Protein kinase superfamily protein |
|  | 1611917_at | 2.44 | 12.51 | Calreticulin family protein |
|  | 1622220_at | 2.41 | 2.23 | pfkB-like carbohydrate kinase family protein |
|  | 1618421_at | 2.38 | 2.04 | N-acetyl-l-glutamate kinase |
|  | 1608239_at | 2.36 | 2.42 | ADP-ribosylation factor 3 |
|  | 1617987_at | 2.14 | 2.2 | Similar to ADP-ribosylation factor isoform 1 |
|  | 1606886_at | 2.13 | 2.75 | Plant calmodulin-binding protein-related |
|  | 1611918_s_at | 2.13 | 13.1 | Calnexin 1 |
|  | 1615495_at | 2.13 | 3.18 | Protein kinase family protein |
|  | 1617888_at | 2.11 | 2.86 | Nucleolar GTP-binding protein |
|  | 1621070_s_at | 2.1 | 3.33 | ENTH/VHS/GAT family protein |
|  | 1607409_at | 2.1 | 2.19 | Phosphatidylinositol 3,5-kinase-like |
|  | 1616759_at | 2.06 | 2.94 | Calcium-binding EF-hand family protein |
|  | 1611877_at | 2.05 | 3.08 | Elongation factor family protein |
|  | 1613847_s_at | 2.03 | 2.25 | Rhodopsin-like receptor |
| Cell rescue | 1611117_at | 273.16 | 356.95 | Disease resistance-responsive (dirigent-like protein) family protein |
|  | 1613461_s_at | 241.76 | 341.8 | Class IV chitinase |
|  | 1611710_at | 169.87 | 263.66 | Class IV chitinase |
|  | 1618568_s_at | 164.69 | 264.82 | MLP-like protein 423 |
|  | 1618663_s_at | 157.83 | 167.99 | Dirigent protein |
|  | 1610011_s_at | 120.55 | 169.04 | Pathogenesis-related protein 10.3 |
|  | 1617192_at | 107 | 68.16 | Class IV chitinase |
|  | 1613339_at | 100.77 | 196.45 | Polygalacturonase inhibiting protein 1 |
|  | 1621220_at | 74.57 | 77.5 | Blue-copper-binding protein |
|  | 1614141_at | 67.5 | 88.02 | Plant disease resistance polyprotein-like |
|  | 1618561_at | 63.04 | 70.25 | Short-chain type dehydrogenase/reductase |
|  | 1618326_x_at | 61.02 | 65.44 | Disease resistance-responsive (dirigent-like protein) family protein |
|  | 1614862_at | 56.63 | 150.29 | Short chain alcohol dehydrogenase |
|  | 1619710_at | 56.5 | 54.03 | Disease resistance-responsive (dirigent-like protein) family protein |
|  | 1613999_x_at | 55.75 | 78.07 | Chitinase A |
|  | 1621244_s_at | 54.41 | 59.92 | Short-chain alcohol dehydrogenase like protein |
|  | 1622652_at | 52.69 | 32.55 | Similarity to SRC2 protein |
|  | 1610704_at | 49.16 | 66.46 | Pathogenesis-related protein 10 |
|  | 1611643_at | 48.48 | 71.14 | Harpin inducing protein 1-like 9 |
|  | 1608864_s_at | 43.94 | 55.17 | Acidic endochitinase precursor |
|  | 1609330_at | 40.65 | 55.9 | Glutathione S-transferase TAU 25 |
|  | 1616064_at | 38.09 | 102.13 | Class IV chitinase |
|  | 1610013_s_at | 37.69 | 57.28 | Calcium-dependent lipid-binding (CaLB domain) family protein |
|  | 1613301_at | 32.37 | 28.3 | Aluminium induced protein with YGL and LRDR motifs |
|  | 1621239_at | 32.32 | 51.04 | Transmembrane receptors |
|  | 1618778_at | 30.31 | 48.76 | Avr9/Cf-9 rapidly elicited protein 75 |
|  | 1615552_at | 23.23 | 30.18 | Galactinol synthase 1 |
|  | 1614090_x_at | 19.12 | 34.61 | Disease resistance response protein-like |
|  | 1618835_s_at | 19.08 | 42.84 | Pathogenesis-related 4 |
|  | 1608260_at | 17.04 | 19.47 | Glutathione S-transferase tau 7 |
|  | 1609689_at | 16.95 | 29.75 | Disease resistance-responsive (dirigent-like protein) family protein |
|  | 1616933_at | 16.04 | 7.79 | Glutathione S-transferase |
|  | 1611890_at | 15.51 | 13.2 | Glutathione S-transferase TAU 8 |
|  | 1615301_at | 14.15 | 16.23 | Late embryogenesis abundant (LEA) Hydroxyproline-rich glycoprotein family |
|  | 1613871_at | 13.92 | 11.82 | Chitinase |
|  | 1621881_at | 12.78 | 21.43 | Aluminium induced protein with YGL and LRDR motifs |
|  | 1618110_s_at | 11.89 | 25.92 | Aluminium induced protein with YGL and LRDR motifs |
|  | 1608802_s_at | 11.67 | 18.33 | Glutathione S-transferase family protein |
|  | 1607408_at | 11.65 | 16.97 | NIMIN-1 protein |
|  | 1620043_at | 10.54 | 7.69 | Leucine-rich repeat protein kinase family protein |
|  | 1619115_s_at | 10.48 | 7.55 | Disease resistance-responsive (dirigent-like protein) family protein |
|  | 1621636_s_at | 10.24 | 6.73 | Glutathione S-transferase phi 8 |
|  | 1609321_at | 9.85 | 21.65 | Peroxidase superfamily protein |
|  | 1621829_at | 8.86 | 3.18 | EXORDIUM like 2 |
|  | 1622142_at | 8.77 | 7.51 | Receptor-like protein kinase 1 |
|  | 1613650_at | 8.42 | 4.94 | Shock protein SRC2 |
|  | 1607127_s_at | 8.14 | 10.01 | Pathogenesis-related family protein |
|  | 1614464_s_at | 7.81 | 8.67 | Pathogenesis-related protein 10 |
|  | 1614259_at | 7.26 | 2.95 | Putative stress related chitinase |
|  | 1606845_at | 6.83 | 18.4 | Phosphate-responsive 1 family protein |
|  | 1610433_at | 6.7 | 8.46 | Galactosyltransferase family protein |
|  | 1617752_at | 6.6 | 12.43 | Aspartyl protease family protein |
|  | 1618308_at | 6.51 | 6.74 | Diacylglycerol kinase 2 |
|  | 1616320_x_at | 6.3 | 6.89 | Dirigent protein |
|  | 1617736_a_at | 6.18 | 7.05 | Rubber elongation factor protein (REF) |
|  | 1609971_at | 5.89 | 4.51 | Similarity to SRC2 protein |
|  | 1616703_at | 5.65 | 5.72 | Thioredoxin superfamily protein |
|  | 1606987_at | 5.59 | 3.47 | Leucine Rich Repeat family protein |
|  | 1608995_at | 5.54 | 10.76 | Alkaline alpha galactosidase II |
|  | 1607557_at | 5.18 | 31.96 | Class IV chitinase |
|  | 1616735_at | 5.04 | 14.26 | Similar to UP\|Q7Y0S8_SOLTU (Q7Y0S8) Erg-1 |
|  | 1608263_a_at | 4.91 | 5.54 | Alcohol dehydrogenase 1 |
|  | 1617909_at | 4.91 | 4.51 | Disease resistance protein (TIR-NBS-LRR class) family |
|  | 1612535_s_at | 4.89 | 4.05 | Glutathione S-transferase family protein |
|  | 1606464_at | 4.68 | 2.84 | Thioredoxin superfamily protein |
|  | 1611058_at | 4.61 | 53.37 | Pathogenesis-related 1 protein) superfamily protein |
|  | 1618545_a_at | 4.54 | 6.88 | Stress-inducible protein |
|  | 1617430_s_at | 4.5 | 7.05 | Basic chitinase |
|  | 1610056_s_at | 4.35 | 3.07 | Serine-rich protein-related |
|  | 1621319_s_at | 4.24 | 17 | Class IV chitinase |
|  | 1608868_at | 4.21 | 3.74 | Receptor lectin kinase |
|  | 1616495_at | 4.2 | 8.14 | Glutathione S-transferase PHI 9 |
|  | 1612845_at | 4.15 | 3.34 | Serine-rich protein-related |
|  | 1613154_at | 4.07 | 5.97 | Stress-inducible protein |
|  | 1617358_s_at | 3.99 | 6.03 | Disease resistance protein (CC-NBS-LRR class) family |
|  | 1614741_at | 3.95 | 2.93 | Diacylglycerol kinase 5 |
|  | 1616706_at | 3.91 | 5.41 | Heat shock protein 70 (HSP70)-interacting protein, |
|  | 1614078_at | 3.87 | 3.4 | HSP20-like chaperones superfamily protein |
|  | 1607390_x_at | 3.79 | 40.64 | CAP (Cysteine-rich secretory proteins, Antigen 5, and Pathogenesis-related 1 protein) superfamily protein |
|  | 1609188_at | 3.78 | 3.16 | Calcium-binding EF-hand family protein |
|  | 1606625_at | 3.76 | 2.61 | Class IV chitinase |
|  | 1613160_s_at | 3.67 | 3.07 | HSP20-like chaperones superfamily protein |
|  | 1621681_at | 3.64 | 3.25 | Disease resistance protein (TIR-NBS-LRR class) family |
|  | 1612826_at | 3.59 | 39.36 | CAP (Cysteine-rich secretory protein |
|  | 1620505_at | 3.5 | 4.67 | Chitinase class I |
|  | 1609893_at | 3.45 | 8.96 | GAST1 protein homolog 1 |
|  | 1618650_a_at | 3.43 | 2.14 | VIER F-box proteine 3 |
|  | 1615967_at | 3.41 | 8.64 | Peroxidase 35 precursor |
|  | 1611876_s_at | 3.33 | 3.13 | Acidic endochitinase precursor |
|  | 1608819_at | 3.21 | 2.69 | E heterogeneous nuclear ribonucleoprotein |
|  | 1610054_at | 3.19 | 2.61 | Disease resistance protein (TIR-NBS-LRR class) family |
|  | 1618491_s_at | 3.13 | 6.85 | Alpha/beta-Hydrolases superfamily protein |
|  | 1618775_at | 3.06 | 3.1 | Pathogenesis-related family protein |
|  | 1619475_a_at | 2.88 | 2.38 | Wound-induced protein |
|  | 1620130_at | 2.87 | 3.45 | Electron carriers |
|  | 1616947_at | 2.85 | 2.06 | Probable disease resistance protein At5g66900 |
|  | 1616081_at | 2.73 | 4.05 | Galactosyltransferase family protein |
|  | 1609922_s_at | 2.69 | 2.36 | Similarity to protein ref:NP_195581.1 (A.thaliana) |
|  | 1613994_at | 2.47 | 2.37 | Plant disease resistance polyprotein-like |
|  | 1610243_at | 2.47 | 3.13 | Glutathione S-transferase tau 7 |
|  | 1611490_at | 2.41 | 2.18 | Pathogenesis-related gene 1 |
|  | 1618856_at | 2.34 | 21.84 | Aluminium induced protein with YGL and LRDR motifs |
|  | 1619873_at | 2.21 | 4.65 | Zinc-binding alcohol dehydrogenase family protein |
|  | 1613816_x_at | 2.18 | 2.01 | Pathogenesis related protein 1 precursor |
|  | 1608262_at | 2.15 | 3.66 | Class I extracellular chitinase |
| Interaction with cellular environment | 1610316_at | 6.81 | 7.87 | Copper transporter 5 |
|  | 1614760_at | 4.95 | 5.07 | Cyclin-like F-box; Galactose oxidase |
|  | 1614018_at | 3.91 | 4 | Gigantea protein (GI) |
|  | 1607113_at | 2.58 | 2.53 | Copper transporter 5 |
| Plant / fungal specific systemic sensing and response | 1615458_at | 274.73 | 313.39 | Similarity to protein ref:NP_174140.1 (A.thaliana) |
|  | 1620276_at | 86.63 | 76.87 | S-adenosyl-L-methionine-dependent methyltransferases superfamily protein |
|  | 1616695_s_at | 74.15 | 107.87 | Thaumatin |
|  | 1610623_s_at | 46.59 | 25.49 | Auxin-responsive family protein |
|  | 1620390_s_at | 37.73 | 58.13 | Thaumatin-like protein |
|  | 1617510_s_at | 30.13 | 20.99 | Plant basic secretory protein (BSP) family protein |
|  | 1622147_at | 29.02 | 32.44 | Ethylene-forming enzyme |
|  | 1612552_at | 27.09 | 31.62 | S-adenosyl-L-methionine-dependent methyltransferases superfamily protein |
|  | 1618108_at | 21.61 | 13.77 | Plant basic secretory protein (BSP) family protein |
|  | 1616698_at | 18.89 | 18.55 | Ethylene-forming enzyme |
|  | 1606794_at | 15.09 | 21.84 | Thaumatin-like protein |
|  | 1622360_at | 14.83 | 18.43 | Nitrilase 4 |
|  | 1621371_at | 14.81 | 8.21 | Disease resistance-responsive (dirigent-like protein) family protein |
|  | 1610880_s_at | 14.4 | 6.36 | Indole-3-acetic acid-amido synthetase |
|  | 1609995_s_at | 14.14 | 14.46 | Ethylene-forming enzym |
|  | 1615090_at | 12.16 | 32.55 | Plant basic secretory protein (BSP) family protein |
|  | 1620071_at | 11.8 | 7.71 | CXE carboxylesterase |
|  | 1613180_at | 10.99 | 8.71 | Thaumatin |
|  | 1622745_at | 10.66 | 31.17 | Quinone reductase family protein |
|  | 1622824_at | 10.47 | 13.54 | Shaggy-like protein kinase 32 |
|  | 1618503_at | 8.67 | 2.79 | Gibberellin 2-oxidase 1 |
|  | 1620662_at | 6.39 | 3.67 | Auxin-responsive GH3 family protein |
|  | 1608502_at | 6.34 | 4.83 | Oxophytodienoate-reductase 3 |
|  | 1608330_at | 6.32 | 9.41 | SNF1-related protein kinase regulatory subunit gamma 1 |
|  | 1614975_at | 5.51 | 5.47 | Wound induced protein |
|  | 1610213_at | 5.49 | 4.76 | Somatic embryogenesis receptor-like kinase 1 |
|  | 1619261_s_at | 5.31 | 3.69 | HVA22 homologue A |
|  | 1609591_at | 5.27 | 8.43 | SAUR-like auxin-responsive protein family |
|  | 1610989_at | 5.1 | 4.98 | Glutathione S-transferase GST 13 |
|  | 1616423_at | 4.96 | 5.73 | Gibberellin 2-oxidase 6 |
|  | 1619610_at | 4.48 | 2.39 | Peptidase M20/M25/M40 family protein |
|  | 1610591_at | 4.25 | 3.2 | Jasmonic acid-amino acid-conjugating enzyme |
|  | 1617791_s_at | 4.23 | 2.24 | Dehydration-induced protein (ERD15) |
|  | 1606557_at | 4.22 | 4.47 | Bri1-associated receptor kinase |
|  | 1607503_s_at | 3.93 | 5.25 | Dormancy-associated protein-like |
|  | 1619716_at | 3.93 | 2.07 | Aldehyde dehydrogenase |
|  | 1622239_at | 3.6 | 3.01 | Thaumatin-like protein TLP8 |
|  | 1607225_at | 3.38 | 6.13 | Pathogenesis-related thaumatin superfamily protein |
|  | 1617572_at | 3.24 | 2.08 | Brassinosteroid-responsive RING-H2 |
|  | 1609419_at | 2.89 | 2.09 | HVA22 homologue A |
|  | 1622868_at | 2.87 | 3.55 | Wound induced protein-like |
|  | 1613811_a_at | 2.34 | 3.45 | 3-ketoacyl-CoA synthase 3 |
| Transposable elements | 1621369_at | 2.5 | 2.65 | SNF7 family protein |
|  | 1616404_at | 2.41 | 4.6 | Mitochondrial ATP synthase subunit G protein |
|  | 1609445_at | 2.33 | 2.3 | SNF7 family protein |
| Cell fate | 1612224_s_at | 48.26 | 45.66 | Putative phytosulfokine peptide precursor |
|  | 1620438_at | 7.59 | 36.36 | Expansin-like B1 |
|  | 1620443_s_at | 6.18 | 6.16 | Phytosulfokine 4 precursor |
|  | 1622456_at | 4.49 | 4.39 | Phytosulfokine 4 precursor |
|  | 1615995_at | 4.15 | 2.01 | Xyloglucan endotransglucosylase/hydrolase 24 |
|  | 1615809_at | 4.09 | 6.33 | Xyloglucan endotransglucosylase/hydrolase 30 |
|  | 1618150_at | 3.86 | 3.71 | Xyloglucan endotransglucosylase/hydrolase family protein |
|  | 1609804_at | 3.5 | 3.82 | Phytosulfokine 4 precursor |
|  | 1617306_at | 2.13 | 2.56 | SNF7 family protein |
|  | 1612697_s_at | 2.02 | 2.65 | Defender against death (DAD family) protein |
| Development | 1614607_at | 6.6 | 6.41 | Senescence-associated protein |
|  | 1615747_at | 4.07 | 4.06 | Enoyl-CoA hydratase/isomerase family |
|  | 1611620_a_at | 3.11 | 3.34 | Zinc finger (C3HC4-type RING finger) protein-like |
| Biogenesis of cellular component | 1616045_a_at | 11.29 | 30.48 | Proline-rich protein 2 |
|  | 1609487_at | 10.95 | 13.68 | Reversibly glycosylated polypeptide 4 |
|  | 1622152_at | 9.6 | 24.23 | RHO guanyl-nucleotide exchange factor 11 |
|  | 1622234_s_at | 9.03 | 9.68 | Reversibly glycosylated polypeptide 3 |
|  | 1609820_at | 8.69 | 16.22 | Similar to UniRef100_A7PNT8 |
|  | 1610563_at | 8.12 | 10.51 | Putative myosin heavy chain |
|  | 1610961_s_at | 6.82 | 10.35 | Dynein light chain, putative |
|  | 1617890_s_at | 6.37 | 10.75 | RHO guanyl-nucleotide exchange factor 11 |
|  | 1606478_at | 4.76 | 6.88 | Putative myosin heavy chain |
|  | 1620186_at | 4.01 | 2.42 | Arabinogalactan-protein precursor |
|  | 1619906_at | 3.59 | 3.92 | Pherophorin-C1 protein precursor |
|  | 1606756_at | 2.55 | 3.36 | Reversibly glycosylated polypeptide 1 |
|  | 1616561_at | 2.36 | 3.05 | Reversibly glycosylated polypeptide 3 |
|  | 1606588_s_at | 2.28 | 17.76 | Tubulin alpha-6 chain |
|  | 1621384_at | 2.23 | 4.53 | Proline rich protein 2 |
|  | 1615811_a_at | 2.1 | 2.08 | P-loop containing nucleoside triphosphate hydrolases superfamily protein |
|  | 1609447_at | 2.06 | 15.09 | Tubulin alpha-6 chain, putative |
