# Supplementary material for: Transcriptomic Analysis of Grape (Vitis vinifera L.) Leaves after Exposure to Ultraviolet C Irradiation
Source: PLoS One. 2014 Dec 2;9(12):e113772. doi: 10.1371/journal.pone.0113772 (PMC4252036; doi:10.1371/journal.pone.0113772)
Supplement: Additional file S4 — Probe sets commonly down-regulated at 6 and 12 h after exposure to UV-C irradiation. (DOCX) [file pone.0113772.s004.docx]

**Additional file S4** Genes down-regulated commonly at 6 and 12 h after UV-C treatment

|  |  | Fold change | |  |
| --- | --- | --- | --- | --- |
| Category | Probe set ID | 6 h | 12 h | Gene name description |
| Metabolism | 1622292_at | 0.02 | 0.02 | Pectin lyase-like superfamily protein |
|  | 1622891_at | 0.04 | 0.04 | Hydrolase |
|  | 1620140_at | 0.04 | 0.04 | Probable polygalacturonase |
|  | 1617693_at | 0.05 | 0.02 | Omega-6 fatty acid desaturase |
|  | 1614485_at | 0.05 | 0.08 | Putative anthranilate N-hydroxycinnamoyl/benzoyltransferase |
|  | 1622535_at | 0.05 | 0.05 | Plant invertase/pectin methylesterase inhibitor superfamily protein |
|  | 1618657_at | 0.05 | 0.05 | Probable polygalacturonase |
|  | 1616158_at | 0.05 | 0.04 | Pectate lyase |
|  | 1609552_at | 0.06 | 0.05 | Cytochrome P450, family 94, subfamily D, polypeptide 2 |
|  | 1613152_at | 0.07 | 0.05 | HXXXD-type acyl-transferase family protein |
|  | 1610511_at | 0.07 | 0.18 | Cytochrome P450, family 71, subfamily B, polypeptide 2 |
|  | 1606617_at | 0.07 | 0.07 | 2Fe-2S ferredoxin-like superfamily protein |
|  | 1607760_at | 0.07 | 0.06 | Flavonoid 3',5'-hydroxylase |
|  | 1618551_at | 0.07 | 0.05 | Flavonol synthase |
|  | 1606998_at | 0.07 | 0.1 | Pectate lyase |
|  | 1618297_s_at | 0.07 | 0.1 | Pectate lyase |
|  | 1618576_at | 0.07 | 0.06 | Pectin lyase-like superfamily protein |
|  | 1612003_at | 0.08 | 0.06 | Adenosylmethionine decarboxylase family protein |
|  | 1608313_at | 0.08 | 0.05 | Beta-D-xylosidase 4 |
|  | 1615198_at | 0.09 | 0.05 | Alpha-xylosidase 1 |
|  | 1607253_s_at | 0.09 | 0.07 | Thiazole biosynthetic enzyme, chloroplast (ARA6) (THI1) (THI4) |
|  | 1611847_at | 0.09 | 0.09 | Flavonoid 3',5'-hydroxylase |
|  | 1615481_at | 0.09 | 0.1 | Cytochrome B5 isoform D |
|  | 1609765_s_at | 0.09 | 0.11 | Leucoanthocyanidin dioxygenase |
|  | 1617019_at | 0.09 | 0.09 | Chalcone synthase |
|  | 1614994_at | 0.1 | 0.05 | GDSL-like Lipase/Acylhydrolase superfamily protein |
|  | 1620618_at | 0.1 | 0.05 | GDSL-like Lipase/Acylhydrolase superfamily protein |
|  | 1608882_at | 0.1 | 0.08 | Chlorophyllase 2 |
|  | 1608756_at | 0.1 | 0.04 | Pectin lyase-like superfamily protein |
|  | 1619578_at | 0.11 | 0.07 | Cytochrome P450, family 72, subfamily A, polypeptide 10 |
|  | 1609526_at | 0.11 | 0.05 | Long-chain fatty alcohol |
|  | 1609520_at | 0.11 | 0.12 | Mannan endo-1,4-beta-mannosidase |
|  | 1607307_at | 0.11 | 0.07 | Glucosyltransferase-11 |
|  | 1620751_at | 0.11 | 0.07 | Phytoene synthase |
|  | 1607732_at | 0.11 | 0.09 | Chalcone synthase |
|  | 1611095_a_at | 0.11 | 0.2 | Similar to UP\|Q9SI74_ARATH (Q9SI74) F23N19.12 |
|  | 1618849_at | 0.12 | 0.07 | Putative polygalacturonase |
|  | 1610602_a_at | 0.12 | 0.07 | Phytoene synthase, chloroplast precursor |
|  | 1615128_at | 0.12 | 0.13 | Putative glucosyltransferase |
|  | 1617699_at | 0.13 | 0.09 | Trehalose-6-phosphate phosphatase-like protein |
|  | 1606763_at | 0.13 | 0.11 | Glycosyl hydrolase superfamily protein |
|  | 1619522_at | 0.13 | 0.09 | Beta-galactosidase |
|  | 1614674_at | 0.13 | 0.05 | Sucrose-phosphate synthase 1 |
|  | 1606454_at | 0.13 | 0.08 | Phytoene synthase |
|  | 1619986_s_at | 0.13 | 0.17 | UDP-glucosyl transferase 88A1 |
|  | 1607739_at | 0.13 | 0.19 | Flavanone 3-hydroxylase |
|  | 1614175_at | 0.14 | 0.09 | Thiazole biosynthetic enzyme, Chloroplast (ARA6) (THI1) (THI4) |
|  | 1609268_at | 0.14 | 0.05 | Mannose-1-phosphate guanylyltransferase (GDP)s |
|  | 1618112_at | 0.14 | 0.14 | UDP-Glycosyltransferase superfamily protein |
|  | 1610415_at | 0.15 | 0.12 | Cytochrome P450, family 76, subfamily C, polypeptide 1 |
|  | 1612710_at | 0.15 | 0.04 | Putative acid phosphatase |
|  | 1612763_at | 0.15 | 0.12 | Putative ripening-related protein |
|  | 1614183_at | 0.16 | 0.07 | S-adenosyl-L-methionine-dependent methyltransferases superfamily protein |
|  | 1613538_at | 0.16 | 0.13 | Glycerol-3-phosphate acyltransferase 8 |
|  | 1612479_at | 0.16 | 0.08 | Glycerol-3-phosphate acyltransferase 4 |
|  | 1608048_at | 0.16 | 0.14 | NAD(P)-binding rossmann-fold superfamily protein |
|  | 1616093_at | 0.16 | 0.11 | Similar to pectin methylesterase |
|  | 1613330_at | 0.16 | 0.1 | Pectin lyase-like superfamily protein |
|  | 1616862_at | 0.17 | 0.18 | Cytochrome P450, family 706, subfamily A, polypeptide 5 |
|  | 1607407_at | 0.17 | 0.15 | Similar to nitrate reductase |
|  | 1610324_a_at | 0.17 | 0.1 | Glycosyl hydrolase superfamily protein |
|  | 1606646_at | 0.17 | 0.1 | Cellulose synthase family protein |
|  | 1616475_at | 0.17 | 0.08 | UDP-Glycosyltransferase superfamily protein |
|  | 1616275_at | 0.17 | 0.16 | Chlorophyllase 2 |
|  | 1622770_at | 0.17 | 0.12 | Glycosyl hydrolase 9C2 |
|  | 1615634_at | 0.18 | 0.21 | UDP-glucosyl transferase 88A1 |
|  | 1611081_a_at | 0.18 | 0.09 | Putative polygalacturonase |
|  | 1622464_at | 0.18 | 0.13 | Thiazole biosynthetic enzyme, chloroplast (ARA6) (THI1) (THI4) |
|  | 1615341_at | 0.19 | 0.12 | Putative ripening-related P-450 enzyme |
|  | 1617205_at | 0.19 | 0.14 | Tetrahydrodipicolinate N-succinyltransferase |
|  | 1612560_at | 0.19 | 0.09 | SKU5 similar 5 |
|  | 1618741_at | 0.19 | 0.09 | Salt tolerance homologue |
|  | 1618021_at | 0.19 | 0.09 | UDP-glucose glucosyltransferase |
|  | 1611389_at | 0.19 | 0.12 | Isoflavone reductase-like protein 6 |
|  | 1611597_at | 0.2 | 0.19 | Accelerated cell death 2 (ACD2) |
|  | 1615818_at | 0.2 | 0.21 | Adenosylmethionine decarboxylase family protein |
|  | 1622130_at | 0.2 | 0.1 | Sterol 4-alpha-methyl-oxidase 2-1 |
|  | 1613768_at | 0.2 | 0.18 | Uroporphyrinogen decarboxylase |
|  | 1610325_at | 0.21 | 0.18 | Cytochrome P450, family 71, subfamily A, polypeptide 25 |
|  | 1615986_at | 0.21 | 0.15 | Galacturonosyltransferase 6 |
|  | 1615159_s_at | 0.21 | 0.17 | Similar to nitrate reductase |
|  | 1610341_at | 0.21 | 0.33 | Purple acid phosphatase 12 |
|  | 1607272_at | 0.21 | 0.17 | Cellulose synthase-like A02 |
|  | 1616528_s_at | 0.21 | 0.11 | Lipolytic enzyme, G-D-S-L |
|  | 1618054_at | 0.21 | 0.24 | PATATIN-like protein 9 |
|  | 1617249_at | 0.21 | 0.09 | Acyl carrier protein |
|  | 1606449_at | 0.21 | 0.11 | Deoxyxylulose-5-phosphate synthase |
|  | 1622049_at | 0.21 | 0.12 | Similarity to At3g57030 |
|  | 1620424_at | 0.21 | 0.18 | Putative chalcone isomerase 4 |
|  | 1614670_at | 0.22 | 0.31 | Hydroxysteroid dehydrogenase 1 |
|  | 1607848_at | 0.22 | 0.28 | Phosphate starvation-induced gene 2 |
|  | 1609652_s_at | 0.22 | 0.14 | Cellulose synthase-like A02 |
|  | 1614465_at | 0.22 | 0.19 | Pentatricopeptide repeat (PPR-like) superfamily protein |
|  | 1614467_at | 0.22 | 0.18 | Thioesterase superfamily protein |
|  | 1621418_at | 0.22 | 0.08 | UDP-Glycosyltransferase superfamily protein |
|  | 1612672_at | 0.22 | 0.21 | Pectinesterase |
|  | 1622687_at | 0.23 | 0.12 | Cytochrome P450, family 78, subfamily A, polypeptide 8 |
|  | 1608621_at | 0.23 | 0.15 | UDP-Glycosyltransferase superfamily protein |
|  | 1610379_at | 0.23 | 0.12 | S-adenosyl-L-methionine-dependent methyltransferases superfamily protein |
|  | 1616007_at | 0.23 | 0.15 | Nucleotide-diphospho-sugar transferases superfamily protein |
|  | 1612311_at | 0.23 | 0.11 | Putative thiamin biosynthesis protein |
|  | 1618991_s_at | 0.23 | 0.22 | Isoflavone reductase-like protein 6 |
|  | 1606663_at | 0.23 | 0.17 | Chalcone synthase |
|  | 1609184_at | 0.24 | 0.05 | Cytochrome P450, family 72, subfamily A, polypeptide 14 |
|  | 1621225_at | 0.24 | 0.19 | Putative polygalacturonase |
|  | 1611320_at | 0.24 | 0.31 | UDP-glucosyl transferase 88A1 |
|  | 1613410_s_at | 0.24 | 0.4 | Similar to plastid acyl carrier protein |
|  | 1613017_s_at | 0.24 | 0.08 | Mannose-1-phosphate guanylyltransferase (GDP)s |
|  | 1619312_a_at | 0.25 | 0.16 | Acetamidase/Formamidase family protein |
|  | 1612984_at | 0.25 | 0.23 | Alpha/beta-Hydrolases superfamily protein |
|  | 1618510_at | 0.25 | 0.06 | UDP-Glycosyltransferase superfamily protein |
|  | 1622295_at | 0.25 | 0.14 | Beta-galactosidase |
|  | 1607426_at | 0.25 | 0.22 | Beta-galactosidase |
|  | 1610935_at | 0.26 | 0.08 | Ferulic acid 5-hydroxylase 1 |
|  | 1610799_at | 0.26 | 0.4 | Alpha/beta-Hydrolases superfamily protein |
|  | 1613695_at | 0.26 | 0.23 | Cytochrome P450, family 71, subfamily A, polypeptide 26 |
|  | 1611739_at | 0.26 | 0.16 | Cytochrome P450 monooxygenase CYP75C1 |
|  | 1618637_at | 0.26 | 0.28 | C2 calcium/lipid-binding plant phosphoribosyltransferase family protein |
|  | 1615571_at | 0.26 | 0.21 | Glycogen/starch synthases, ADP-glucose type |
|  | 1609732_at | 0.26 | 0.22 | Zinc-binding dehydrogenase family protein |
|  | 1620850_at | 0.26 | 0.07 | Dicarboxylate diiron protein, putative |
|  | 1620634_at | 0.26 | 0.12 | Pheophorbide a oxygenase family protein with Rieske [2Fe-2S] domain |
|  | 1613113_at | 0.26 | 0.22 | Phenylalanine ammonia-lyase |
|  | 1607417_at | 0.26 | 0.17 | Cinnamyl alcohol dehydrogenase 9 |
|  | 1621407_at | 0.27 | 0.17 | Acetamidase/Formamidase family protein |
|  | 1619383_s_at | 0.27 | 0.16 | Beta-galactosidase 3 |
|  | 1607705_at | 0.27 | 0.17 | Glycosyl hydrolase 9B13 |
|  | 1619325_at | 0.28 | 0.37 | Cytochrome P450, family 72, subfamily A, polypeptide 15 |
|  | 1613258_at | 0.28 | 0.19 | Hydrolase family protein / HAD-superfamily protein |
|  | 1614202_s_at | 0.28 | 0.48 | Alpha/beta-Hydrolases superfamily protein |
|  | 1612105_at | 0.28 | 0.09 | 3-ketoacyl-CoA synthase 11 |
|  | 1620403_at | 0.28 | 0.2 | Fatty acid desaturase 2 |
|  | 1616409_at | 0.28 | 0.23 | Cell elongation protein / DWARF1 / DIMINUTO (DIM) |
|  | 1619642_at | 0.28 | 0.31 | Phenylalanine ammonia-lyase |
|  | 1608094_at | 0.28 | 0.19 | NAD(P)-binding Rossmann-fold superfamily protein |
|  | 1617120_s_at | 0.29 | 0.47 | Phosphatidic acid phosphatase (PAP2) family protein |
|  | 1615675_at | 0.29 | 0.23 | Zinc-binding dehydrogenase family protein |
|  | 1612748_at | 0.29 | 0.46 | Disproportionating enzyme |
|  | 1606624_at | 0.29 | 0.21 | Porphobilinogen deaminase |
|  | 1616522_at | 0.29 | 0.21 | Pectinesterase |
|  | 1618409_at | 0.29 | 0.17 | Beta-1,3-glucanase-like protein |
|  | 1619954_at | 0.29 | 0.23 | Glucuronidase 2 |
|  | 1619276_at | 0.3 | 0.14 | Fatty acid/sphingolipid desaturase |
|  | 1615574_at | 0.3 | 0.13 | Plant invertase/pectin methylesterase inhibitor superfamily |
|  | 1619359_at | 0.31 | 0.22 | Cytochrome P450, family 714, subfamily A, polypeptide 1 |
|  | 1610983_at | 0.31 | 0.26 | Sulfotransferase 4C |
|  | 1614668_at | 0.31 | 0.3 | Adenine phosphoribosyl transferase 4 |
|  | 1608415_at | 0.31 | 0.11 | Sigma factor E |
|  | 1608809_at | 0.31 | 0.25 | Beta glucosidase 46 |
|  | 1613224_at | 0.31 | 0.24 | Cellulose synthase family protein |
|  | 1607069_at | 0.31 | 0.23 | Cellulose synthase like E1 |
|  | 1621112_s_at | 0.31 | 0.2 | UDP-D-apiose/UDP-D-xylose synthase 1 |
|  | 1618204_at | 0.31 | 0.45 | Enoyl-ACP reductase precursor |
|  | 1609639_at | 0.31 | 0.23 | Delta-aminolevulinic acid dehydratase |
|  | 1616224_at | 0.32 | 0.11 | Cellulose synthase-like protein CslE |
|  | 1614434_at | 0.32 | 0.35 | S-adenosyl-L-methionine-dependent methyltransferases superfamily protein |
|  | 1617585_at | 0.32 | 0.37 | Uroporphyrinogen decarboxylase |
|  | 1619808_at | 0.32 | 0.32 | NAD(P)-binding Rossmann-fold superfamily protein |
|  | 1607374_at | 0.32 | 0.29 | Glycosyl hydrolase family protein |
|  | 1606832_at | 0.33 | 0.31 | Putative glycosyl hydrolase family 5 protein |
|  | 1612289_at | 0.33 | 0.23 | Alpha/beta-Hydrolases superfamily protein |
|  | 1615431_at | 0.33 | 0.23 | AGO4-2 |
|  | 1616573_at | 0.33 | 0.22 | ATGSL12 (glucan synthase-like 12); 1,3-beta-glucan synthase/ transferase, transferring glycosyl groups |
|  | 1614903_at | 0.33 | 0.16 | Mitochondrial acyl carrier protein 3 |
|  | 1606970_at | 0.33 | 0.18 | GTP cyclohydrolase II |
|  | 1619457_at | 0.33 | 0.18 | Trehalose-6-phosphate synthase |
|  | 1608037_at | 0.33 | 0.18 | Pheophorbide a oxygenase family protein with Rieske [2Fe-2S] domain |
|  | 1619477_at | 0.33 | 0.3 | Glutamate-1-semialdehyde-2,1-aminomutase |
|  | 1609876_at | 0.33 | 0.43 | UDP-glucosyl transferase 88A1 |
|  | 1606672_at | 0.33 | 0.21 | Similar to At4g09970 |
|  | 1621309_at | 0.34 | 0.13 | OSJNBa0084K11.8 protein |
|  | 1613203_at | 0.34 | 0.32 | Class I glutamine amidotransferase-like superfamily protein |
|  | 1615577_at | 0.34 | 0.23 | Cellulose synthase family protein |
|  | 1608296_at | 0.34 | 0.2 | Acyl-[acyl-carrier protein] desaturase |
|  | 1617749_a_at | 0.34 | 0.26 | Sterol methyltransferase 1 |
|  | 1607874_at | 0.34 | 0.21 | GDP-D-mannose 3',5'-epimerase |
|  | 1617320_at | 0.34 | 0.14 | Plant invertase/pectin methylesterase inhibitor superfamily protein |
|  | 1608387_at | 0.35 | 0.18 | NAD(P)-binding Rossmann-fold superfamily protein |
|  | 1608261_at | 0.35 | 0.29 | Pectin methylesterase 3 |
|  | 1611671_at | 0.35 | 0.23 | Cellulose synthase 1 |
|  | 1609390_at | 0.35 | 0.39 | Sterol 4-alpha-methyl-oxidase 2-1 |
|  | 1619526_at | 0.35 | 0.14 | Fatty acid desaturase 8 |
|  | 1614825_at | 0.35 | 0.47 | NAD(P)-binding Rossmann-fold superfamily protein |
|  | 1620148_at | 0.36 | 0.3 | Haloacid dehalogenase (HAD) superfamily protein |
|  | 1610460_at | 0.36 | 0.27 | Cellulose synthase family protein |
|  | 1607964_s_at | 0.36 | 0.28 | Ferredoxin/thioredoxin reductase subunit A (variable subunit) 2 |
|  | 1610675_s_at | 0.36 | 0.11 | NAD(P)-linked oxidoreductase superfamily protein |
|  | 1619355_at | 0.36 | 0.26 | Endoxyloglucan transferase A3 |
|  | 1615614_at | 0.37 | 0.15 | Putative glucosyltransferase |
|  | 1611585_at | 0.37 | 0.29 | ATP-dependent caseinolytic (Clp) protease/crotonase family protein |
|  | 1622811_at | 0.38 | 0.23 | Cytochrome P450, family 89, subfamily A, polypeptide 5 |
|  | 1614707_at | 0.38 | 0.37 | Phosphorylase |
|  | 1608257_at | 0.38 | 0.29 | Sucrose-6F-phosphate phosphohydrolase family protein |
|  | 1622791_at | 0.38 | 0.26 | Cellulose synthase A4 |
|  | 1622353_at | 0.38 | 0.3 | Alpha-1,6-xylosyltransferase |
|  | 1621260_at | 0.38 | 0.28 | Phosphatidylserine decarboxylase 1 |
|  | 1610613_at | 0.38 | 0.45 | Coproporphyrinogen III oxidase |
|  | 1619051_at | 0.39 | 0.43 | Putative nitrilase-associated protein |
|  | 1617214_s_at | 0.39 | 0.22 | NAD(P)-binding Rossmann-fold superfamily protein |
|  | 1619786_at | 0.39 | 0.16 | 1,2-dihydroxy-3-keto-5-methylthiopentene dioxygenase 4 |
|  | 1615116_s_at | 0.39 | 0.4 | ATP-dependent caseinolytic (Clp) protease/crotonase family protein |
|  | 1618087_at | 0.39 | 0.39 | Similar to RelA-SpoT like protein RSH1 |
|  | 1608631_s_at | 0.39 | 0.14 | Putative alkaline alpha-galactosidase seed imbibition protein |
|  | 1608527_at | 0.39 | 0.31 | GroES-like zinc-binding alcohol dehydrogenase family protein |
|  | 1610176_at | 0.39 | 0.3 | Geranylgeranyl pyrophosphate synthase 1 |
|  | 1612989_at | 0.39 | 0.25 | Shikimate kinase like 1 |
|  | 1619468_at | 0.39 | 0.27 | Pectin methylesterase PME1 |
|  | 1616113_at | 0.4 | 0.47 | O-Glycosyl hydrolases family 17 protein |
|  | 1621572_at | 0.4 | 0.32 | Thioesterase superfamily protein |
|  | 1607911_at | 0.4 | 0.4 | Ornithine carbamoyltransferase |
|  | 1615156_at | 0.4 | 0.24 | Lipid phosphate phosphatase 2 |
|  | 1608097_at | 0.4 | 0.22 | Seed imbibition 2 |
|  | 1607727_at | 0.4 | 0.31 | Sucrose-phosphatase 1 |
|  | 1608180_at | 0.4 | 0.36 | Xyloglucan endotransglucosylase/hydrolase 7 |
|  | 1613448_at | 0.41 | 0.28 | NAD(P)-binding Rossmann-fold superfamily protein |
|  | 1621484_at | 0.41 | 0.34 | Serine acetyltransferase 2 |
|  | 1609081_at | 0.41 | 0.05 | Acireductone dioxygenase |
|  | 1620575_at | 0.41 | 0.22 | UDP-Glycosyltransferase superfamily protein |
|  | 1607590_at | 0.41 | 0.32 | Plastid transcriptionally active 17 |
|  | 1620768_at | 0.42 | 0.17 | NAD(P)-linked oxidoreductase superfamily protein |
|  | 1616349_a_at | 0.42 | 0.22 | GDP-D-mannose 3',5'-epimerase |
|  | 1617079_at | 0.42 | 0.42 | Shikimate kinase like 1 |
|  | 1608698_at | 0.43 | 0.34 | Alpha/beta-Hydrolases superfamily protein |
|  | 1606838_at | 0.43 | 0.3 | Thioesterase superfamily protein |
|  | 1607456_at | 0.44 | 0.36 | NAD(P)-binding Rossmann-fold superfamily protein |
|  | 1622630_s_at | 0.44 | 0.19 | Haloacid dehalogenase-like hydrolase (HAD) superfamily protein |
|  | 1612979_at | 0.44 | 0.4 | NAD(P)-linked oxidoreductase superfamily protein |
|  | 1614795_at | 0.44 | 0.5 | UDP-glucuronic acid decarboxylase 3 |
|  | 1619938_at | 0.44 | 0.33 | Cellulose synthase |
|  | 1614509_at | 0.44 | 0.45 | Similar to plastid acyl carrier protein |
|  | 1610577_at | 0.44 | 0.22 | Fatty acid biosynthesis 1 |
|  | 1606585_at | 0.45 | 0.22 | Aminomethyltransferase |
|  | 1612568_at | 0.45 | 0.41 | Phosphorylase |
|  | 1615421_at | 0.46 | 0.31 | SPIRAL1-like1 |
|  | 1614576_s_at | 0.46 | 0.38 | Cyanate hydratase |
|  | 1619879_s_at | 0.46 | 0.13 | Single hybrid motif superfamily protein |
|  | 1607722_at | 0.46 | 0.41 | Phosphofructokinase family protein |
|  | 1614719_at | 0.46 | 0.32 | 4-alpha-glucanotransferase |
|  | 1617939_s_at | 0.46 | 0.23 | Pectinacetylesterase family protein |
|  | 1620679_at | 0.46 | 0.48 | B-S glucosidase 44 |
|  | 1618818_at | 0.47 | 0.38 | Alpha/beta-Hydrolases superfamily protein |
|  | 1614513_at | 0.47 | 0.14 | Alpha/beta-Hydrolases superfamily protein |
|  | 1617901_s_at | 0.47 | 0.14 | Single hybrid motif superfamily protein |
|  | 1611605_s_at | 0.47 | 0.36 | Lipoic acid synthase-like protein |
|  | 1618389_at | 0.47 | 0.38 | HXXXD-type acyl-transferase family protein |
|  | 1611508_at | 0.48 | 0.19 | Thioesterase superfamily protein |
|  | 1613649_at | 0.48 | 0.34 | Acyl-CoA N-acyltransferases (NAT) superfamily protein |
|  | 1611814_at | 0.48 | 0.44 | Melibiase family protein |
|  | 1610646_s_at | 0.48 | 0.14 | Beta-amylase 1 |
|  | 1607622_at | 0.48 | 0.34 | 3-hydroxy-3-methylglutaryl CoA reductase |
|  | 1609291_at | 0.48 | 0.4 | AMP-dependent synthetase and ligase family protein |
|  | 1611897_s_at | 0.48 | 0.36 | Caffeoyl-CoA O-methyltransferase |
|  | 1621181_at | 0.48 | 0.35 | NAD(P)-binding Rossmann-fold superfamily protein |
|  | 1607357_at | 0.49 | 0.34 | Acid phosphatase/vanadium-dependent haloperoxidase-related protein |
|  | 1615174_s_at | 0.49 | 0.43 | Leucoanthocyanidin reductase 2 |
|  | 1621163_at | 0.49 | 0.31 | Putative cinnamoyl-CoA reductase |
|  | 1619011_at | 0.49 | 0.48 | 3-ketoacyl-CoA synthase 1 |
|  | 1616411_at | 0.5 | 0.34 | Met-10+ like family protein |
|  | 1619636_at | 0.5 | 0.23 | Alpha/beta-Hydrolases superfamily protein |
|  | 1619280_at | 0.5 | 0.48 | Cellulose synthase family protein |
|  | 1610047_s_at | 0.5 | 0.41 | Lipoic acid synthase 1 |
|  | 1620678_at | 0.5 | 0.42 | Flavin containing amine oxidoreductase family |
| Engery | 1611623_at | 0.04 | 0.02 | Photosystem I subunit D-2 |
|  | 1617102_at | 0.05 | 0.05 | Ribulose bisphosphate carboxylase small chain |
|  | 1621495_at | 0.08 | 0.02 | Alpha carbonic anhydrase 1 |
|  | 1618061_a_at | 0.09 | 0.08 | Transketolase |
|  | 1612075_at | 0.1 | 0.05 | Ferric reduction oxidase 6 |
|  | 1620504_at | 0.13 | 0.1 | Nodulin MtN3 family protein |
|  | 1607800_at | 0.15 | 0.08 | Putative cytochrome c oxidoreductase |
|  | 1619629_at | 0.15 | 0.03 | Light-harvesting chlorophyll B-binding protein 3 |
|  | 1620654_at | 0.16 | 0.32 | Transketolase |
|  | 1606445_a_at | 0.16 | 0.13 | Photosynthetic electron transfer C |
|  | 1622109_at | 0.16 | 0.07 | Photosystem II 11 kDa protein-related |
|  | 1616940_s_at | 0.16 | 0.03 | Light-harvesting chlorophyll-protein complex I subunit A4 |
|  | 1611860_at | 0.16 | 0.03 | Chlorophyll a-b binding protein CP24 10A, chloroplast precursor |
|  | 1621351_s_at | 0.16 | 0.03 | Light-harvesting chlorophyll-protein complex II subunit B1 |
|  | 1612480_at | 0.19 | 0.07 | PsbQ-like 2 |
|  | 1621038_at | 0.19 | 0.08 | Photosystem II light harvesting complex gene B1B2 |
|  | 1620884_at | 0.2 | 0.07 | Serine/threonine-protein kinase SNT7, chloroplast precursor |
|  | 1613991_at | 0.22 | 0.08 | Photosystem ii core complex proteins psby, chloroplast (L-arginine metabolising enzyme) (L-ame) |
|  | 1608311_at | 0.22 | 0.05 | Chlorophyll a/b-binding protein |
|  | 1622074_at | 0.23 | 0.18 | Phosphoenolpyruvate carboxylase kinase 1 |
|  | 1620694_at | 0.26 | 0.24 | Dihydrolipoyl dehydrogenase |
|  | 1609044_at | 0.26 | 0.04 | Light-harvesting chlorophyll-protein complex I subunit A4 |
|  | 1614720_at | 0.27 | 0.08 | ACD1-like |
|  | 1611924_at | 0.27 | 0.08 | Photosystem I subunit G |
|  | 1607056_at | 0.27 | 0.16 | Magnesium chelatase i2 |
|  | 1618107_at | 0.28 | 0.07 | ACD1-like |
|  | 1614699_at | 0.29 | 0.28 | Putative membrane lipoprotein |
|  | 1614655_at | 0.29 | 0.15 | P-loop containing nucleoside triphosphate hydrolases superfamily protein |
|  | 1614740_at | 0.29 | 0.06 | Rubisco activase |
|  | 1614156_at | 0.31 | 0.02 | High cyclic electron flow 1 |
|  | 1607516_at | 0.32 | 0.09 | Photosystem II subunit O-2 |
|  | 1612085_at | 0.33 | 0.11 | Photosystem II reaction center W |
|  | 1609784_s_at | 0.33 | 0.15 | Oxidoreductase, zinc-binding dehydrogenase family protein |
|  | 1607514_at | 0.33 | 0.15 | Chloroplast thylakoid lumen protein |
|  | 1613843_at | 0.33 | 0.21 | Photosystem II reaction center PsbP family protein |
|  | 1612645_at | 0.33 | 0.14 | ATP synthase |
|  | 1609335_at | 0.34 | 0.17 | Putative oxygen evolving enhancer protein |
|  | 1620551_s_at | 0.34 | 0.08 | Rubisco activase |
|  | 1608606_at | 0.34 | 0.19 | Pentapeptide repeat-containing protein |
|  | 1618249_at | 0.35 | 0.13 | Aldehyde dehydrogenase 11A3 |
|  | 1609678_at | 0.37 | 0.12 | Fructose-bisphosphate aldolase |
|  | 1610491_at | 0.37 | 0.22 | Putative ribulose-1,5-bisphosphate carboxylase/oxygenase small subunit N-methyltransferase I |
|  | 1607673_at | 0.37 | 0.1 | Magnesium-chelatase subunit chlH, chloroplast, putative |
|  | 1618617_at | 0.37 | 0.27 | Tetratricopeptide repeat (TPR)-like superfamily protein |
|  | 1616002_s_at | 0.38 | 0.1 | Fructose-bisphosphate aldolase |
|  | 1622575_s_at | 0.38 | 0.41 | Pyruvate dehydrogenase kinase |
|  | 1616560_at | 0.38 | 0.16 | Photosystem I light harvesting complex gene 6 |
|  | 1607961_at | 0.39 | 0.16 | Photosystem II 5 kDa protein, chloroplast precursor |
|  | 1613939_at | 0.4 | 0.48 | Galactose mutarotase-like superfamily protein |
|  | 1611645_at | 0.4 | 0.19 | Photosystem II 22 kDa protein |
|  | 1611901_s_at | 0.42 | 0.32 | Carbonic anhydrase 2 |
|  | 1610360_at | 0.42 | 0.11 | Photosystem II subunit X |
|  | 1618116_s_at | 0.42 | 0.05 | Chlorophyll A/B binding protein 1 |
|  | 1620184_at | 0.43 | 0.16 | Malate dehydrogenase |
|  | 1621944_at | 0.43 | 0.14 | Fructose-bisphosphate aldolase |
|  | 1619903_at | 0.43 | 0.12 | Photosystem I light harvesting complex gene 1 |
|  | 1610245_at | 0.43 | 0.08 | Chlorophyll A/B binding protein 1 |
|  | 1618497_at | 0.44 | 0.17 | Glycine decarboxylase P-protein 2 |
|  | 1613595_at | 0.45 | 0.4 | Transketolase |
|  | 1616325_at | 0.45 | 0.27 | Phosphoenolpyruvate carboxylase |
|  | 1608100_at | 0.45 | 0.45 | Phosphoenolpyruvate carboxylase |
|  | 1613867_at | 0.46 | 0.06 | High cyclic electron flow 1 |
|  | 1621532_at | 0.47 | 0.15 | Photosystem I subunit K |
|  | 1620744_at | 0.48 | 0.21 | Plastocyanin A, chloroplast, putative |
|  | 1622065_at | 0.49 | 0.18 | Fructose-1,6-bisphosphatase, chloroplast precursor |
|  | 1620802_at | 0.49 | 0.43 | pyruvate dehydrogenase E1 alpha |
|  | 1620292_at | 0.49 | 0.15 | Ultraviolet-B-repressible protein |
|  | 1611093_at | 0.5 | 0.23 | Pyruvate dehydrogenase kinase |
|  | 1619503_at | 0.5 | 0.31 | Photosystem II stability/assembly factor, chloroplast (HCF136) |
|  | 1614598_at | 0.5 | 0.14 | Light harvesting complex photosystem II |
|  | 1615602_at | 0.5 | 0.22 | ATPase, F1 complex, gamma subunit protein |
| Storage protein  cell cyecle and DNA processing | 1618086_at | 0.08 | 0.04 | Phosphorylase superfamily protein |
|  | 1618511_at | 0.07 | 0.03 | Similarity to protein ref:NP_197631.1 (A.thaliana) |
|  | 1617240_at | 0.07 | 0.07 | Putative histone acetyltransferase |
|  | 1621976_at | 0.08 | 0.05 | DNA glycosylase superfamily protein |
|  | 1608794_a_at | 0.1 | 0.08 | Cyclin D1 |
|  | 1612468_at | 0.15 | 0.15 | Cyclin D1;1 |
|  | 1616638_at | 0.19 | 0.13 | Cyclin D3;3 |
|  | 1608380_at | 0.22 | 0.09 | Histone superfamily protein |
|  | 1620212_at | 0.22 | 0.18 | Cyclin-dependent kinase |
|  | 1610135_at | 0.24 | 0.25 | Cyclin dependent kinase inhibitor |
|  | 1613041_at | 0.25 | 0.18 | Histone superfamily protein |
|  | 1616453_at | 0.25 | 0.15 | Cyclin-dependent kinase B2;1 |
|  | 1608305_at | 0.26 | 0.21 | Putative seed specific protein |
|  | 1608927_at | 0.28 | 0.24 | Histone H2A |
|  | 1618097_at | 0.29 | 0.32 | Histone superfamily protein \| |
|  | 1607575_at | 0.29 | 0.26 | Photolyase/blue-light receptor 2 |
|  | 1612614_at | 0.31 | 0.26 | Histone deacetylase 8 |
|  | 1615878_at | 0.31 | 0.34 | Histone-lysine N-methyltransferase ASHH3 |
|  | 1620332_at | 0.33 | 0.26 | Histone superfamily protein |
|  | 1614452_at | 0.35 | 0.46 | SET domain protein |
|  | 1615788_at | 0.35 | 0.33 | Protein kinase superfamily protein |
|  | 1609715_s_at | 0.38 | 0.47 | Histone H2A |
|  | 1607026_s_at | 0.4 | 0.5 | Winged-helix DNA-binding transcription factor family protein |
|  | 1614217_at | 0.4 | 0.4 | Domains rearranged methyltransferase 2 |
|  | 1619207_at | 0.41 | 0.34 | DNA polymerase delta small subunit |
|  | 1610096_at | 0.43 | 0.49 | Histone superfamily protein |
|  | 1618944_at | 0.44 | 0.4 | Histone superfamily protein |
|  | 1609334_at | 0.45 | 0.48 | Replication factor C 2 |
|  | 1606509_at | 0.46 | 0.23 | NAD(P)-linked oxidoreductase superfamily protein |
|  | 1608056_at | 0.49 | 0.35 | Histone H2A |
| Transcription | 1614061_at | 0.03 | 0.02 | B-box type zinc finger protein with CCT domain |
|  | 1611920_at | 0.04 | 0.04 | MYB transcription factor |
|  | 1615196_at | 0.04 | 0.02 | Zinc finger protein 8 |
|  | 1613445_at | 0.06 | 0.04 | Mutant cincinnata |
|  | 1610657_at | 0.06 | 0.06 | Homeobox-leucine zipper protein family |
|  | 1609484_at | 0.07 | 0.05 | Beta HLH protein 71 |
|  | 1618884_at | 0.09 | 0.08 | Myb-like, HTH transcriptional regulator family protein |
|  | 1609874_at | 0.09 | 0.06 | CONSTANS-like 2 |
|  | 1618504_at | 0.09 | 0.06 | Myc anthocyanin regulatory protein |
|  | 1613486_at | 0.09 | 0.07 | MYB transcription factor |
|  | 1616202_at | 0.1 | 0.08 | Squalene epoxidase 3 |
|  | 1619987_at | 0.12 | 0.06 | Sigma factor A |
|  | 1618519_at | 0.12 | 0.11 | GATA transcription factor 16 |
|  | 1617790_at | 0.12 | 0.04 | Phytochrome-interacting factor7 |
|  | 1609798_at | 0.12 | 0.1 | Duplicated homeodomain-like superfamily protein |
|  | 1619418_at | 0.13 | 0.05 | Putative bHLH transcription factor |
|  | 1621872_s_at | 0.13 | 0.14 | Myb-like transcription factor family protein |
|  | 1622853_at | 0.13 | 0.09 | Similar to Histone-lysine N-methyltransferase ATX2 |
|  | 1609148_at | 0.13 | 0.04 | Squamosa promoter binding protein-like 4 |
|  | 1609496_at | 0.14 | 0.14 | Sequence-specific DNA binding transcription factors |
|  | 1619226_at | 0.14 | 0.09 | GOLDEN2-like 2 |
|  | 1610349_s_at | 0.15 | 0.07 | Similar to SIGB (SIGMA FACTOR B), DNA binding / DNA-directed RNA polymerase/ transcription factor |
|  | 1606951_at | 0.15 | 0.07 | B-box type zinc finger family protein |
|  | 1608582_at | 0.15 | 0.2 | Similar to ZFP4 (ZINC FINGER PROTEIN 4) |
|  | 1611042_at | 0.16 | 0.19 | Lipid-binding serum glycoprotein family protein |
|  | 1617362_at | 0.16 | 0.09 | Nuclear factor Y, subunit B3 |
|  | 1606591_at | 0.16 | 0.16 | GRAS family transcription factor |
|  | 1620278_at | 0.16 | 0.13 | Integrase-type DNA-binding superfamily protein |
|  | 1622864_at | 0.17 | 0.15 | BHLH protein family-like |
|  | 1617998_at | 0.18 | 0.22 | myb domain protein |
|  | 1617012_at | 0.18 | 0.18 | Integrase-type DNA-binding superfamily protein |
|  | 1619204_at | 0.18 | 0.06 | RAD-like 1 |
|  | 1622209_at | 0.18 | 0.11 | Plant-specific transcription factor YABBY family protein |
|  | 1622273_at | 0.18 | 0.21 | BZIP protein |
|  | 1614416_at | 0.19 | 0.14 | MYB-like transcription factor |
|  | 1606914_at | 0.19 | 0.13 | B-box zinc finger family protein |
|  | 1615847_at | 0.2 | 0.12 | Similar to SIGB (SIGMA FACTOR B), DNA binding / DNA-directed RNA polymerase/ transcription factor |
|  | 1617694_at | 0.2 | 0.17 | GBF's pro-rich region-interacting factor 1 |
|  | 1611070_at | 0.2 | 0.08 | SSXT family protein |
|  | 1620083_at | 0.21 | 0.42 | Basic leucine zipper 9 |
|  | 1614331_at | 0.21 | 0.13 | Polynucleotidyl transferase |
|  | 1614532_at | 0.21 | 0.13 | High chlorophyll fluorescent 107 |
|  | 1609206_at | 0.22 | 0.09 | Homeodomain-like superfamily protein |
|  | 1606491_at | 0.22 | 0.22 | WUSCHEL related homeobox 4 |
|  | 1613799_at | 0.22 | 0.13 | Integrase-type DNA-binding superfamily protein |
|  | 1612362_at | 0.23 | 0.09 | Plant-specific transcription factor YABBY family protein |
|  | 1609175_at | 0.24 | 0.25 | Putative membrane lipoprotein |
|  | 1619029_at | 0.24 | 0.24 | Abscisic acid responsive elements-binding factor 2 |
|  | 1613698_at | 0.24 | 0.15 | Putative ethylene response factor 4 |
|  | 1614909_at | 0.25 | 0.24 | MYC1 |
|  | 1617042_at | 0.25 | 0.28 | Putative transcription factor |
|  | 1607523_at | 0.25 | 0.11 | TCP1 protein |
|  | 1621238_at | 0.26 | 0.2 | Putative transcription factor |
|  | 1613842_at | 0.26 | 0.29 | C2H2-like zinc finger protein |
|  | 1619453_s_at | 0.26 | 0.25 | Homeobox 1 |
|  | 1615011_at | 0.26 | 0.23 | Homeobox 1 |
|  | 1611381_at | 0.26 | 0.17 | Similar to putative At5g37260 |
|  | 1613239_at | 0.26 | 0.22 | MYB transcription factor MYB118 |
|  | 1609122_at | 0.26 | 0.34 | Metallo-hydrolase/oxidoreductase superfamily protein |
|  | 1619362_at | 0.27 | 0.11 | RNApolymerase sigma-subunit F |
|  | 1610370_s_at | 0.27 | 0.23 | Early nodulin-like protein 17 |
|  | 1609442_at | 0.27 | 0.11 | Sequence-specific DNA binding transcription factors |
|  | 1620708_at | 0.27 | 0.04 | RAD-like 5 |
|  | 1614723_at | 0.27 | 0.22 | Growth-regulating factor 3 |
|  | 1620877_at | 0.27 | 0.1 | GATA transcription factor 9 |
|  | 1614656_at | 0.27 | 0.33 | BEL1-like homeodomain 2 |
|  | 1610202_at | 0.27 | 0.14 | Ribonuclease 1 |
|  | 1607898_at | 0.27 | 0.17 | Putative 3' exoribonuclease |
|  | 1606781_at | 0.28 | 0.46 | YEATS family protein |
|  | 1617092_at | 0.28 | 0.26 | MYB transcription factor |
|  | 1620536_at | 0.28 | 0.31 | BEL1-related homeotic protein 13 |
|  | 1611709_at | 0.29 | 0.19 | TCP family transcription factor |
|  | 1614483_at | 0.29 | 0.19 | TCP-domain protein |
|  | 1615068_at | 0.29 | 0.31 | POX (plant homeobox) family protein |
|  | 1608281_at | 0.29 | 0.28 | P-loop containing nucleoside triphosphate hydrolases superfamily protein |
|  | 1614432_at | 0.29 | 0.21 | Putative ribonuclease E |
|  | 1616675_at | 0.3 | 0.2 | Transcription factor |
|  | 1616946_at | 0.31 | 0.1 | Sigma factor E |
|  | 1610512_at | 0.31 | 0.43 | MYB transcription factor |
|  | 1614864_at | 0.32 | 0.22 | Sigma factor |
|  | 1614298_at | 0.32 | 0.23 | Plant-specific transcription factor YABBY family protein |
|  | 1619251_at | 0.32 | 0.21 | B-box type zinc finger family protein |
|  | 1612264_at | 0.32 | 0.44 | MYB transcription factor |
|  | 1621798_at | 0.33 | 0.3 | Integrase-type DNA-binding superfamily protein |
|  | 1619410_at | 0.33 | 0.28 | HDZip I protein |
|  | 1622245_at | 0.33 | 0.16 | Basic helix-loop-helix (bHLH) DNA-binding superfamily protein |
|  | 1610120_at | 0.33 | 0.22 | Short vegetative phase protein |
|  | 1616278_a_at | 0.33 | 0.21 | RNA-binding (RRM/RBD/RNP motifs) family protein |
|  | 1618219_at | 0.34 | 0.23 | Plant-specific transcription factor YABBY family protein |
|  | 1618145_at | 0.34 | 0.33 | Homeodomain leucine zipper protein HDZ2 |
|  | 1621794_at | 0.35 | 0.45 | DNA-binding storekeeper protein-related |
|  | 1607278_at | 0.35 | 0.46 | SWIB/MDM2 domain superfamily protein |
|  | 1611914_at | 0.36 | 0.39 | RNA polymerase Rpb7-like, N-terminal domain |
|  | 1617580_at | 0.36 | 0.13 | Sigma factor E |
|  | 1621314_s_at | 0.36 | 0.35 | Related to AP2 4 |
|  | 1616094_at | 0.37 | 0.32 | VvMYBPA1 |
|  | 1617350_at | 0.37 | 0.31 | Early nodulin-like protein 17 |
|  | 1606707_at | 0.37 | 0.41 | Transcription factor IIB |
|  | 1609116_at | 0.37 | 0.21 | RNA-binding (RRM/RBD/RNP motifs) family protein |
|  | 1610882_s_at | 0.38 | 0.4 | Basic helix-loop-helix (bHLH) DNA-binding superfamily protein |
|  | 1609859_at | 0.38 | 0.41 | DEA(D/H)-box RNA helicase family protein |
|  | 1614235_at | 0.39 | 0.22 | Homeobox-leucine zipper protein HDG1 |
|  | 1617493_at | 0.39 | 0.38 | Homeobox protein 22 |
|  | 1617626_at | 0.39 | 0.34 | Basic helix-loop-helix (bHLH) DNA-binding superfamily protein |
|  | 1619574_at | 0.4 | 0.31 | Transducin family protein / WD-40 repeat family protein |
|  | 1609991_at | 0.4 | 0.25 | Putative zinc finger protein |
|  | 1619977_at | 0.4 | 0.36 | K-box region and MADS-box transcription factor family protein |
|  | 1618129_at | 0.4 | 0.29 | Transcription factor-related |
|  | 1618437_at | 0.41 | 0.38 | Homeobox 1 |
|  | 1620966_at | 0.42 | 0.3 | RmlC-like cupins superfamily protein |
|  | 1619627_at | 0.43 | 0.47 | Transcriptional activator DEMETER |
|  | 1617877_at | 0.44 | 0.31 | GATA type zinc finger transcription factor family protein |
|  | 1607284_at | 0.44 | 0.32 | POX (plant homeobox) family protein |
|  | 1617630_at | 0.45 | 0.31 | Tubulin/FtsZ family protein |
|  | 1615524_at | 0.45 | 0.41 | MADS-box protein |
|  | 1613366_at | 0.46 | 0.41 | TCP family transcription factor 4 |
|  | 1621474_at | 0.46 | 0.26 | DEAD box RNA helicase (RH3) |
|  | 1615616_at | 0.46 | 0.27 | Saccharopine dehydrogenase |
|  | 1611866_at | 0.46 | 0.4 | RNA-binding (RRM/RBD/RNP motifs) family protein |
|  | 1611536_at | 0.48 | 0.4 | Yippee family putative zinc-binding protein |
|  | 1620175_at | 0.48 | 0.49 | WRKY DNA-binding protein 3 |
|  | 1611218_at | 0.49 | 0.37 | Transcription regulators |
|  | 1607494_at | 0.49 | 0.34 | KNOTTED-like homeobox of Arabidopsis thaliana 7 |
|  | 1615191_at | 0.49 | 0.49 | Ribonuclease III family protein |
|  | 1612742_s_at | 0.5 | 0.35 | GATA transcription factor 27 |
| Protein synthesis | 1607017_at | 0.16 | 0.07 | Similar to protein At3g53470 |
|  | 1610546_at | 0.17 | 0.12 | Elongation factor Ts family protein |
|  | 1614138_at | 0.19 | 0.07 | Ribosomal protein PSRP-3/Ycf65 |
|  | 1608691_at | 0.2 | 0.1 | Ribosomal protein L13 family protein |
|  | 1611933_at | 0.2 | 0.08 | RNA-binding (RRM/RBD/RNP motifs) family protein |
|  | 1620684_at | 0.2 | 0.07 | 30S ribosomal protein S6 alpha, chloroplast precursor |
|  | 1608376_at | 0.21 | 0.09 | Similar to ribosomal protein S17 isoform 1 |
|  | 1610136_a_at | 0.21 | 0.3 | Similar to Elongation factor Tu, chloroplastic |
|  | 1622348_s_at | 0.22 | 0.1 | Ribosomal protein L13 family protein |
|  | 1613729_at | 0.24 | 0.15 | Ribosomal protein S17 |
|  | 1614745_at | 0.25 | 0.09 | 50S ribosomal protein L35 |
|  | 1620854_at | 0.25 | 0.09 | 50S ribosomal protein L34 |
|  | 1621285_at | 0.25 | 0.11 | Ribosomal L5P family protein |
|  | 1613550_at | 0.26 | 0.17 | 50S ribosomal protein L21 |
|  | 1620434_at | 0.28 | 0.21 | 30S ribosomal protein S1-like |
|  | 1606797_at | 0.29 | 0.18 | Similar to 50S ribosomal protein L17 |
|  | 1615910_at | 0.29 | 0.39 | Ribosomal L18p/L5e family protein |
|  | 1608205_at | 0.29 | 0.06 | Similar to Elongation factor Tu, chloroplastic |
|  | 1609685_at | 0.3 | 0.21 | Ribosome-binding factor A family protein |
|  | 1616390_at | 0.31 | 0.13 | Ribosomal protein S10p/S20e family protein |
|  | 1607466_at | 0.31 | 0.11 | Ribosomal protein L6 family |
|  | 1613645_at | 0.32 | 0.16 | Ribosomal protein L13 family protein |
|  | 1621569_at | 0.32 | 0.24 | Ribosomal protein L12-A |
|  | 1611293_at | 0.33 | 0.17 | Ribosomal protein L3 family protein |
|  | 1611472_at | 0.33 | 0.14 | Ribosomal protein L19 |
|  | 1622803_at | 0.33 | 0.13 | Ribosomal protein L10 family protein |
|  | 1606900_at | 0.34 | 0.16 | Ribosomal protein S13/S18 family |
|  | 1620553_at | 0.34 | 0.19 | Phenylalanyl-tRNA synthetase class IIc family protein |
|  | 1606926_at | 0.35 | 0.25 | Glutamyl-tRNA(Gln) and/or aspartyl-tRNA(Asn) amidotransferase, C subunit |
|  | 1607273_at | 0.35 | 0.14 | Plastid-specific 50S ribosomal protein 6 |
|  | 1608490_s_at | 0.35 | 0.12 | Glutamyl-tRNA reductase |
|  | 1618168_s_at | 0.36 | 0.42 | Ribosomal protein L2 family |
|  | 1610867_at | 0.37 | 0.43 | Similar to structural constituent of ribosome |
|  | 1609719_at | 0.38 | 0.1 | Translation elongation factor EFG/EF2 protein |
|  | 1616667_a_at | 0.39 | 0.26 | Ribosomal L29 family protein |
|  | 1607287_a_at | 0.39 | 0.16 | Plastid-specific 50S ribosomal protein 5, chloroplast precursor |
|  | 1607878_at | 0.39 | 0.15 | Glutamyl-tRNA reductase |
|  | 1621399_s_at | 0.4 | 0.18 | Ribosome recycling factor, chloroplast precursor |
|  | 1606458_at | 0.4 | 0.3 | Ribosomal protein L9 |
|  | 1616948_at | 0.4 | 0.24 | Similar to putative tryptophanyl-tRNA synthetase |
|  | 1615609_at | 0.41 | 0.18 | Ribosomal protein L15 |
|  | 1619291_at | 0.41 | 0.15 | Structural constituent of ribosome |
|  | 1613959_at | 0.41 | 0.44 | Ribosomal protein L9/RNase H1 |
|  | 1612081_at | 0.41 | 0.14 | Similarity to 30s ribosomal protein s1 |
|  | 1615542_at | 0.41 | 0.49 | Collagen alpha-1(XV) chain precursor |
|  | 1606971_at | 0.42 | 0.3 | Ribosomal protein L30/L7 family protein |
|  | 1622495_at | 0.42 | 0.23 | Ribosomal protein S9 |
|  | 1610733_at | 0.44 | 0.31 | Putative translation elongation factor |
|  | 1619816_at | 0.45 | 0.22 | Ribosomal protein L31 |
|  | 1616096_s_at | 0.46 | 0.3 | Ribosomal protein S21 family protein |
|  | 1613561_at | 0.46 | 0.34 | Glycine-tRNA ligases |
|  | 1607422_s_at | 0.47 | 0.26 | Ribosomal L28 family |
|  | 1619536_at | 0.47 | 0.39 | High chlorophyll fluorescent 109 |
|  | 1614439_at | 0.47 | 0.27 | GLU-ADT subunit B |
|  | 1607285_s_at | 0.47 | 0.3 | Class II aaRS and biotin synthetases superfamily protein |
|  | 1617122_s_at | 0.48 | 0.21 | 30S ribosomal protein S31 |
|  | 1610716_at | 0.48 | 0.23 | Chloroplast 30S ribosomal protein S20, putative |
|  | 1620113_at | 0.48 | 0.13 | Translation elongation factor EFG/EF2 protein |
|  | 1618847_s_at | 0.5 | 0.27 | Ribosomal protein L1 |
| Protein fate | 1615999_at | 0.04 | 0.02 | RING/U-box superfamily protein |
|  | 1608319_at | 0.05 | 0.03 | F-BOX WITH WD-40 2 |
|  | 1606531_at | 0.05 | 0.04 | Subtilase family protein |
|  | 1612443_at | 0.06 | 0.04 | Putative CBL-interacting protein kinase 2 |
|  | 1607653_at | 0.06 | 0.04 | Aspartyl protease family protein |
|  | 1620287_at | 0.08 | 0.06 | Subtilase family protein |
|  | 1616401_at | 0.09 | 0.09 | Putative serine/threonine protein kinase |
|  | 1612873_at | 0.1 | 0.15 | Subtilisin-like serine protease 2 |
|  | 1606762_at | 0.11 | 0.1 | Serine carboxypeptidase-like 40 |
|  | 1607227_s_at | 0.12 | 0.03 | Chaperonin-like RbcX protein |
|  | 1608824_at | 0.12 | 0.05 | Similarity to At1g32520 |
|  | 1616850_at | 0.13 | 0.1 | Serine carboxypeptidase-like 17 |
|  | 1614293_at | 0.14 | 0.05 | Chaperone DnaJ-domain superfamily protein |
|  | 1619775_at | 0.14 | 0.08 | Aspartyl protease family protein |
|  | 1607752_at | 0.14 | 0.1 | Aspartyl protease family protein |
|  | 1620896_at | 0.15 | 0.13 | Subtilase 1.3 |
|  | 1618083_at | 0.16 | 0.09 | Chaperone DnaJ-domain superfamily protein |
|  | 1617344_at | 0.17 | 0.19 | NSP-interacting kinase 3 |
|  | 1612525_at | 0.17 | 0.06 | Aspartyl protease family protein |
|  | 1609490_s_at | 0.18 | 0.12 | Alpha/beta-Hydrolases superfamily protein |
|  | 1613583_at | 0.18 | 0.07 | Subtilase family protein |
|  | 1622218_at | 0.19 | 0.08 | Similar to membrane-associated zinc metalloprotease, putative isoform 1 |
|  | 1611462_at | 0.2 | 0.07 | Arm repeat containing protein |
|  | 1620013_at | 0.2 | 0.32 | RING/U-box superfamily protein |
|  | 1608213_at | 0.21 | 0.08 | Subtilisin-like serine protease 3 |
|  | 1619560_at | 0.22 | 0.11 | Peptidyl-prolyl cis-trans isomerase |
|  | 1619169_at | 0.22 | 0.07 | Serine carboxypeptidase-like 27 |
|  | 1616222_at | 0.23 | 0.1 | DnaJ/Hsp40 cysteine-rich domain superfamily protein |
|  | 1617962_at | 0.23 | 0.19 | Similar to At1g60010/T2K10_6 |
|  | 1613085_at | 0.23 | 0.11 | Phototropin 1 |
|  | 1612531_at | 0.23 | 0.28 | Aspartyl protease family protein |
|  | 1615913_at | 0.24 | 0.34 | Double Clp-N motif-containing P-loop nucleoside triphosphate hydrolases superfamily protein |
|  | 1607003_at | 0.24 | 0.09 | DnaJ/Hsp40 cysteine-rich domain superfamily protein |
|  | 1608829_at | 0.25 | 0.2 | Protein phosphatase 2C family protein |
|  | 1616046_at | 0.26 | 0.2 | Glycosyltransferase family 29 (sialyltransferase) family protein |
|  | 1610938_at | 0.26 | 0.19 | Ser/Thr kinase |
|  | 1612634_at | 0.27 | 0.41 | Chaperonin-60alpha |
|  | 1613856_at | 0.28 | 0.22 | Leucine-rich repeat protein kinase family protein |
|  | 1612593_at | 0.28 | 0.37 | Similar to stpk1 protein kinase |
|  | 1609737_at | 0.28 | 0.18 | Plant U-box 26 |
|  | 1611254_s_at | 0.28 | 0.39 | RING/U-box superfamily protein |
|  | 1621456_at | 0.28 | 0.2 | RING/U-box superfamily protein |
|  | 1610231_at | 0.29 | 0.38 | RING/U-box superfamily protein |
|  | 1620841_at | 0.3 | 0.39 | HSP20-like chaperones superfamily protein |
|  | 1619556_at | 0.3 | 0.12 | Peptidyl-prolyl cis-trans isomerase |
|  | 1606754_at | 0.3 | 0.4 | Serine carboxypeptidase-like 35 |
|  | 1607831_at | 0.31 | 0.34 | Evolutionarily conserved C-terminal region 11 |
|  | 1607790_at | 0.32 | 0.42 | DNAJ heat shock N-terminal domain-containing protein |
|  | 1622013_at | 0.32 | 0.21 | Xylulose kinase-1 |
|  | 1613223_at | 0.33 | 0.33 | ATP-dependent Clp protease proteolytic subunit |
|  | 1622489_at | 0.34 | 0.44 | HSP20-like chaperones superfamily protein |
|  | 1617187_at | 0.34 | 0.24 | Ubiquitin family protein |
|  | 1611891_at | 0.34 | 0.13 | Serine carboxypeptidase-like 20 |
|  | 1606844_at | 0.34 | 0.23 | Subtilisin-like serine endopeptidase family protein |
|  | 1616949_at | 0.34 | 0.19 | FTSH protease 11 |
|  | 1607464_at | 0.34 | 0.36 | Peptidase M50 family protein |
|  | 1618234_at | 0.35 | 0.41 | Diadenosine 5,5-P1,P4-tetraphosphate hydrolase |
|  | 1619941_at | 0.35 | 0.27 | CBL-interacting protein kinase 1 |
|  | 1618990_at | 0.35 | 0.43 | OTU-like cysteine protease family protein |
|  | 1613980_at | 0.35 | 0.29 | Subtilase family protein |
|  | 1614692_at | 0.36 | 0.31 | Chaperone protein htpG family protein |
|  | 1616097_at | 0.36 | 0.18 | Peptidyl-prolyl cis-trans isomerase |
|  | 1616239_at | 0.36 | 0.27 | Receptor-like protein kinase 1 |
|  | 1608656_at | 0.36 | 0.16 | Putative C3HC4-type RING zinc finger protein |
|  | 1608644_at | 0.36 | 0.35 | RING/U-box superfamily protein |
|  | 1615690_at | 0.36 | 0.21 | Serine carboxypeptidase-like 20 |
|  | 1613057_at | 0.37 | 0.25 | Chaperone protein htpG family protein |
|  | 1617986_at | 0.37 | 0.33 | DnaJ-like protein isoform |
|  | 1607832_at | 0.37 | 0.2 | SBP (S-ribonuclease binding protein) family protein |
|  | 1609802_at | 0.37 | 0.29 | Subtilase family protein |
|  | 1611162_at | 0.38 | 0.4 | Peptidyl-prolyl cis-trans isomerase |
|  | 1619615_s_at | 0.38 | 0.32 | Chaperonin 20 |
|  | 1615393_s_at | 0.38 | 0.32 | DNAJ-like 20 |
|  | 1607816_at | 0.38 | 0.22 | STRUBBELIG-receptor family 8 |
|  | 1616531_at | 0.39 | 0.22 | DC1 domain-containing protein |
|  | 1606439_s_at | 0.39 | 0.34 | EIN3-binding F-box protein 2 |
|  | 1617099_at | 0.4 | 0.47 | Co-chaperone GrpE family protein |
|  | 1613405_at | 0.4 | 0.31 | Peptidyl-prolyl cis-trans isomerase |
|  | 1607070_at | 0.4 | 0.22 | Casein lytic proteinase B3 |
|  | 1611516_at | 0.4 | 0.4 | Protein kinase superfamily protein |
|  | 1611363_at | 0.41 | 0.28 | Xylulose kinase-1 |
|  | 1609644_at | 0.41 | 0.2 | Alpha/beta-Hydrolases superfamily protein |
|  | 1606978_s_at | 0.42 | 0.41 | Chaperone DnaJ-domain superfamily protein |
|  | 1610964_at | 0.42 | 0.45 | F-BOX WITH WD-40 2 |
|  | 1615289_at | 0.42 | 0.24 | ATPase |
|  | 1620705_a_at | 0.43 | 0.47 | Serine carboxypeptidase-like 44 |
|  | 1614524_at | 0.43 | 0.15 | Low psii accumulation2 |
|  | 1615946_s_at | 0.43 | 0.38 | ATP-dependent Clp protease proteolytic subunit |
|  | 1620538_at | 0.43 | 0.47 | Putative O-sialoglycoprotein endopeptidase |
|  | 1615731_at | 0.44 | 0.3 | Chaperone protein htpG family protein |
|  | 1606621_at | 0.44 | 0.26 | Serine/threonine-protein kinase |
|  | 1607463_at | 0.44 | 0.29 | Serine carboxypeptidase-like 17 |
|  | 1618912_at | 0.45 | 0.43 | RING/U-box superfamily protein |
|  | 1621652_at | 0.45 | 0.4 | Flavonoid 3'-hydroxylase |
|  | 1617221_s_at | 0.45 | 0.47 | Receptor-like protein kinase 1 |
|  | 1617767_s_at | 0.45 | 0.31 | Ubiquitin carrier protein |
|  | 1611263_at | 0.45 | 0.43 | Ureidoglycolate amidohydrolase |
|  | 1617445_s_at | 0.46 | 0.32 | Similar to Hsp70 interacting protein |
|  | 1607399_at | 0.46 | 0.45 | SUGAR-INSENSITIVE 3 |
|  | 1607818_at | 0.47 | 0.42 | Tubulin folding cofactor A (KIESEL) |
|  | 1607226_at | 0.48 | 0.35 | P-loop containing nucleoside triphosphate hydrolases superfamily protein |
|  | 1615008_at | 0.48 | 0.42 | Hsp70 interacting protein/thioredoxin chimera |
|  | 1612115_at | 0.48 | 0.42 | Calcineurin-like metallo-phosphoesterase superfamily protein |
|  | 1621230_at | 0.49 | 0.5 | Chaperone DnaJ-domain superfamily protein |
|  | 1622380_at | 0.49 | 0.48 | Peptidase M20/M25/M40 family protein |
|  | 1620475_at | 0.49 | 0.32 | Methionyl aminopeptidase-like protein |
|  | 1616435_at | 0.5 | 0.41 | Chaperonin 60 beta |
|  | 1616555_s_at | 0.5 | 0.29 | Pherophorin-dz1 protein precursor |
|  | 1617307_at | 0.5 | 0.45 | RING-H2 finger A2A |
| Protein with binding function | 1609391_s_at | 0.1 | 0.14 | Blue copper protein precursor |
|  | 1617317_at | 0.16 | 0.14 | C2H2-like zinc finger protein |
|  | 1607812_at | 0.16 | 0.2 | Concanavalin A-like lectin family protein |
|  | 1607960_at | 0.16 | 0.21 | Cupredoxin superfamily protein |
|  | 1611332_at | 0.16 | 0.12 | Early nodulin-like protein 16 |
|  | 1614779_s_at | 0.18 | 0.1 | Remorin family protein |
|  | 1607347_at | 0.2 | 0.13 | RNA-binding (RRM/RBD/RNP motifs) family protein |
|  | 1618313_at | 0.2 | 0.12 | Heavy metal transport/detoxification superfamily protein |
|  | 1617863_at | 0.21 | 0.18 | Zinc knuckle (CCHC-type) family protein |
|  | 1621364_s_at | 0.21 | 0.12 | Membrane-associated progesterone binding protein 2 |
|  | 1607354_at | 0.23 | 0.1 | ACT domain repeat 3 |
|  | 1610009_at | 0.24 | 0.25 | Acyl-CoA-binding protein 6 |
|  | 1619899_at | 0.27 | 0.17 | Chloroplast RNA binding protein precursor |
|  | 1613050_at | 0.29 | 0.41 | RING zinc finger protein |
|  | 1614577_at | 0.29 | 0.22 | Nucleic acid-binding, OB-fold-like protein |
|  | 1613528_at | 0.29 | 0.13 | SKU5 similar 17 |
|  | 1618731_at | 0.3 | 0.32 | Chloroplast nucleoid DNA-binding protein-like protein |
|  | 1617311_at | 0.3 | 0.16 | 31-kDa RNA binding protein |
|  | 1612757_at | 0.31 | 0.38 | Non-phototropic hypocotyl |
|  | 1607049_s_at | 0.31 | 0.18 | Putative multiple inositol polyphosphate phosphatase |
|  | 1613521_at | 0.31 | 0.19 | Putative multiple inositol polyphosphate phosphatase |
|  | 1613036_at | 0.31 | 0.38 | GATA type zinc finger transcription factor family protein |
|  | 1608942_at | 0.33 | 0.39 | Putative C2H2-type zinc finger protein |
|  | 1617159_at | 0.35 | 0.39 | Translin-associated factor X |
|  | 1614225_at | 0.35 | 0.35 | COBRA-like extracellular glycosyl-phosphatidyl inositol-anchored protein family |
|  | 1612074_at | 0.35 | 0.1 | Similar to kinesin like protein |
|  | 1615445_at | 0.38 | 0.42 | Metallothionein 1a |
|  | 1616745_at | 0.42 | 0.43 | AT hook motif DNA-binding family protein |
|  | 1615304_at | 0.42 | 0.38 | GTP-binding family protein |
|  | 1617988_at | 0.43 | 0.26 | RNA-binding (RRM/RBD/RNP motifs) family protein |
|  | 1619852_s_at | 0.45 | 0.36 | Selenium-binding protein 1 |
|  | 1614493_at | 0.46 | 0.4 | 31 kDa ribonucleoprotein, chloroplast precursor |
|  | 1609155_at | 0.47 | 0.17 | SOUL heme-binding family protein |
|  | 1610241_at | 0.49 | 0.39 | RNA-binding (RRM/RBD/RNP motifs) family protein |
|  | 1612576_at | 0.5 | 0.35 | Single-strand DNA-binding |
| Transport regulation | 1607943_at | 0.03 | 0.01 | Putative aquaporin TIP3 |
|  | 1615829_s_at | 0.03 | 0.01 | Aquaporin |
|  | 1608175_at | 0.04 | 0.03 | Non-specific lipid-transfer protein |
|  | 1610982_at | 0.04 | 0.02 | Aquaporin PIP2 |
|  | 1612325_at | 0.05 | 0.05 | Cytochrome P450 |
|  | 1614916_at | 0.05 | 0.03 | Aquaporin PIP2 |
|  | 1617745_at | 0.06 | 0.04 | PVR3-like protein |
|  | 1622870_at | 0.06 | 0.05 | ABC-2 type transporter family protein |
|  | 1607561_at | 0.06 | 0.05 | ABC-2 type transporter family protein |
|  | 1615407_at | 0.07 | 0.07 | Copper transporter 2 |
|  | 1615295_at | 0.07 | 0.04 | Glycosylphosphatidylinositol-anchored lipid protein transfer 1 |
|  | 1616990_s_at | 0.08 | 0.03 | Bifunctional inhibitor |
|  | 1611159_at | 0.09 | 0.02 | Sulfate transporter |
|  | 1606724_at | 0.09 | 0.07 | Transmembrane amino acid transporter family protein |
|  | 1613045_at | 0.09 | 0.03 | Bifunctional inhibitor/lipid-transfer protein/seed storage 2S albumin superfamily protein |
|  | 1612244_s_at | 0.09 | 0.1 | Plasma membrane intrinsic protein 3 |
|  | 1620830_at | 0.09 | 0.07 | Pulvinus outward-rectifying channel for potassium SPOCK1 |
|  | 1608623_s_at | 0.11 | 0.27 | Amino acid permease 6 |
|  | 1606656_at | 0.11 | 0.02 | Bifunctional inhibitor/lipid-transfer protein/seed storage 2S albumin superfamily protein |
|  | 1611350_at | 0.12 | 0.11 | Bifunctional inhibitor/lipid-transfer protein/seed storage 2S albumin superfamily protein |
|  | 1622416_at | 0.12 | 0.1 | Non-specific lipid-transfer protein P5 |
|  | 1606834_s_at | 0.12 | 0.08 | Mitochondrial substrate carrier family protein |
|  | 1615141_at | 0.13 | 0.05 | Peptide transporter protein-like |
|  | 1607770_at | 0.13 | 0.02 | Bifunctional inhibitor/lipid-transfer protein/seed storage 2S albumin superfamily protein |
|  | 1612313_at | 0.14 | 0.3 | Amino acid permease 6 |
|  | 1614230_at | 0.14 | 0.1 | Non-specific lipid-transfer protein |
|  | 1622822_at | 0.14 | 0.15 | Gamma tonoplast intrinsic protein |
|  | 1621559_at | 0.14 | 0.15 | Multidrug resistance protein 11 |
|  | 1615669_at | 0.15 | 0.12 | Zinc transporter 5 precursor |
|  | 1620639_at | 0.15 | 0.05 | Inorganic phosphate transporter 2-1 |
|  | 1619918_at | 0.16 | 0.09 | Cationic amino acid transporter 7 |
|  | 1616456_at | 0.17 | 0.27 | Ammonium transporter 1;2 |
|  | 1618590_at | 0.17 | 0.09 | ABC-2 type transporter family protein |
|  | 1614222_at | 0.18 | 0.5 | Amino acid permease 6 |
|  | 1612852_at | 0.19 | 0.13 | Xanthine/uracil permease family protein |
|  | 1614363_at | 0.19 | 0.01 | Non-specific lipid-transfer protein |
|  | 1610366_at | 0.2 | 0.08 | Potassium transport 2/3 |
|  | 1609425_at | 0.21 | 0.29 | Heavy metal transport/detoxification superfamily protein |
|  | 1617847_at | 0.21 | 0.18 | Heavy metal transport/detoxification superfamily protein |
|  | 1608574_at | 0.21 | 0.25 | Similar to hypothetical protein At3g06130 |
|  | 1615359_at | 0.21 | 0.05 | Similar to P-glycoprotein |
|  | 1617061_s_at | 0.21 | 0.15 | Trigger factor type chaperone family protein |
|  | 1609903_at | 0.22 | 0.05 | Sulfate transporter 91 |
|  | 1613274_at | 0.22 | 0.13 | Glutamate receptor 3.6 |
|  | 1618261_at | 0.23 | 0.1 | Major facilitator superfamily protein |
|  | 1610527_at | 0.23 | 0.12 | Polyol/monosaccharide transporter 5 |
|  | 1615598_at | 0.24 | 0.16 | ATPase, coupled to transmembrane movement of substances |
|  | 1612744_at | 0.24 | 0.17 | White-brown complex homolog protein 11 |
|  | 1608408_at | 0.24 | 0.15 | ABC-2 type transporter family protein |
|  | 1610123_at | 0.25 | 0.17 | K+ uptake transporter 3 |
|  | 1612477_at | 0.25 | 0.29 | GroES-like zinc-binding alcohol dehydrogenase family protein |
|  | 1608952_at | 0.25 | 0.37 | Thioredoxin z |
|  | 1619295_at | 0.26 | 0.17 | Heavy metal transport/detoxification superfamily protein |
|  | 1615319_s_at | 0.26 | 0.44 | Major facilitator superfamily protein |
|  | 1614158_at | 0.26 | 0.32 | Major facilitator superfamily protein |
|  | 1606669_s_at | 0.26 | 0.2 | Aquaporin PIP2;4 |
|  | 1608571_s_at | 0.27 | 0.06 | Bifunctional inhibitor/lipid-transfer protein/seed storage 2S albumin superfamily protein |
|  | 1617032_s_at | 0.27 | 0.13 | Non-specific lipid-transfer protein |
|  | 1615415_s_at | 0.28 | 0.11 | Aquaporin PIP2;3 |
|  | 1616410_at | 0.28 | 0.2 | multidrug resistance-associated protein 6 |
|  | 1619421_at | 0.28 | 0.12 | ABC transporter family protein |
|  | 1622231_at | 0.29 | 0.25 | Lipid transfer protein |
|  | 1615808_s_at | 0.29 | 0.21 | Aquaporin PIP2;4 |
|  | 1617656_at | 0.3 | 0.44 | Outer plastid envelope protein 16-1 |
|  | 1608114_at | 0.3 | 0.24 | Inorganic H pyrophosphatase family protein |
|  | 1617522_a_at | 0.3 | 0.23 | Thioredoxin X |
|  | 1607144_at | 0.3 | 0.14 | Similar to plasma membrane H+ ATPase |
|  | 1614920_at | 0.31 | 0.28 | Potassium transporter 2 |
|  | 1617400_at | 0.31 | 0.06 | Sulfate transporter 3;1 |
|  | 1606611_at | 0.31 | 0.18 | Bifunctional inhibitor/lipid-transfer protein/seed storage 2S albumin superfamily protein |
|  | 1616429_at | 0.31 | 0.25 | Non-intrinsic ABC protein 9 |
|  | 1612302_at | 0.31 | 0.19 | Synaptobrevin-like protein |
|  | 1622315_at | 0.32 | 0.25 | Ttrigger factor type chaperone family protein |
|  | 1612329_at | 0.32 | 0.29 | Annexin 2 |
|  | 1615926_at | 0.33 | 0.09 | Tthioredoxin M-type 4 |
|  | 1619170_at | 0.34 | 0.23 | Major facilitator superfamily protein |
|  | 1622592_at | 0.34 | 0.45 | Divalent ion symporter |
|  | 1612739_at | 0.34 | 0.22 | Boron transporter |
|  | 1620256_at | 0.34 | 0.3 | Sucrose transporter |
|  | 1615893_at | 0.34 | 0.28 | Bifunctional inhibitor/lipid-transfer protein/seed storage 2S albumin superfamily protein |
|  | 1607452_at | 0.34 | 0.24 | Thioredoxin X |
|  | 1607517_at | 0.34 | 0.15 | WCRKC thioredoxin 1 |
|  | 1615210_at | 0.34 | 0.4 | Electron transfer flavoprotein alpha |
|  | 1615283_at | 0.34 | 0.22 | Quinone reductase family protein |
|  | 1621591_at | 0.34 | 0.34 | Cyclic nucleotide-gated cation channel 4 |
|  | 1616075_at | 0.35 | 0.4 | Mitochondrial substrate carrier family protein |
|  | 1613785_at | 0.35 | 0.32 | Putative ionotropic glutamate receptor homolog GLR4 |
|  | 1611423_s_at | 0.35 | 0.33 | Protein kinase superfamily protein |
|  | 1612609_at | 0.35 | 0.32 | Multidrug resistance-associated protein 6 |
|  | 1619056_at | 0.36 | 0.17 | Nuclear transport factor 2 |
|  | 1607658_at | 0.36 | 0.23 | Translocon at the inner envelope membrane of chloroplasts 55-II |
|  | 1614025_x_at | 0.36 | 0.41 | Aquaporin PIP1;3 |
|  | 1611167_at | 0.36 | 0.18 | General control non-repressible 5 |
|  | 1607916_at | 0.37 | 0.38 | Magnesium transporter 9 |
|  | 1617990_s_at | 0.37 | 0.23 | YELLOW STRIPE like 8 |
|  | 1619779_at | 0.37 | 0.31 | Emp24/gp25L/p24 family/GOLD family protein |
|  | 1622747_at | 0.37 | 0.27 | Bacterial sec-independent translocation protein mttA/Hcf106 |
|  | 1616831_at | 0.37 | 0.37 | Translocon at the inner envelope membrane of chloroplasts 110 |
|  | 1620245_at | 0.37 | 0.37 | Ferulic acid 5-hydroxylase 1 |
|  | 1615517_x_at | 0.37 | 0.47 | Aquaporin PIP1;3 |
|  | 1609870_at | 0.37 | 0.13 | Multidrug resistance-associated protein 9 |
|  | 1617359_at | 0.38 | 0.13 | Hydroxyproline-rich glycoprotein family protein |
|  | 1610016_at | 0.39 | 0.39 | Amino acid permease family protein |
|  | 1620373_at | 0.39 | 0.28 | thioredoxin X |
|  | 1607037_at | 0.39 | 0.33 | Shaker-like potassium channel |
|  | 1611528_at | 0.4 | 0.39 | Protein kinase superfamily protein |
|  | 1620420_at | 0.41 | 0.19 | 15-cis-zeta-carotene isomerase |
|  | 1616666_at | 0.41 | 0.38 | Mitochondrial substrate carrier family protein |
|  | 1619157_at | 0.41 | 0.18 | Inward rectifying potassium channel n |
|  | 1620972_at | 0.42 | 0.35 | Xanthine/uracil permease family protein |
|  | 1619012_s_at | 0.43 | 0.49 | C-terminal cysteine residue is changed to a serine 1 |
|  | 1617711_s_at | 0.44 | 0.22 | Ferritin |
|  | 1608833_at | 0.44 | 0.32 | Nucleotide-sugar transporter family protein |
|  | 1614704_at | 0.45 | 0.41 | Mitochondrial substrate carrier family protein |
|  | 1620735_at | 0.46 | 0.26 | FAD/NAD(P)-binding oxidoreductase family protein |
|  | 1622021_at | 0.46 | 0.35 | Sodium hydrogen exchanger 2 |
|  | 1615547_at | 0.46 | 0.35 | Expressed protein localized to the inner membrane of the chloroplast |
|  | 1618747_at | 0.48 | 0.47 | Mercaptopyruvate sulfurtransferase 1 |
|  | 1620378_at | 0.48 | 0.16 | Major facilitator superfamily protein |
|  | 1621821_s_at | 0.48 | 0.47 | Hydrogen-transporting ATP synthase |
|  | 1622157_at | 0.5 | 0.22 | Glucose-6-phosphate/phosphate translocator-related |
|  | 1609626_at | 0.5 | 0.4 | Dicarboxylate transporter 1 |
| Signal transduction | 1607664_at | 0.05 | 0.04 | Cytochrome b |
|  | 1619795_at | 0.07 | 0.02 | Similar to leucine-rich repeat family protein |
|  | 1607946_at | 0.1 | 0.13 | Concanavalin A-like lectin protein kinase family protein |
|  | 1609996_at | 0.1 | 0.12 | Leucine-rich repeat protein kinase family protein |
|  | 1611503_s_at | 0.11 | 0.29 | CBL-interacting protein kinase 7 |
|  | 1611337_at | 0.11 | 0.06 | Rho GTPase activating protein with PAK-box |
|  | 1618956_at | 0.11 | 0.09 | Transducin/WD40 repeat-like superfamily protein |
|  | 1611555_at | 0.11 | 0.37 | EF hand calcium-binding protein family |
|  | 1620472_at | 0.11 | 0.09 | Protein kinase superfamily protein |
|  | 1614358_at | 0.11 | 0.12 | Transmembrane kinase-like 1 |
|  | 1607524_at | 0.12 | 0.12 | Leucine-rich receptor-like protein kinase family protein |
|  | 1618441_at | 0.14 | 0.36 | Leucine-rich receptor-like protein kinase family protein |
|  | 1621455_at | 0.14 | 0.1 | Leucine-rich repeat protein kinase family protein |
|  | 1615962_at | 0.17 | 0.1 | Rac-like GTP-binding protein ARAC3 |
|  | 1616611_at | 0.17 | 0.09 | Protein kinase superfamily protein |
|  | 1612893_at | 0.18 | 0.18 | Translocon at the outer envelope membrane of chloroplasts 159 |
|  | 1612729_at | 0.18 | 0.09 | Leucine-rich repeat receptor-like protein kinase family protein |
|  | 1610279_s_at | 0.19 | 0.15 | Lysophospholipase-like protein |
|  | 1614131_at | 0.19 | 0.12 | Sec14p-like phosphatidylinositol transfer family protein |
|  | 1616710_at | 0.19 | 0.14 | Leucine-rich repeat protein kinase family protein |
|  | 1607099_s_at | 0.2 | 0.21 | Leucine-rich repeat protein kinase family protein |
|  | 1619654_at | 0.21 | 0.19 | Lysophospholipase-like protein |
|  | 1617238_at | 0.21 | 0.35 | Leucine-rich repeat protein kinase family protein |
|  | 1611248_at | 0.22 | 0.19 | Transducin/WD40 repeat-like superfamily protein |
|  | 1608969_at | 0.24 | 0.12 | Putative receptor protein kinase TMK1 precursor |
|  | 1608084_x_at | 0.25 | 0.48 | CBL-interacting protein kinase 9 |
|  | 1611562_at | 0.25 | 0.16 | LRR receptor-like kinase |
|  | 1610815_at | 0.25 | 0.23 | Leucine-rich repeat protein kinase family protein |
|  | 1622512_at | 0.26 | 0.14 | Rho guanyl-nucleotide exchange factor 1 |
|  | 1612956_at | 0.27 | 0.38 | NSP-interacting kinase 2 |
|  | 1614666_s_at | 0.27 | 0.46 | RAB homolog 1 |
|  | 1613767_at | 0.27 | 0.27 | Similarity to calmodulin-binding protein |
|  | 1607756_s_at | 0.27 | 0.21 | Phospholipase C 2 |
|  | 1610447_at | 0.28 | 0.23 | Phospholipase C 2 |
|  | 1622444_at | 0.28 | 0.18 | Leucine-rich repeat |
|  | 1606589_at | 0.29 | 0.23 | IQ-domain 2 |
|  | 1611223_at | 0.29 | 0.22 | Calcium-binding EF-hand family protein |
|  | 1607639_s_at | 0.29 | 0.22 | Receptor kinase 3 |
|  | 1607610_at | 0.3 | 0.19 | S-locus lectin protein kinase family protein |
|  | 1613012_at | 0.31 | 0.2 | Protein kinase family protein |
|  | 1608204_at | 0.31 | 0.16 | RHO-related protein from plants 9 |
|  | 1619119_at | 0.31 | 0.1 | Calcium sensing receptor |
|  | 1622305_at | 0.31 | 0.24 | Phospholipase D delta |
|  | 1622757_at | 0.31 | 0.45 | Phosphatase family protein |
|  | 1620367_at | 0.31 | 0.34 | Leucine-rich repeat protein kinase family protein |
|  | 1621263_at | 0.32 | 0.29 | U-box domain-containing protein kinase family protein |
|  | 1610530_at | 0.32 | 0.16 | Protein kinase superfamily protein |
|  | 1608590_at | 0.33 | 0.18 | GTP cyclohydrolase II/3,4-dihydroxy-2-butanone 4-phosphate synthase |
|  | 1619249_at | 0.33 | 0.26 | Similar to Rac-like GTP-binding protein RAC2 |
|  | 1617175_at | 0.33 | 0.18 | 1-phosphatidylinositol-4-phosphate 5-kinases |
|  | 1620938_at | 0.34 | 0.34 | Extra-large G-protein |
|  | 1609354_at | 0.34 | 0.45 | P-loop containing nucleoside triphosphate hydrolases superfamily protein |
|  | 1619479_a_at | 0.34 | 0.28 | Putative 7-transmembrane G-protein-coupled receptor |
|  | 1619722_at | 0.35 | 0.26 | Mitogen-activated protein kinase 16 |
|  | 1611115_s_at | 0.37 | 0.09 | Ras-related small GTP-binding family protein |
|  | 1606720_at | 0.37 | 0.3 | RAC-like GTP binding protein 5 |
|  | 1618419_at | 0.37 | 0.13 | Calcium sensing receptor |
|  | 1616098_at | 0.38 | 0.11 | Ras-related small GTP-binding family protein |
|  | 1610229_at | 0.39 | 0.28 | IQ-domain 12 |
|  | 1610085_at | 0.4 | 0.33 | Leucine-rich repeat protein kinase family protein |
|  | 1622441_at | 0.41 | 0.47 | CDPK-related kinase 1 |
|  | 1612021_at | 0.42 | 0.4 | Leucine-rich receptor-like protein kinase family protein |
|  | 1614027_s_at | 0.44 | 0.42 | NAD kinase 2 |
|  | 1606658_at | 0.44 | 0.36 | P-loop containing nucleoside triphosphate hydrolases superfamily protein |
|  | 1610384_s_at | 0.44 | 0.26 | Calcium-dependent lipid-binding (CaLB domain) family protein |
|  | 1615231_at | 0.44 | 0.46 | RAB GTPase homolog A5E |
|  | 1610925_at | 0.45 | 0.49 | U-box domain-containing protein kinase family protein |
|  | 1618505_at | 0.46 | 0.26 | Protein kinase superfamily protein |
|  | 1616648_at | 0.46 | 0.32 | Ras-related small GTP-binding family protein |
|  | 1607809_s_at | 0.46 | 0.26 | Inositol-pentakisphosphate 2-kinase family protein |
|  | 1613879_at | 0.47 | 0.12 | Thylakoid-associated phosphatase 38 |
|  | 1613429_s_at | 0.47 | 0.26 | Myo-inositol-1-phosphate synthase 2 |
|  | 1617008_at | 0.47 | 0.5 | Putative receptor-like protein kinase 3 |
|  | 1613808_at | 0.5 | 0.36 | Protein phosphatase 2C family protein |
|  | 1607490_at | 0.5 | 0.42 | Rho GTPase activating protein with PAK-box |
|  | 1607952_at | 0.5 | 0.29 | HAESA-like 1 |
| Cell rescue | 1613471_at | 0.05 | 0.02 | CAP (Cysteine-rich secretory proteins, Antigen 5, and Pathogenesis-related 1 protein) superfamily protein |
|  | 1619667_at | 0.07 | 0.06 | Leucine-rich repeat (LRR) family protein |
|  | 1607967_at | 0.07 | 0.04 | Alcohol dehydrogenase 7 |
|  | 1618920_at | 0.07 | 0.05 | Peroxidase superfamily protein |
|  | 1608586_at | 0.08 | 0.04 | Peroxidase superfamily protein |
|  | 1609383_at | 0.09 | 0.02 | Predicted to encode a PR (pathogenesis-related) protein |
|  | 1612037_s_at | 0.09 | 0.02 | Predicted to encode a PR (pathogenesis-related) protein |
|  | 1614086_at | 0.1 | 0.18 | Alcohol dehydrogenase |
|  | 1621611_at | 0.12 | 0.11 | Drought-responsive family protein |
|  | 1621901_at | 0.12 | 0.06 | Disease resistance-responsive (dirigent-like protein) family protein |
|  | 1610299_at | 0.12 | 0.09 | MLP-like protein 423 |
|  | 1620624_at | 0.13 | 0.05 | UDP-glucosyltransferase 75B1 |
|  | 1618164_at | 0.13 | 0.11 | cytochrome P450, family 96, subfamily A, polypeptide 1 |
|  | 1612707_at | 0.14 | 0.07 | Superoxide dismutase |
|  | 1606772_at | 0.15 | 0.2 | Putative salt-inducible protein |
|  | 1616426_at | 0.15 | 0.06 | RD22-like protein |
|  | 1610481_at | 0.15 | 0.06 | Late embryogenesis abundant protein, group 2 |
|  | 1613132_s_at | 0.15 | 0.07 | Peroxidase superfamily protein |
|  | 1615444_at | 0.16 | 0.09 | Dehydration-induced protein RD22-like protein |
|  | 1613067_at | 0.17 | 0.06 | BURP domain-containing protein |
|  | 1613825_at | 0.18 | 0.11 | Stress regulated protein |
|  | 1610756_at | 0.18 | 0.17 | Leucine-rich repeat (LRR) family protein |
|  | 1615821_at | 0.18 | 0.07 | Chaperone DnaJ-domain superfamily protein |
|  | 1621587_at | 0.2 | 0.34 | APS reductase 1 |
|  | 1616877_at | 0.2 | 0.12 | OBP3-responsive gene 4 |
|  | 1619111_s_at | 0.21 | 0.1 | Disease resistance-responsive (dirigent-like protein) family protein |
|  | 1620522_at | 0.22 | 0.09 | Salt tolerance homologue |
|  | 1616711_at | 0.22 | 0.21 | Disease resistance-responsive (dirigent-like protein) family protein |
|  | 1620738_at | 0.23 | 0.12 | Thioredoxin superfamily protein |
|  | 1622001_at | 0.25 | 0.36 | Unknown seed protein like 1 |
|  | 1613844_at | 0.25 | 0.21 | Alpha/beta-Hydrolases superfamily protein |
|  | 1612444_at | 0.26 | 0.16 | MLP-like protein 28 |
|  | 1608003_s_at | 0.26 | 0.24 | Cytochrome P450, family 72, subfamily A, polypeptide 9 |
|  | 1608541_s_at | 0.27 | 0.11 | NAD(P)-binding Rossmann-fold superfamily protein |
|  | 1615250_at | 0.29 | 0.08 | Eukaryotic aspartyl protease family protein |
|  | 1612460_s_at | 0.29 | 0.41 | CAX-interacting protein 2 |
|  | 1608433_at | 0.29 | 0.11 | Thioredoxin superfamily protein |
|  | 1614204_at | 0.3 | 0.09 | Thioredoxin superfamily protein |
|  | 1621501_at | 0.3 | 0.33 | P67-like superoxide-generating NADPH oxidase |
|  | 1610553_at | 0.31 | 0.15 | Avr9/Cf-9 rapidly elicited protein 194 |
|  | 1607783_at | 0.31 | 0.29 | Transporter |
|  | 1613998_at | 0.31 | 0.14 | Phytochelatin synthetase-like protein |
|  | 1620400_at | 0.34 | 0.49 | Pathogenesis-related gene 1 |
|  | 1621583_at | 0.34 | 0.23 | Chitinase-like protein 2 |
|  | 1618599_at | 0.37 | 0.33 | Superoxide dismutase |
|  | 1615776_at | 0.38 | 0.33 | Putative ripening-related protein |
|  | 1609029_at | 0.38 | 0.28 | Disease resistance-responsive (dirigent-like protein) family protein |
|  | 1610521_at | 0.39 | 0.42 | MLP-like protein 28 |
|  | 1609165_at | 0.42 | 0.08 | Alpha/beta-Hydrolases superfamily protein |
|  | 1609269_at | 0.42 | 0.39 | NAD(P)-binding Rossmann-fold superfamily protein |
|  | 1614324_at | 0.42 | 0.25 | HYS1-related |
|  | 1609202_at | 0.42 | 0.28 | Glutathione S-transferase tau 7 |
|  | 1619419_at | 0.42 | 0.38 | Glutathione S-transferase family protein |
|  | 1608907_s_at | 0.43 | 0.16 | Galactinol synthase 4 |
|  | 1608019_s_at | 0.43 | 0.24 | Chloroplastic drought-induced stress protein of 32 kD |
|  | 1610772_at | 0.44 | 0.2 | Low temperature and salt responsive protein family |
|  | 1617173_s_at | 0.44 | 0.09 | Chlorophyll A-B binding family protein |
|  | 1621213_at | 0.44 | 0.36 | Glycosyl hydrolase family protein with chitinase insertion domain |
|  | 1617661_at | 0.45 | 0.48 | Putative Machado-Joseph disease gene product ataxin-3 |
|  | 1609873_at | 0.46 | 0.21 | Glyoxalase/Bleomycin resistance protein/Dioxygenase superfamily protein |
|  | 1616888_at | 0.46 | 0.35 | Similar to lactoylglutathione lyase, putative / glyoxalase I |
|  | 1620867_at | 0.46 | 0.37 | HXXXD-type acyl-transferase family protein |
|  | 1609901_at | 0.47 | 0.35 | Thioredoxin superfamily protein |
|  | 1619210_at | 0.47 | 0.29 | Superoxide dismutase [Cu-Zn], chloroplast precursor |
|  | 1620163_x_at | 0.48 | 0.29 | Low temperature and salt responsive protein family |
|  | 1617035_s_at | 0.48 | 0.21 | Galactinol synthase 4 |
|  | 1617515_at | 0.48 | 0.25 | L-ascorbate peroxidase |
|  | 1622739_at | 0.48 | 0.48 | Peroxidase superfamily protein |
|  | 1622266_x_at | 0.49 | 0.11 | Chlorophyll A-B binding family protein |
|  | 1608089_at | 0.5 | 0.42 | Glutathione peroxidase |
| Interaction with cellular environment | 1616731_at | 0.09 | 0.02 | CCR-like |
|  | 1612035_at | 0.39 | 0.25 | Putative COP-1 interacting protein 7 |
|  | 1622167_at | 0.39 | 0.47 | Galactose oxidase/kelch repeat superfamily protein |
| Plant / fungal specific systemic sensing and response | 1606566_at | 0.01 | 0.01 | SAUR-like auxin-responsive protein family |
|  | 1613054_at | 0.01 | 0.01 | SAUR-like auxin-responsive protein family |
|  | 1606517_at | 0.05 | 0.02 | Pathogenesis-related thaumatin superfamily protein |
|  | 1607897_at | 0.05 | 0.03 | Gibberellin-regulated family protein |
|  | 1612160_at | 0.07 | 0.03 | Pathogenesis-related thaumatin superfamily protein |
|  | 1619658_at | 0.07 | 0.11 | Auxin-responsive GH3 family protein |
|  | 1616717_at | 0.07 | 0.09 | AUX/IAA transcriptional regulator family protein |
|  | 1608099_at | 0.07 | 0.09 | Brassinosteroid-6-oxidase |
|  | 1613468_at | 0.09 | 0.05 | Indoleacetic acid-induced protein 16 |
|  | 1616961_at | 0.09 | 0.06 | Gibberellin oxidase-like protein |
|  | 1621818_at | 0.1 | 0.07 | BURP domain-containing protein |
|  | 1621946_at | 0.11 | 0.21 | PIN1-like auxin transport protein |
|  | 1618245_at | 0.11 | 0.08 | Stress responsive A/B Barrel Domain |
|  | 1609435_at | 0.13 | 0.15 | SCUTL1 |
|  | 1619751_at | 0.13 | 0.04 | AUX/IAA transcriptional regulator family protein |
|  | 1612180_at | 0.16 | 0.1 | Auxin response factor 4 |
|  | 1610607_at | 0.16 | 0.13 | Gibberellin-regulated protein |
|  | 1622860_at | 0.16 | 0.07 | Putative lysine decarboxylase family protein |
|  | 1613857_at | 0.17 | 0.16 | Phytochrome-associated protein 1 |
|  | 1608070_at | 0.17 | 0.25 | F-box containing protein TIR1 |
|  | 1611491_at | 0.17 | 0.18 | Auxin efflux carrier family protein |
|  | 1616104_at | 0.18 | 0.18 | SAUR-like auxin-responsive protein family |
|  | 1620224_at | 0.18 | 0.13 | Auxin-responsive family protein |
|  | 1609099_at | 0.18 | 0.1 | Cytochrome P450 superfamily protein |
|  | 1611390_a_at | 0.19 | 0.13 | Indole-3-acetic acid 7 |
|  | 1615985_at | 0.19 | 0.07 | AUX/IAA transcriptional regulator family protein |
|  | 1620512_at | 0.2 | 0.16 | Phytochrome-associated protein 2 |
|  | 1619741_at | 0.2 | 0.12 | Indoleacetic acid-induced protein 16 |
|  | 1617541_s_at | 0.21 | 0.08 | Beta-carotene hydroxylase 2 |
|  | 1618871_at | 0.22 | 0.26 | Putative thaumatin-like protein |
|  | 1616581_at | 0.22 | 0.14 | Transcription factor BHLH9-like protein |
|  | 1611468_at | 0.24 | 0.24 | Phytochrome B |
|  | 1617881_at | 0.25 | 0.22 | Gibberellin-regulated family protein |
|  | 1611657_at | 0.28 | 0.18 | Outer arm dynein light chain 1 protein |
|  | 1611489_at | 0.28 | 0.22 | Indole-3-acetic acid 7 |
|  | 1606686_at | 0.28 | 0.22 | NAD(P)-binding Rossmann-fold superfamily protein |
|  | 1608814_at | 0.3 | 0.12 | Beta-hydroxylase 1 |
|  | 1621497_at | 0.32 | 0.29 | Protein phosphatase 2C (AtP2C-HA) |
|  | 1606727_at | 0.32 | 0.43 | Phytochrome F |
|  | 1612994_at | 0.33 | 0.3 | HVA22 homologue A |
|  | 1616015_at | 0.34 | 0.3 | Similar to auxin response factor 1 |
|  | 1606777_s_at | 0.34 | 0.31 | DELLA protein GAI1 |
|  | 1615321_at | 0.35 | 0.3 | Auxin-responsive protein IAA13 |
|  | 1606941_at | 0.35 | 0.33 | Ethylene overproducer-like 1 |
|  | 1610455_at | 0.36 | 0.08 | Nine-cis-epoxycarotenoid dioxygenase 4 |
|  | 1615754_at | 0.37 | 0.28 | O-fucosyltransferase family protein |
|  | 1615888_at | 0.39 | 0.15 | Non-photochemical quenching 1 |
|  | 1617604_at | 0.39 | 0.22 | SPA1-related 4 |
|  | 1618006_at | 0.4 | 0.35 | Indole-3-acetic acid inducible 9 |
|  | 1615645_at | 0.4 | 0.39 | Peptidase M20/M25/M40 family protein |
|  | 1621228_at | 0.4 | 0.27 | Similar to DELLA protein |
|  | 1609027_at | 0.4 | 0.28 | Aldehyde dehydrogenase 3F1 |
|  | 1622268_at | 0.4 | 0.33 | SPA1-related 2 |
|  | 1608474_at | 0.41 | 0.2 | Shaggy-like kinase 13 |
|  | 1620078_at | 0.42 | 0.28 | Auxin responsive SAUR protein |
|  | 1611998_at | 0.42 | 0.16 | Violaxanthin de-epoxidase |
|  | 1619068_at | 0.43 | 0.36 | Xyloglucan endotransglucosylase/hydrolase 15 |
|  | 1615487_at | 0.46 | 0.47 | Cysteine proteinases superfamily protein |
|  | 1617680_at | 0.46 | 0.48 | Phytochrome-associated protein 1 |
|  | 1618301_at | 0.46 | 0.36 | Lanthionine synthetase C-like |
|  | 1622471_at | 0.47 | 0.39 | Signal transduction histidine kinase, hybrid-type, ethylene sensor |
|  | 1612564_at | 0.48 | 0.33 | Similar to auxin response factor 8 |
|  | 1621754_at | 0.48 | 0.31 | Indoleacetic acid-induced protein 16 |
|  | 1607708_at | 0.49 | 0.41 | Pathogenesis-related thaumatin superfamily protein |
|  | 1612060_at | 0.49 | 0.44 | Auxin transporter protein 1 |
|  | 1612940_at | 0.49 | 0.46 | Cysteine proteinases superfamily protein |
| Transposable elements | 1610659_at | 0.1 | 0.19 | Zinc finger, RING-type; RINGv |
|  | 1607839_at | 0.12 | 0.12 | Retroelement pol polyprotein-like |
|  | 1620151_at | 0.17 | 0.27 | Putative NPH3 family protein |
|  | 1613257_at | 0.37 | 0.24 | Tetratricopeptide repeat (TPR)-containing protein |
|  | 1617817_at | 0.43 | 0.45 | Similarity to retroelement pol polyprotein |
| Cell fate | 1620840_at | 0.03 | 0.03 | Alpha-expansin precursor |
|  | 1608074_s_at | 0.03 | 0.02 | Expansin |
|  | 1619010_s_at | 0.03 | 0.02 | Expansin |
|  | 1619082_at | 0.05 | 0.06 | Expansin A1 |
|  | 1612030_at | 0.09 | 0.12 | Epicotyl-specific tissue protein |
|  | 1617739_at | 0.1 | 0.21 | Xyloglucan endotransglucosylase/hydrolase 5 |
|  | 1622121_at | 0.1 | 0.09 | Glycosyl hydrolase 9B8 |
|  | 1614426_at | 0.13 | 0.04 | Xyloglucan endotransglucosylase/hydrolase 15 |
|  | 1612253_at | 0.18 | 0.27 | Alpha-expansin 1 |
|  | 1608191_at | 0.2 | 0.11 | Expansin A6 |
|  | 1620608_s_at | 0.2 | 0.09 | Thiol protease aleurain-like precursor |
|  | 1616312_at | 0.2 | 0.39 | Putative phytosulfokine peptide precursor |
|  | 1617150_at | 0.21 | 0.19 | Expansin |
|  | 1609909_s_at | 0.21 | 0.09 | Xyloglucan endotransglucosylase/hydrolase 15 |
|  | 1620096_at | 0.29 | 0.19 | Xyloglucan endotransglucosylase/hydrolase 9 |
|  | 1615533_s_at | 0.31 | 0.25 | Xyloglucan endotransglucosylase/hydrolase 7 |
|  | 1622108_at | 0.34 | 0.15 | Putative monocopper oxidase precursor |
|  | 1619881_at | 0.42 | 0.05 | Thiol protease aleurain-like precursor |
| Development | 1611086_at | 0.12 | 0.07 | HXXXD-type acyl-transferase family protein |
|  | 1614189_at | 0.17 | 0.13 | Flowering locus T-like protein |
|  | 1615867_at | 0.42 | 0.39 | LisH dimerisation motif;WD40/YVTN repeat-like-containing domain |
|  | 1618023_at | 0.49 | 0.35 | Tetraspanin family protein |
| Biogenesis of cellular component | 1608528_at | 0.09 | 0.06 | Photosystem II family protein |
|  | 1618121_at | 0.11 | 0.07 | Extensin protein-like |
|  | 1612981_at | 0.12 | 0.1 | Tubulin beta 8 |
|  | 1607341_at | 0.13 | 0.05 | GDSL-like Lipase/Acylhydrolase superfamily protein |
|  | 1620864_at | 0.13 | 0.11 | Kinase interacting (KIP1-like) family protein |
|  | 1620976_at | 0.14 | 0.12 | Proline-rich protein |
|  | 1607837_at | 0.15 | 0.09 | Fasciclin-like arabinogalactan protein 8 precursor |
|  | 1615089_at | 0.15 | 0.1 | Tubulin beta-7 chain |
|  | 1607001_at | 0.16 | 0.08 | Tubulin beta-1 chain |
|  | 1613467_at | 0.18 | 0.12 | Pollen Ole e 1 allergen and extensin family protein |
|  | 1613441_s_at | 0.18 | 0.09 | Similar to hydroxyproline-rich glycoprotein |
|  | 1611168_at | 0.18 | 0.11 | Tubulin/FtsZ family protein |
|  | 1612541_at | 0.18 | 0.01 | Peroxin 11c |
|  | 1616815_at | 0.2 | 0.14 | Beta-6 tubulin |
|  | 1615949_at | 0.22 | 0.06 | Protocadherin 15b |
|  | 1613677_at | 0.23 | 0.21 | FASCICLIN-like arabinoogalactan 7 |
|  | 1614049_at | 0.23 | 0.16 | Putative arabinogalactan-protein AGP14 |
|  | 1620583_s_at | 0.23 | 0.21 | Arabinogalactan-protein |
|  | 1622481_x_at | 0.25 | 0.25 | Proline-rich cell wall protein |
|  | 1618093_a_at | 0.25 | 0.17 | Similar to hypothetical protein At3g11700 |
|  | 1622843_s_at | 0.25 | 0.19 | Putative arabinogalactan-protein AGP14 |
|  | 1618889_at | 0.25 | 0.1 | Hydroxyproline-rich glycoprotein DZ-HRGP precursor |
|  | 1619969_at | 0.25 | 0.22 | Tubulin beta 8 |
|  | 1615201_at | 0.27 | 0.08 | Bifunctional inhibitor/lipid-transfer protein/seed storage 2S albumin superfamily protein |
|  | 1609349_s_at | 0.27 | 0.27 | Proline-rich cell wall protein |
|  | 1607162_s_at | 0.28 | 0.11 | Putative proline-rich protein |
|  | 1622641_at | 0.28 | 0.29 | VMP4 protein |
|  | 1617023_at | 0.29 | 0.22 | Fasciclin-like arabinogalactan protein 1 precursor |
|  | 1614517_x_at | 0.29 | 0.3 | Proline-rich cell wall protein |
|  | 1608807_at | 0.29 | 0.1 | RHO guanyl-nucleotide exchange factor 11 |
|  | 1607155_at | 0.29 | 0.09 | Tubulin alpha-6 chain |
|  | 1608133_at | 0.3 | 0.15 | Actin 3 |
|  | 1607541_at | 0.31 | 0.24 | TonB, C-terminal domain |
|  | 1607325_at | 0.32 | 0.17 | NFU domain protein 3 |
|  | 1619695_at | 0.33 | 0.16 | 2S albumin |
|  | 1606427_at | 0.36 | 0.45 | Putative glycine rich protein |
|  | 1619655_at | 0.37 | 0.18 | Putative membrane protein precursor |
|  | 1614807_s_at | 0.38 | 0.2 | Alpha-1 tubulin |
|  | 1622787_at | 0.4 | 0.33 | End binding protein 1B |
|  | 1616523_s_at | 0.41 | 0.27 | Arabinogalactan protein 41 |
|  | 1619789_at | 0.43 | 0.37 | Putative membrane protein |
|  | 1614951_at | 0.46 | 0.24 | EXORDIUM like 3 |
|  | 1609433_s_at | 0.46 | 0.45 | Translationally controlled tumor protein |
|  | 1611137_at | 0.46 | 0.32 | NFU domain protein 3 |
|  | 1618413_at | 0.47 | 0.28 | Tubulin alpha-2 chain |
|  | 1612484_at | 0.49 | 0.22 | Kinesin like protein for actin based chloroplast movement 2 |
|  | 1617526_s_at | 0.5 | 0.37 | Pherophorin-C2 protein precursor |
