# Supplementary material for: Transcriptomic Analysis of Grape (Vitis vinifera L.) Leaves after Exposure to Ultraviolet C Irradiation
Source: PLoS One. 2014 Dec 2;9(12):e113772. doi: 10.1371/journal.pone.0113772 (PMC4252036; doi:10.1371/journal.pone.0113772)
Supplement: Additional file S5 — Probe sets specifically up- and down-regulated at 6 h after exposure to UV-C treatment. (DOCX) [file pone.0113772.s005.docx]

**Additional file S5** Unique genes up- or down-regulated at 6 h after UV-C treatment

| Category | Probe set ID | Fold change | Gene name description |
| --- | --- | --- | --- |
| Metabolism | 1615604_at | 5.86 | Terpene synthase 21 |
|  | 1621838_at | 4.81 | Carboxyesterase 18 |
|  | 1621378_at | 3.01 | Ribose-phosphate pyrophosphokinase |
|  | 1621051_at | 2.56 | UDP-Glycosyltransferase superfamily protein |
|  | 1619220_at | 2.34 | Spermidine synthase 1 |
|  | 1606915_at | 2.28 | Phosphocholine cytidylyltransferase |
|  | 1622503_at | 2.25 | Elicitor-activated gene 3-1 |
|  | 1611011_at | 2.24 | 2-oxoglutarate (2OG) and Fe(II)-dependent oxygenase superfamily protein |
|  | 1609854_at | 2.18 | Elicitor-activated gene 3-1 |
|  | 1620149_at | 2.17 | Fatty acid/sphingolipid desaturase |
|  | 1621918_at | 2.16 | Benzoyl coenzyme A: benzyl alcohol benzoyl transferase |
|  | 1613717_a_at | 2.13 | Auxin-responsive family protein |
|  | 1620650_s_at | 2.09 | Elicitor-activated gene 3-1 |
|  | 1612050_at | 2.08 | Chitinase A |
|  | 1621728_at | 2.03 | Quinate hydroxycinnamoyl transferase |
|  | 1619913_at | 2.03 | Auxin-responsive family protein |
|  | 1621343_at | 2.02 | Indole-3-butyric acid response 10 |
|  | 1614681_at | 2.01 | UDP-D-apiose/UDP-D-xylose synthase 1 |
|  | 1617719_at | 2 | Reversibly glycosylated polypeptide 3 |
|  | 1606799_s_at | 0.49 | Malate dehydrogenase |
|  | 1610169_at | 0.48 | nicotinamidase 1 |
|  | 1620457_at | 0.48 | Uridine 5'-monophosphate synthase / UMP synthase (PYRE-F) (UMPS) |
|  | 1610035_x_at | 0.47 | Malate dehydrogenase |
|  | 1618606_at | 0.46 | 3-ketoacyl-acyl carrier protein synthase I |
|  | 1607607_s_at | 0.46 | Flavanone 3-hydroxylase |
|  | 1620325_at | 0.44 | Homocysteine methyltransferase 2 |
|  | 1611362_at | 0.44 | HAD superfamily, subfamily IIIB acid phosphatase |
|  | 1620997_at | 0.43 | L-galactose dehydrogenase |
|  | 1621719_at | 0.42 | 2-oxoacid dehydrogenases acyltransferase family protein |
|  | 1622252_at | 0.41 | L-idonate 5-dehydrogenase |
|  | 1612571_at | 0.41 | UDP-glucosyl transferase 85A2 |
|  | 1620308_at | 0.41 | 3-oxo-5-alpha-steroid 4-dehydrogenase family protein |
|  | 1608583_at | 0.39 | Aldehyde dehydrogenase 2B7 |
|  | 1618485_at | 0.39 | Purple acid phosphatase 1 |
|  | 1615374_at | 0.39 | Sucrose phosphate synthase 1F |
|  | 1612918_at | 0.38 | L-idonate dehydrogenase |
|  | 1615579_at | 0.37 | Acyl-[acyl-carrier protein] desaturase |
|  | 1611804_at | 0.35 | UDP-Glycosyltransferase superfamily protein |
|  | 1613627_at | 0.35 | Gamma-aminobutyrate transaminase subunit precursor |
|  | 1622079_at | 0.31 | Biotin carboxyl carrier protein of acetyl-CoA carboxylase, chloroplast precursor |
|  | 1616437_at | 0.3 | NAD(P)-binding Rossmann-fold superfamily protein |
|  | 1616615_at | 0.27 | Dihydrodipicolinate reductase |
|  | 1612185_at | 0.26 | Glycosyl hydrolase superfamily protein |
|  | 1611613_at | 0.13 | Glycosyl hydrolases family 32 protein |
| Energy | 1619717_at | 2.05 | Protochlorophyllide oxidoreductase A |
|  | 1610336_at | 0.42 | Pyruvate dehydrogenase kinase |
|  | 1609301_at | 0.4 | Aldolase-type TIM barrel family protein |
|  | 1611072_at | 0.34 | Glycine decarboxylase complex H-protein |
|  | 1613182_at | 0.22 | Pyruvate dehydrogenase kinase |
| Storage protein  cell cyecle and DNA processing | 1619225_s_at | 2.56 | DNAJ homologue 3 |
|  | 1607336_at | 0.5 | Actin depolymerizing factor 6 |
|  | 1617887_at | 0.48 | Histone H2B |
|  | 1614219_at | 0.48 | Histone superfamily protein |
|  | 1607931_s_at | 0.47 | Histone H1 |
|  | 1610051_at | 0.46 | ATPase family associated with various cellular activities (AAA) |
|  | 1610007_at | 0.41 | Histone H1-3 |
|  | 1617566_at | 0.4 | S-adenosyl-L-methionine-dependent methyltransferases superfamily protein |
|  | 1622440_at | 0.33 | Histone H3.2 |
| Transcription | 1612636_at | 6.75 | Acetyl-CoA acyltransferase |
|  | 1617411_at | 4.51 | Basic helix-loop-helix (bHLH) DNA-binding superfamily protein |
|  | 1609171_at | 3.51 | Hypothetical protein |
|  | 1615012_s_at | 3.14 | GRAS family transcription factor |
|  | 1617671_s_at | 3.01 | Erf domain protein 9 |
|  | 1615596_s_at | 2.96 | A20/AN1-like zinc finger family protein |
|  | 1620817_at | 2.87 | GRAS family transcription factor |
|  | 1613186_at | 2.84 | A20/AN1-like zinc finger family protein |
|  | 1616057_s_at | 2.7 | BZip transcription factor |
|  | 1619433_at | 2.66 | KNOTTED1-like homeobox gene 3 |
|  | 1613318_at | 2.64 | MYB family transcription factor |
|  | 1619386_at | 2.57 | MYB family transcription factor |
|  | 1622333_at | 2.51 | WRKY DNA-binding protein 23 |
|  | 1620999_at | 2.47 | Tesmin/TSO1-like |
|  | 1609138_at | 2.38 | C2H2 type Zn finger-containing protein |
|  | 1615625_at | 2.33 | KNOTTED1-like homeobox gene 3 |
|  | 1620607_s_at | 2.31 | B-box type zinc finger protein with CCT domain |
|  | 1608936_at | 2.19 | Basic helix-loop-helix (bHLH) DNA-binding superfamily protein |
|  | 1607122_at | 2.19 | Homeobox-leucine zipper protein family |
|  | 1618700_at | 2.14 | Ribonuclease II/R family protein |
|  | 1617037_at | 2.11 | Arginine/serine-rich 6 (Pre-mRNA splicing factor SRP55 |
|  | 1606975_at | 2.08 | Putative ethylene response factor ERF3a |
|  | 1619305_at | 2.05 | Tubby like protein 10 |
|  | 1619985_at | 2.02 | GRAS family transcription factor |
|  | 1618229_at | 0.48 | DNA-directed RNA polymerase |
|  | 1616899_at | 0.48 | Alfin-like 5 |
|  | 1619972_at | 0.48 | MYB transcription factor-like |
|  | 1608639_at | 0.47 | AGAMOUS-like 20 |
|  | 1609295_at | 0.46 | Homeobox 7 |
|  | 1619334_at | 0.45 | GRAS family transcription factor |
|  | 1614385_at | 0.44 | Protein SET DOMAIN GROUP 40 |
|  | 1615073_at | 0.44 | GAGA-binding transcriptional activator BBR/BPC6-like |
|  | 1619830_at | 0.44 | Squamosa promoter binding protein-like 4 |
|  | 1609008_at | 0.43 | Transcriptional regulator, putative |
|  | 1615219_at | 0.42 | Transcriptional coactivator p15 (PC4) family protein (KELP) |
|  | 1621662_at | 0.41 | RNAse THREE-like protein 2 |
| Protein synthesis | 1620491_at | 2.05 | Rafflesia pricei 19S ribosomal RNA gene |
|  | 1622817_at | 0.5 | Putative methionyl-tRNA synthetase |
|  | 1607549_at | 0.45 | Similar to hypothetical protein At5g28500 |
| Protein fate | 1607636_at | 3.89 | Polyubiquitin 3 |
|  | 1615587_at | 3.35 | DnaJ-like protein |
|  | 1617823_at | 2.84 | Protein kinase superfamily protein |
|  | 1619043_at | 2.72 | Serine/threonine protein phosphatase |
|  | 1609028_at | 2.35 | Ubiquitin-conjugating enzyme 32 |
|  | 1622827_at | 2.34 | Insulinase (Peptidase family M16) protein |
|  | 1615115_at | 2.33 | Ubiquitin-conjugating enzyme 22 |
|  | 1612226_at | 2.14 | Phytochrome-associated protein phosphatase type 2C |
|  | 1617047_at | 2.13 | Peptidyl-prolyl cis-trans isomerase |
|  | 1607779_at | 2.06 | Nudix hydrolase homolog 14 |
|  | 1613127_at | 2.03 | Granulin repeat cysteine protease family protein |
|  | 1611873_s_at | 2.02 | RING/U-box superfamily protein |
|  | 1621858_at | 0.5 | Protein phosphatase 2A, regulatory subunit PR55 |
|  | 1610684_at | 0.49 | Chloroplast heat shock protein 70-1 |
|  | 1608701_at | 0.49 | GroES-like family protein |
|  | 1609111_at | 0.49 | TCP-1/cpn60 chaperonin family protein |
|  | 1622801_at | 0.49 | RING/U-box superfamily protein |
|  | 1619591_at | 0.48 | Hydroxyproline-rich glycoprotein family protein |
|  | 1608808_at | 0.45 | Receptor protein kinase PERK1-like protein |
|  | 1620508_at | 0.45 | Similar to cysteine protease Cp5 |
|  | 1612574_s_at | 0.45 | RING/U-box superfamily protein |
|  | 1617731_at | 0.44 | RING/U-box superfamily protein |
|  | 1621666_at | 0.44 | Putativepod-specific dehydrogenase SAC25 |
|  | 1616194_at | 0.41 | Gamma-glutamyl hydrolase 2 |
|  | 1612291_at | 0.4 | Calreticulin 1b |
|  | 1616369_at | 0.39 | Chloroplast heat shock protein 70-2 |
|  | 1612247_at | 0.38 | RING/U-box superfamily protein |
|  | 1614346_at | 0.36 | DNAJ heat shock N-terminal domain-containing protein |
| Protein with binding function | 1622092_at | 2.54 | DNA-binding bromodomain-containing protein |
|  | 1611479_at | 2.43 | Zinc finger protein |
|  | 1607923_at | 0.47 | RNA-binding (RRM/RBD/RNP motifs) family protein |
|  | 1618411_at | 0.46 | PLATZ transcription factor family protein |
| Transport regulation | 1616788_at | 6 | Nitrate transporter2.5 |
|  | 1616083_at | 3.64 | Hexose transporter |
|  | 1608136_s_at | 3.18 | Zinc transporter 5 precursor |
|  | 1609973_at | 2.89 | ABC transporter |
|  | 1608751_at | 2.42 | Cationic amino acid transporter 5 |
|  | 1617166_s_at | 2.42 | Anthocyanidin reductase |
|  | 1619979_at | 2.41 | Vacuolar protein sorting 45 |
|  | 1611719_at | 2.36 | MATE efflux family protein |
|  | 1620591_at | 2.32 | Mitochondrial substrate carrier family protein |
|  | 1620486_at | 2.24 | Cation/H+ exchanger 20 |
|  | 1618682_at | 2.23 | Zinc transporter 5 precursor |
|  | 1607669_at | 2.18 | Major facilitator superfamily protein |
|  | 1620088_at | 2.17 | Membrane transport protein-like |
|  | 1615843_at | 2.14 | Uncoupling protein 5 |
|  | 1614489_at | 2.13 | Aquaporin PIP1 |
|  | 1618942_at | 2.11 | Phosphate transporter 1;9 |
|  | 1607339_at | 2.11 | Vacuolar protein sorting 45 |
|  | 1617495_at | 2.1 | Transmembrane amino acid transporter family protein |
|  | 1621529_at | 2.07 | Amino acid permease 7 |
|  | 1606974_at | 2.06 | Rer1 family protein |
|  | 1618772_at | 2.04 | ENTH/VHS/GAT family protein |
|  | 1619961_at | 0.5 | Sec14p-like phosphatidylinositol transfer family protein |
|  | 1620670_at | 0.49 | Sugar transporter protein 7 |
|  | 1612780_at | 0.49 | Inorganic H pyrophosphatase family protein |
|  | 1609615_at | 0.48 | Translocon at the outer envelope membrane of chloroplasts 34 |
|  | 1613358_at | 0.48 | Vacuolar membrane ATPase 10 |
|  | 1613918_at | 0.47 | Sec14p-like phosphatidylinositol transfer family protein |
|  | 1613638_at | 0.47 | Major facilitator superfamily protein |
|  | 1616748_at | 0.47 | Nascent polypeptide-associated complex subunit alpha-like protein 2 |
|  | 1618459_at | 0.47 | 2-oxoglutarate (2OG) and Fe(II)-dependent oxygenase superfamily protein |
|  | 1609219_at | 0.46 | Mitochondrial substrate carrier family protein |
|  | 1618658_at | 0.46 | Fiber annexin |
|  | 1611299_s_at | 0.42 | Nucleotide-sugar transporter family protein |
|  | 1612083_at | 0.42 | Sec14p-like phosphatidylinositol transfer family protein |
|  | 1610562_at | 0.4 | Transmembrane amino acid transporter family protein |
|  | 1606944_at | 0.38 | Syntaxin/t-SNARE family protein |
|  | 1617910_at | 0.35 | Autoinhibited H(+)-ATPase isoform 10 |
|  | 1620743_at | 0.33 | Vamp/synaptobrevin-associated protein 27-2 |
|  | 1614034_at | 0.3 | Major facilitator superfamily protein |
| Signal transduction | 1617036_at | 2.85 | NAD kinase 1 |
|  | 1621642_at | 2.82 | Inositol 1,3,4-trisphosphate 5/6-kinase family protein |
|  | 1613708_at | 2.57 | Ring zinc finger protein |
|  | 1620322_at | 2.56 | Ras-related protein Rab11C |
|  | 1612777_at | 2.42 | Heptahelical transmembrane protein2 |
|  | 1613535_at | 2.4 | 3'-phosphoinositide-dependent protein kinase 1 |
|  | 1615113_at | 2.37 | Calcium-binding EF-hand family protein |
|  | 1620196_at | 2.34 | Similar to hypothetical protein At2g45590 |
|  | 1612958_at | 2.31 | Protein kinase superfamily protein |
|  | 1620855_at | 2.29 | MAP kinase/ ERK kinase 1 |
|  | 1619478_at | 2.28 | Similar to At5g12010 |
|  | 1616234_at | 2.21 | Protein kinase superfamily protein |
|  | 1620375_at | 2.18 | Shaggy-related kinase 11 |
|  | 1612955_at | 2.18 | Hypothetical protein P0018A03.3 |
|  | 1606455_at | 2.13 | PR5-like receptor kinase |
|  | 1606903_s_at | 2.12 | Calcium-binding EF-hand family protein |
|  | 1612098_x_at | 2.09 | Calcium-binding EF-hand family protein |
|  | 1615362_s_at | 2.04 | MAP kinase 4 |
|  | 1617441_at | 2.03 | Calcium-dependent protein kinase 1 |
|  | 1614905_at | 0.5 | Calcineurin B-like 3 |
|  | 1619559_at | 0.45 | CBL-interacting protein kinase 9 |
|  | 1610761_at | 0.43 | G protein alpha subunit 1 |
|  | 1614811_at | 0.41 | D6 protein kinase |
|  | 1614917_at | 0.32 | IQ-domain 29 |
| Cell rescue | 1617100_at | 3.22 | Glutathione S-transferase tau 7 |
|  | 1617423_at | 2.92 | Glutathione S-transferase family protein |
|  | 1617308_at | 2.83 | Disease resistance-responsive (dirigent-like protein) family protein |
|  | 1612108_at | 2.8 | Avr9/Cf-9 rapidly elicited protein 146 |
|  | 1620030_at | 2.32 | Calcium-binding EF hand family protein |
|  | 1622129_s_at | 2.12 | Fibrillin |
|  | 1614543_a_at | 2.03 | Stress enhanced protein 2 |
|  | 1607971_at | 2.01 | Stress enhanced protein 2 |
|  | 1611765_at | 2.01 | Senescence associated gene 20 |
|  | 1616657_at | 0.49 | Superoxide dismutase [Cu-Zn] |
|  | 1606595_at | 0.49 | Glutathione S-transferase tau 7 |
|  | 1622797_at | 0.48 | Aluminium induced protein with YGL and LRDR motifs |
|  | 1622090_at | 0.48 | Glutaredoxin |
|  | 1615377_at | 0.48 | Immunoglobulin E-set superfamily protein |
|  | 1615226_at | 0.46 | CAX-interacting protein 2 |
|  | 1608025_at | 0.46 | Josephin family protein |
|  | 1611740_at | 0.45 | Machado-Joseph disease-like protein |
|  | 1615639_a_at | 0.42 | Late embryogenesis abundant protein, group 2 |
|  | 1614652_at | 0.31 | Protein kinase protein with tetratricopeptide repeat domain |
|  | 1622477_at | 0.24 | Similar to hypothetical protein At3g22550 |
| Plant / fungal specific systemic sensing and response | 1615971_a_at | 3.1 | Heavy metal transporter |
|  | 1620413_at | 2.98 | Chaperone protein dnaJ-related |
|  | 1607601_at | 2.69 | 12-oxophytodienoate reductase 2 |
|  | 1618465_at | 2.45 | Glycogen synthase kinase-3 |
|  | 1615877_at | 0.5 | ETO1-like 1 |
|  | 1609302_at | 0.47 | Similar to somatic embryogenesis receptor kinase |
|  | 1610003_a_at | 0.42 | NAD(P)-linked oxidoreductase superfamily protein |
|  | 1608979_at | 0.37 | FRIGIDA-like protein |
| Cell fate | 1620003_at | 3.09 | Xyloglucan endotransglucosylase/hydrolase family protein |
| Transposable elements  development | 1619006_at | 0.39 | Mutator-like transposase |
|  | 1608186_at | 2.1 | Tetraspanin8 |
|  | 1612840_a_at | 0.37 | LOB domain-containing protein 1 |
| Biogenesis of cellular component | 1614868_at | 7.24 | Arabinogalactan-protein precursor |
|  | 1614803_at | 4.09 | Proline-rich protein 1 |
|  | 1619613_at | 3.76 | Bifunctional inhibitor/lipid-transfer protein/seed storage 2S albumin superfamily protein |
|  | 1616822_at | 3.55 | Proline-rich protein 1 |
|  | 1607449_s_at | 3.29 | Proline-rich protein 1 |
|  | 1615789_at | 2.47 | Hydroxyproline-rich glycoprotein precursor |
|  | 1616121_at | 2.01 | Similar to AT4g34150/F28A23_90 |
|  | 1609398_at | 0.42 | Tubulin beta 8 |
|  | 1619519_at | 0.4 | Fasciclin-like arabinogalactan protein 1 |
|  | 1607762_at | 0.4 | SCAR2-like |
|  | 1607744_at | 0.37 | Acylhydrolase superfamily protein |
