# Supplementary material for: Transcriptomic Analysis of Grape (Vitis vinifera L.) Leaves after Exposure to Ultraviolet C Irradiation
Source: PLoS One. 2014 Dec 2;9(12):e113772. doi: 10.1371/journal.pone.0113772 (PMC4252036; doi:10.1371/journal.pone.0113772)
Supplement: Additional file S6 — Probe sets specifically up- and down-regulated at 12 h after exposure to UV-C treatment. This information is available free of charge via the Internet at http://pubs.acs.org. (DOCX) [file pone.0113772.s006.docx]

**Additional file S6** Unique genes up- or down-regulated at 12 h after UV-C treatment

| Category | Probe set ID | Fold change | Gene name description |
| --- | --- | --- | --- |
| Metabolism | 1611233_at | 18.51 | Beta-xylosidase 2 |
|  | 1608791_at | 10.38 | Flavonol synthase |
|  | 1618517_at | 9.7 | Beta-galactosidase |
|  | 1619223_s_at | 8.46 | Sucrose synthase 4 |
|  | 1607476_at | 4.76 | Alanine--glyoxylate aminotransferase 2 homolog 2, mitochondrial precursor |
|  | 1618262_at | 4.3 | Acyl-CoA oxidase 2 |
|  | 1616400_s_at | 3.75 | Glutamate-cysteine ligase |
|  | 1618215_s_at | 3.75 | 2-oxoglutarate (2OG) and Fe(II)-dependent oxygenase superfamily protein |
|  | 1620767_at | 3.73 | Glutamine synthetase |
|  | 1606532_s_at | 3.62 | Acyl carrier protein |
|  | 1614985_at | 3.6 | Aspartate aminotransferase |
|  | 1618157_at | 3.59 | 2-oxoglutarate (2OG) and Fe(II)-dependent oxygenase superfamily protein |
|  | 1614355_at | 3.49 | Fumarylacetoacetase |
|  | 1614643_at | 3.27 | Caffeoyl-CoA O-methyltransferase |
|  | 1607228_at | 3.25 | Resveratrol/hydroxycinnamic acid O-glucosyltransferase |
|  | 1614428_at | 3.23 | Glutamate-cysteine ligase |
|  | 1619079_s_at | 3.23 | Acetoacetyl-CoA thiolase 2 |
|  | 1617144_s_at | 3.17 | Phosphoglycerate mutase |
|  | 1617349_at | 3.16 | Purple acid phosphatase 29 |
|  | 1621317_at | 3.05 | Semialdehyde dehydrogenase family protein |
|  | 1619104_s_at | 3.03 | Dehydratase family |
|  | 1610222_at | 2.99 | ATP citrate lyase (ACL) family protein |
|  | 1615976_at | 2.95 | Peroxisomal 3-ketoacyl-CoA thiolase 4 |
|  | 1617789_at | 2.95 | NAD(P)-binding Rossmann-fold superfamily protein |
|  | 1608781_at | 2.89 | Pectin lyase-like superfamily protein |
|  | 1610812_at | 2.89 | Acyl carrier protein |
|  | 1618259_at | 2.78 | Branched-chain amino acid aminotransferase 5 |
|  | 1620569_s_at | 2.72 | 2-oxoglutarate (2OG) and Fe(II)-dependent oxygenase superfamily protein |
|  | 1613920_at | 2.67 | Cytochrome P450, family 71, subfamily B, polypeptide 37 |
|  | 1608661_at | 2.67 | Methylenetetrahydrofolate reductase |
|  | 1606753_at | 2.64 | AMP-dependent synthetase and ligase family protein |
|  | 1622766_at | 2.62 | ATP-citrate lyase A-3 |
|  | 1618480_s_at | 2.62 | UDP-glcnac-adolichol phosphate glcnac-1-p-transferase |
|  | 1612217_at | 2.61 | Fumarylacetoacetase |
|  | 1620788_at | 2.59 | Similar to ferrodoxin NADP oxidoreductase |
|  | 1620673_at | 2.59 | Fructose-bisphosphate aldolase |
|  | 1608741_s_at | 2.57 | Deoxyxylulose-5-phosphate synthase |
|  | 1610923_a_at | 2.56 | NmrA-like negative transcriptional regulator family protein |
|  | 1610781_at | 2.53 | Cytidine/deoxycytidylate deaminase family protein |
|  | 1618506_at | 2.51 | 4-hydroxyphenylpyruvate dioxygenase |
|  | 1615102_at | 2.51 | Dihydroxy-acid dehydratase |
|  | 1609258_at | 2.51 | Copper amine oxidase family protein |
|  | 1608603_at | 2.45 | O-methyltransferase 1 |
|  | 1622839_at | 2.45 | Alpha/beta-Hydrolases superfamily protein |
|  | 1607889_a_at | 2.44 | UDP-XYL synthase 6 |
|  | 1618839_s_at | 2.43 | ATP-citrate lyase A-3 |
|  | 1608579_at | 2.43 | Resveratrol/hydroxycinnamic acid O-glucosyltransferase |
|  | 1622521_at | 2.4 | Histidinol phosphate aminotransferase 1 |
|  | 1620930_s_at | 2.4 | UDP-XYL synthase 5 |
|  | 1614574_at | 2.28 | Putative lysine decarboxylase family protein |
|  | 1609118_at | 2.26 | Aldehyde dehydrogenase 3H1 |
|  | 1612285_at | 2.24 | 2-oxoglutarate (2OG) and Fe(II)-dependent oxygenase superfamily protein |
|  | 1614191_s_at | 2.24 | O-methyltransferase family protein |
|  | 1613321_at | 2.22 | 2-oxoglutarate dehydrogenase E2 subunit |
|  | 1609849_at | 2.2 | Similar to methyltransferase WBSCR22 |
|  | 1616466_at | 2.19 | Alpha/beta-Hydrolases superfamily protein |
|  | 1609840_at | 2.19 | Peptidoglycan-binding LysM domain-containing protein |
|  | 1622313_at | 2.14 | Aldehyde dehydrogenase 3I1 |
|  | 1622560_at | 2.13 | Vacuolar sorting receptor homolog 1 |
|  | 1618777_at | 2.12 | Tyrosine transaminase family protein |
|  | 1609980_a_at | 2.12 | Spermidine synthase 3 |
|  | 1615085_at | 2.12 | O-methyltransferase family protein |
|  | 1615114_at | 2.11 | Putative ripening-related protein |
|  | 1618794_at | 2.1 | Asparagine synthetase 2 |
|  | 1617024_at | 2.09 | Deoxyxylulose-5-phosphate synthase |
|  | 1617525_at | 2.08 | Arginosuccinate synthase family |
|  | 1609458_at | 2.04 | GMP synthase (glutamine-hydrolyzing) |
|  | 1610801_at | 2.04 | Similar to Glutamate decarboxylase |
|  | 1606675_at | 2.03 | Isopentenyl diphosphate isomerase 1 |
|  | 1613782_at | 2 | Ferredoxin 3 |
|  | 1616379_s_at | 0.5 | NAD(P)-binding Rossmann-fold superfamily protein |
|  | 1621020_at | 0.49 | Dicarboxylate diiron protein |
|  | 1609325_at | 0.49 | NAD(P)-linked oxidoreductase superfamily protein |
|  | 1621982_s_at | 0.49 | NAD(P)-binding Rossmann-fold superfamily protein |
|  | 1613511_at | 0.49 | D-isomer specific 2-hydroxyacid dehydrogenase family protein |
|  | 1618004_s_at | 0.48 | Alanine-2-oxoglutarate aminotransferase 2 |
|  | 1618563_at | 0.48 | S-adenosyl-L-methionine-dependent methyltransferases superfamily protein |
|  | 1606984_at | 0.48 | Glycosyl hydrolase 9A1 |
|  | 1622041_at | 0.48 | Sedoheptulose-bisphosphatase |
|  | 1613014_at | 0.48 | Galactose mutarotase-like superfamily protein |
|  | 1611815_at | 0.48 | Alpha/beta-Hydrolases superfamily protein |
|  | 1618940_at | 0.47 | 1-deoxy-D-xylulose 5-phosphate reductoisomerase |
|  | 1613282_at | 0.47 | Putative glycosyl hydrolase family 5 protein/cellulase ((1-4)-beta- mannan endohydrolase) |
|  | 1608861_at | 0.47 | Geranylgeranyl pyrophosphate synthase-related protein, chloroplast precursor |
|  | 1620135_at | 0.47 | Similar to putative isopropylmalate synthase |
|  | 1613289_at | 0.46 | Fatty acyl-ACP thioesterases B |
|  | 1619632_at | 0.46 | Similar to isoamylase-type starch-debranching enzyme 3 |
|  | 1615252_at | 0.46 | Alpha/beta-Hydrolases superfamily protein |
|  | 1611885_at | 0.46 | Carotenoid isomerase |
|  | 1610627_at | 0.46 | Acyl-carrier-protein |
|  | 1614923_at | 0.45 | Hydroxy methylglutaryl CoA reductase 1 |
|  | 1622887_at | 0.45 | Ferredoxin 1 |
|  | 1618865_at | 0.45 | Alpha-amylase-like 2 |
|  | 1606935_at | 0.45 | Aldehyde dehydrogenase 5F1 |
|  | 1616136_at | 0.45 | Cobalamin-independent synthase family protein |
|  | 1610694_at | 0.45 | Similar to UDP-glucuronic acid decarboxylase 1 isoform 1 |
|  | 1613167_s_at | 0.44 | Sedoheptulose-bisphosphatase |
|  | 1615622_at | 0.44 | APS reductase 1 |
|  | 1607462_at | 0.44 | Phosphoglucomutase/phosphomannomutase family protein |
|  | 1611274_at | 0.43 | Lactate/malate dehydrogenase family protein |
|  | 1614186_at | 0.42 | Aldose 1-epimerase family protein |
|  | 1621216_at | 0.42 | S-adenosyl-L-methionine-dependent methyltransferases superfamily protein |
|  | 1613102_s_at | 0.42 | Phosphoribulokinase |
|  | 1613340_at | 0.42 | Alpha/beta-Hydrolases superfamily protein |
|  | 1620567_at | 0.42 | Aspartate aminotransferase |
|  | 1611161_at | 0.42 | Hydrolase |
|  | 1608896_at | 0.42 | Secondary cell wall-related glycosyltransferase family 47 |
|  | 1614709_at | 0.41 | Alpha/beta-Hydrolases superfamily protein |
|  | 1609853_at | 0.41 | Pyrophosphorylase 4 |
|  | 1619289_at | 0.41 | 5'-nucleotidases;magnesium ion binding |
|  | 1615223_at | 0.41 | Carbamoyl phosphate synthetase A |
|  | 1607938_at | 0.4 | Haloacid dehalogenase-like hydrolase (HAD) superfamily protein |
|  | 1622277_at | 0.4 | Putative glycosyl hydrolase family 5 protein/cellulase ((1-4)-beta- mannan endohydrolase) |
|  | 1617309_at | 0.4 | Aldehyde dehydrogenase 3H1 |
|  | 1621374_at | 0.4 | UbiA prenyltransferase family protein |
|  | 1619759_at | 0.4 | S-formylglutathione hydrolase |
|  | 1615927_s_at | 0.39 | 2Fe-2S ferredoxin-like superfamily protein |
|  | 1621073_at | 0.39 | GDP-D-mannose 3',5'-epimerase |
|  | 1613585_at | 0.39 | Alpha/beta-Hydrolases superfamily protein |
|  | 1622806_at | 0.39 | pfkB-like carbohydrate kinase family protein |
|  | 1611604_at | 0.39 | Glycosyl hydrolase family 38 protein |
|  | 1619828_at | 0.38 | Beta-1,3-glucanase 2 |
|  | 1622549_at | 0.38 | Zinc-binding dehydrogenase family protein |
|  | 1616276_at | 0.36 | Glucose-1-phosphate adenylyltransferase |
|  | 1613891_at | 0.36 | MBOAT (membrane bound O-acyl transferase) family protein |
|  | 1610549_at | 0.36 | Flavin containing amine oxidoreductase family |
|  | 1621737_s_at | 0.35 | Alpha/beta-Hydrolases superfamily protein |
|  | 1614014_at | 0.34 | Serine acetyltransferase 2;2 |
|  | 1613382_at | 0.34 | NAD(P)-linked oxidoreductase superfamily protein |
|  | 1613697_at | 0.34 | Glutamine synthetase |
|  | 1615916_at | 0.34 | Haloacid dehalogenase-like hydrolase (HAD) superfamily protein |
|  | 1620058_at | 0.34 | Beta glucosidase 42 |
|  | 1611780_at | 0.34 | Similar to granule-bound starch synthase |
|  | 1621899_at | 0.33 | Zeta-carotene desaturase |
|  | 1612196_at | 0.33 | S-adenosylmethionine decarboxylase proenzyme |
|  | 1607939_at | 0.33 | Caffeoyl-CoA O-methyltransferase |
|  | 1614487_at | 0.33 | Hydroxy methylglutaryl CoA reductase 1 |
|  | 1613666_at | 0.31 | Pectinacetylesterase family protein |
|  | 1611061_at | 0.3 | GDSL-like Lipase/Acylhydrolase superfamily protein |
|  | 1622368_at | 0.3 | Catalytic LigB subunit of aromatic ring-opening dioxygenase family |
|  | 1606522_at | 0.3 | Alpha/beta-Hydrolases superfamily protein |
|  | 1607579_at | 0.3 | GDHB glutamate dehydrogenase |
|  | 1621053_at | 0.3 | pfkB-like carbohydrate kinase family protein |
|  | 1612134_at | 0.29 | Anthocyanidin reductase |
|  | 1618616_at | 0.29 | Plastid transcriptionally active 5 |
|  | 1616659_at | 0.29 | Alanine:glyoxylate aminotransferase |
|  | 1607223_at | 0.27 | Plant L-ascorbate oxidase |
|  | 1608562_at | 0.26 | Ferredoxin--NADP reductase |
|  | 1622361_s_at | 0.25 | Cyclase |
|  | 1616851_at | 0.25 | Sterol 1 |
|  | 1613945_at | 0.24 | Beta-xylosidase 3 |
|  | 1621880_s_at | 0.23 | Glycosyl hydrolase family protein |
|  | 1618284_at | 0.22 | Sedoheptulose-bisphosphatase |
|  | 1615046_at | 0.21 | NADPH dehydrogenase/ oxidoreductase/ poly(U) binding |
|  | 1607774_at | 0.21 | FAD/NAD(P)-binding oxidoreductase family protein |
|  | 1614173_at | 0.2 | Cellulose synthase A4 |
|  | 1620201_at | 0.17 | Pectinesterase |
|  | 1607360_at | 0.17 | Solanesyl diphosphate synthase 1 |
|  | 1617875_at | 0.16 | Beta-hexosaminidase 3 |
|  | 1608196_at | 0.03 | Beta-amylase 1 |
| Engery | 1616605_at | 8.23 | Pheophorbide a oxygenase family protein with Rieske [2Fe-2S] domain |
|  | 1612015_at | 3.39 | Succinyl-CoA ligase, alpha subunit |
|  | 1620526_at | 2.71 | Cytosolic NADP+-dependent isocitrate dehydrogenase |
|  | 1621161_at | 2.68 | Aconitase 1 |
|  | 1608673_at | 2.59 | Cytochrome C1 family |
|  | 1608530_at | 2.42 | Phosphoglycerate mutase family protein |
|  | 1612775_at | 2.39 | Similar to Enolase 2 |
|  | 1614989_at | 2.38 | Cytochome C-1 |
|  | 1611435_s_at | 2.32 | Ubiquinol-cytochrome C reductase complex 6.7 kDa protein |
|  | 1608235_at | 2.3 | Glycine decarboxylase P-protein 2 |
|  | 1608614_at | 2.26 | Fructose-bisphosphate aldolase |
|  | 1609004_s_at | 2.25 | Similar to Enolase 2 |
|  | 1616630_at | 2.12 | Phosphoenolpyruvate carboxykinase 1 |
|  | 1615254_at | 2.07 | Cytochrome bd ubiquinol oxidase, 14kDa subunit |
|  | 1621088_at | 0.5 | NADH-ubiquinone oxidoreductase 24 kDa subunit |
|  | 1609380_x_at | 0.5 | Ribulose-1,5-bisphophate carboxylase/oxygenase small subunit |
|  | 1617670_at | 0.49 | Triosephosphate isomerase |
|  | 1617751_s_at | 0.49 | Lipoxygenase |
|  | 1608207_at | 0.48 | Aldehyde dehydrogenase |
|  | 1609421_at | 0.48 | NAD(P)H dehydrogenase B2 |
|  | 1620919_at | 0.46 | Cytochrome b6-f complex iron-sulfur subunit |
|  | 1618636_at | 0.46 | Serine hydroxymethyltransferase |
|  | 1611515_s_at | 0.45 | Photosystem I subunit H2 |
|  | 1613691_s_at | 0.45 | Photosystem II light harvesting complex gene 2.1 |
|  | 1614270_at | 0.44 | Rubisco methyltransferase family protein |
|  | 1611154_at | 0.43 | Pyruvate kinase |
|  | 1622249_at | 0.43 | Aldolase-type TIM barrel family protein |
|  | 1612272_at | 0.41 | Fructose-1,6-bisphosphatase, cytosolic |
|  | 1611733_s_at | 0.41 | Photosystem I subunit D-2 |
|  | 1616091_at | 0.4 | Fructose-1,6-bisphosphatase, cytosolic |
|  | 1610124_at | 0.4 | Ribulose bisphosphate carboxylase small chain |
|  | 1614016_at | 0.38 | Pyruvate kinase |
|  | 1607655_at | 0.38 | Photosystem I subunit F |
|  | 1617771_at | 0.37 | Photosystem I reaction centre subunit IV / PsaE protein |
|  | 1622302_s_at | 0.36 | PS II oxygen-evolving complex 1 |
|  | 1608859_at | 0.34 | Phosphoglycerate mutase family protein |
|  | 1610656_s_at | 0.32 | Phosphoglycerate mutase family protein |
|  | 1609310_at | 0.31 | Phosphoglycerate kinase |
|  | 1618370_at | 0.3 | Photosystem I subunit l |
|  | 1612273_at | 0.3 | Photosystem II light harvesting complex gene B1B2 |
|  | 1622715_s_at | 0.29 | Glyceraldehyde 3-phosphate dehydrogenase A subunit 2 |
|  | 1614593_at | 0.29 | Light harvesting complex of photosystem II 5 |
|  | 1613494_s_at | 0.28 | Photosystem II subunit P-1 |
|  | 1617066_at | 0.27 | Glycine decarboxylase P-protein 1 |
|  | 1613941_at | 0.27 | Lipoxygenase |
|  | 1614317_at | 0.27 | PS II oxygen-evolving complex 1 |
|  | 1617428_at | 0.26 | Glyceraldehyde-3-phosphate dehydrogenase B subunit |
|  | 1614409_at | 0.26 | Photosystem I light harvesting complex gene 2 |
|  | 1622514_at | 0.25 | Phosphoglycerate/bisphosphoglycerate mutase family protein |
|  | 1614178_at | 0.25 | Rubisco activase |
|  | 1611476_at | 0.25 | Photosystem I P subunit |
|  | 1613332_at | 0.23 | Malate dehydrogenase |
|  | 1611364_at | 0.23 | Photosystem I reaction center subunit III |
|  | 1615822_at | 0.23 | Light-harvesting chlorophyll B-binding protein 3 |
|  | 1613447_s_at | 0.22 | Photosystem I light harvesting complex gene 3 |
|  | 1614784_s_at | 0.21 | Photosystem I light harvesting complex gene 3 |
|  | 1622534_at | 0.21 | Photosystem I reaction center subunit PSI-N, chloroplast, putative / PSI-N, putative (PSAN) |
|  | 1616533_at | 0.19 | ATPase, F0 complex, subunit B/B', bacterial/chloroplast |
|  | 1613773_s_at | 0.18 | Photosystem II reaction center W |
|  | 1618127_at | 0.15 | Photosystem I light harvesting complex gene 3 |
|  | 1614023_at | 0.07 | High cyclic electron flow 1)] |
| Cell cyecle and DNA processing | 1611802_at | 3 | Winged-helix DNA-binding transcription factor family protein |
|  | 1616293_at | 2.77 | Histone deacetylase 3 |
|  | 1606766_a_at | 2.45 | Winged-helix DNA-binding transcription factor family protein |
|  | 1621875_s_at | 2.36 | P-loop containing nucleoside triphosphate hydrolases superfamily protein |
|  | 1612053_at | 2.34 | Histone deacetylase 2a |
|  | 1615409_at | 2.32 | Replication factor A-like protein |
|  | 1615528_a_at | 2.29 | Histone H1 |
|  | 1615214_at | 2.2 | Transducin family protein / WD-40 repeat family protein |
|  | 1617583_at | 2.1 | Ribosome-sedimenting protein |
|  | 1621090_at | 2.09 | P-loop containing nucleoside triphosphate hydrolases superfamily protein |
|  | 1607895_at | 2.09 | Cyclin family protein |
|  | 1618728_at | 2.06 | SET-domain-containing protein |
|  | 1616537_at | 0.43 | DNA topoisomerase I alpha |
|  | 1617718_at | 0.39 | DNA topoisomerase 1 beta |
|  | 1607332_at | 0.39 | FTSH protease 7 |
|  | 1608820_at | 0.25 | P-loop containing nucleoside triphosphate hydrolases superfamily protein |
| Transcription | 1610480_at | 13.97 | NAC domain containing protein 1 |
|  | 1609629_at | 7.49 | AP2 domain transcription factor-like |
|  | 1618514_at | 3.12 | MYB transcription factor |
|  | 1617104_at | 3.12 | Fibrillarin 2 |
|  | 1609021_at | 2.78 | MYB transcription factor |
|  | 1621264_at | 2.74 | Transducin/WD40 repeat-like superfamily protein |
|  | 1613133_at | 2.37 | Similar to putative C-type U1 snRNP |
|  | 1617351_at | 2.33 | TATA binding protein associated factor 21kDa subunit |
|  | 1621462_at | 2.32 | rpb5 RNA polymerase subunit family protein |
|  | 1611826_at | 2.28 | Splicing factor-like protein |
|  | 1611183_at | 2.19 | GATA type zinc finger transcription factor family protein |
|  | 1614801_at | 2.18 | P-loop containing nucleoside triphosphate hydrolases superfamily protein |
|  | 1620219_at | 2.18 | P-loop containing nucleoside triphosphate hydrolases superfamily protein |
|  | 1613873_at | 2.16 | Putative bZIP protein |
|  | 1617969_at | 2.15 | Nucleotide binding |
|  | 1606525_at | 2.14 | Small nuclear ribonucleoprotein family protein |
|  | 1609957_at | 2.11 | Prohibitin 3 |
|  | 1622296_at | 2.09 | G-box binding factor 6 |
|  | 1617678_at | 2.09 | U3 ribonucleoprotein (Utp) family protein |
|  | 1621074_s_at | 2.04 | Transcription elongation factor 1 homolog |
|  | 1612549_at | 2 | Putative RSZp22 splicing factor |
|  | 1617809_at | 0.5 | GRAS family transcription factor |
|  | 1612834_at | 0.5 | Homeodomain-like superfamily protein |
|  | 1607451_at | 0.49 | Sec14p-like phosphatidylinositol transfer family protein |
|  | 1609540_at | 0.49 | FtsH extracellular protease family |
|  | 1614932_at | 0.49 | Duplicated homeodomain-like superfamily protein |
|  | 1618968_at | 0.48 | Homeodomain-like superfamily protein |
|  | 1620757_at | 0.48 | GATA transcription factor 27 |
|  | 1621556_s_at | 0.46 | Homeobox protein 6 |
|  | 1615970_at | 0.45 | Drought responsive element binding protein |
|  | 1610416_at | 0.45 | Glucose-1-phosphate adenylyltransferase |
|  | 1610258_at | 0.44 | T1K7.16 protein |
|  | 1621827_at | 0.44 | Similar to flowering locus C-like MADS-box protein |
|  | 1621626_at | 0.43 | CONSTANS-like 4 |
|  | 1616843_at | 0.42 | D-tyrosyl-tRNA(Tyr) deacylase |
|  | 1620661_at | 0.41 | Homeobox protein 23 |
|  | 1612503_at | 0.4 | Basic helix-loop-helix (bHLH) DNA-binding superfamily protein |
|  | 1613430_a_at | 0.4 | BolA-like family protein |
|  | 1615303_at | 0.39 | Calmodulin-binding transcription activator protein with CG-1 and Ankyrin domains |
|  | 1620298_at | 0.39 | Tubby like protein 6 |
|  | 1620170_at | 0.38 | Related to AP2.7 |
|  | 1617480_at | 0.38 | ZIM-like 1 |
|  | 1610238_s_at | 0.36 | FtsH extracellular protease family |
|  | 1609745_at | 0.36 | BZIP transcription factor bZIP78 |
|  | 1613123_at | 0.34 | Basic helix-loop-helix (bHLH) DNA-binding superfamily protein |
|  | 1609074_at | 0.33 | SCARECROW transcriptional regulator-like |
|  | 1610633_at | 0.33 | B-box type zinc finger family protein |
|  | 1622642_at | 0.31 | Transcriptional coactivator/pterin dehydratase |
|  | 1607973_at | 0.3 | AGAMOUS-like MADS-box protein |
|  | 1608511_at | 0.27 | Ethylene responsive element binding factor 5 |
|  | 1610494_at | 0.27 | Homeodomain GLABROUS 2 |
|  | 1615961_at | 0.26 | NAD(P)-binding Rossmann-fold superfamily protein |
|  | 1611910_s_at | 0.25 | Similar to putative ethylene response factor ERF3b |
|  | 1609990_at | 0.2 | Putative ethylene response factor ERF3b |
| Protein synthesis | 1612076_at | 3.94 | R-protein L3 B |
|  | 1607140_at | 3.39 | Similar to 40S ribosomal S4 protein |
|  | 1612726_at | 3.35 | Ribosomal protein S13A |
|  | 1613008_at | 3.25 | Putative acidic ribosomal protein P1a |
|  | 1617246_s_at | 3 | Similar to 40S ribosomal S4 protein |
|  | 1616689_at | 3 | Ribosomal protein S11 family protein |
|  | 1619423_at | 2.98 | Ribosomal protein L4/L1 family |
|  | 1610548_at | 2.93 | Ribosomal protein S11 family protein |
|  | 1608333_at | 2.91 | Ribosomal protein S8 |
|  | 1607828_at | 2.87 | Nucleolar essential protein-related |
|  | 1618190_at | 2.87 | 40S ribosomal protein S18 |
|  | 1617268_at | 2.85 | Eukaryotic initiation factor 4E protein |
|  | 1619926_at | 2.83 | Translation protein SH3-like family protein |
|  | 1612476_at | 2.74 | Ribosomal protein S3 family protein |
|  | 1610393_at | 2.74 | Translation initiation factor 3G1 |
|  | 1615243_at | 2.72 | Ribosomal protein L16p/L10e family protein |
|  | 1617806_at | 2.71 | Translation initiation factor 2 |
|  | 1610517_s_at | 2.69 | Ribosomal protein S4 |
|  | 1618074_s_at | 2.68 | Ribosomal protein L31e family protein |
|  | 1616595_at | 2.66 | Tyrosyl-tRNA synthetase |
|  | 1609125_at | 2.65 | 60S ribosomal protein L27 |
|  | 1620727_at | 2.61 | Similar to RPL18AA (60S RIBOSOMAL PROTEIN L18A-1); structural constituent of ribosome isoform 2 |
|  | 1607384_at | 2.6 | Ribosomal L22e protein family |
|  | 1613543_s_at | 2.59 | Translation protein SH3-like family protein |
|  | 1612743_at | 2.58 | RNA binding Plectin/S10 domain-containing protein |
|  | 1615029_at | 2.56 | Translation elongation factor EF1B, gamma chain |
|  | 1613237_at | 2.55 | Ribosomal protein S5 domain 2-like superfamily protein |
|  | 1611006_s_at | 2.54 | Ribosomal protein S29 |
|  | 1609713_at | 2.53 | Ribosomal protein L1p/L10e family |
|  | 1610139_s_at | 2.52 | Ribosomal protein L6 family |
|  | 1614840_at | 2.52 | Translation initiation factor 3 subunit H1 |
|  | 1614108_at | 2.51 | Ribosomal protein L35Ae family protein |
|  | 1617765_at | 2.51 | Ribosomal protein S4 |
|  | 1610767_at | 2.51 | Ribosomal protein 5B |
|  | 1609318_at | 2.51 | Ribosomal protein L18e/L15 superfamily protein |
|  | 1612602_at | 2.49 | Ribosomal protein 5B |
|  | 1619463_s_at | 2.47 | 60S ribosomal protein L41 |
|  | 1612626_at | 2.47 | Ribosomal L29 family protein |
|  | 1607974_at | 2.46 | Ribosomal protein L7Ae/L30e/S12e/Gadd45 family protein |
|  | 1609098_at | 2.44 | Translation initiation factor 3G1 |
|  | 1622063_s_at | 2.43 | Translation initiation factor 4E |
|  | 1621039_s_at | 2.42 | Ribosomal protein S6 |
|  | 1612656_at | 2.4 | Ribosomal protein L30/L7 family protein |
|  | 1613150_at | 2.39 | Ribosomal protein L23AA |
|  | 1612610_s_at | 2.39 | Ribosomal protein 5B |
|  | 1617136_s_at | 2.36 | Ribosomal protein L15 |
|  | 1610619_at | 2.35 | 60S ribosomal protein L36 |
|  | 1613721_at | 2.34 | Zinc-binding ribosomal protein family protein |
|  | 1610095_s_at | 2.33 | 60S ribosomal protein L13 |
|  | 1608162_at | 2.33 | Ribosomal protein L13 family protein |
|  | 1621330_at | 2.33 | Translation protein SH3-like family protein |
|  | 1612782_at | 2.32 | Ribosomal protein L5 B |
|  | 1611286_s_at | 2.3 | Thaliana 60S ribosomal protein L7 |
|  | 1620178_s_at | 2.29 | Similar to protein synthesis initiation factor-like |
|  | 1609377_at | 2.29 | Similar to RPL16A (ribosomal protein large subunit 16A); structural constituent of ribosome |
|  | 1614171_at | 2.28 | Ribosomal protein L22p/L17e family protein |
|  | 1621433_s_at | 2.26 | Ribosomal protein S12/S23 family protein |
|  | 1619416_at | 2.26 | 40S ribosomal protein S9-2 |
|  | 1619008_x_at | 2.24 | Ribosomal protein S7e family protein |
|  | 1615368_at | 2.23 | Probable ribosome biogenesis protein RLP24 |
|  | 1621771_at | 2.2 | 40S ribosomal protein SA |
|  | 1616502_at | 2.18 | Ribosomal protein S19 family protein |
|  | 1611082_s_at | 2.17 | 60S ribosomal protein L10 |
|  | 1616916_a_at | 2.17 | 60S ribosomal protein L36 |
|  | 1614315_at | 2.16 | Class II aminoacyl-tRNA and biotin synthetases superfamily protein |
|  | 1611315_at | 2.15 | Zinc-binding ribosomal protein family protein |
|  | 1611122_at | 2.13 | Ribosomal protein S4 |
|  | 1608266_at | 2.13 | 60S ribosomal protein L36 |
|  | 1615334_at | 2.11 | Ribosomal L28e protein family |
|  | 1613593_at | 2.11 | Seryl-tRNA synthetase / serine--tRNA ligase |
|  | 1608064_at | 2.1 | Ribosomal protein L18 |
|  | 1620764_a_at | 2.1 | Elongation factor 1-alpha |
|  | 1616266_at | 2.08 | 60S acidic ribosomal protein family |
|  | 1619375_at | 2.08 | Eukaryotic elongation factor 5A-1 |
|  | 1609755_s_at | 2.07 | Ribosomal protein L27a |
|  | 1621763_s_at | 2.07 | 60S ribosomal protein L13 |
|  | 1608129_at | 2.07 | Similar to RPL18AA (60S RIBOSOMAL PROTEIN L18A-1) |
|  | 1608540_at | 2.06 | 60S ribosomal protein L12 |
|  | 1607035_at | 2.06 | MIF4G domain-containing protein / MA3 domain-containing protein |
|  | 1619943_at | 2.05 | Ribosomal protein S6e |
|  | 1609112_s_at | 2.05 | Ribosomal protein S19e family protein |
|  | 1611294_at | 2.05 | Ribosomal protein S3 family protein |
|  | 1619809_at | 2.05 | Eukaryotic translation initiation factor 3G1 |
|  | 1616442_a_at | 2.04 | Ribosomal protein S25 family protein |
|  | 1615465_at | 2.03 | Similar to protein synthesis initiation factor-like |
|  | 1618220_at | 2.03 | Ribosomal protein S8 |
|  | 1620016_at | 2.03 | Translation initiation factor 4E |
|  | 1607085_at | 2.02 | Transcript elongation factor IIS |
|  | 1621535_s_at | 2.01 | Ribosomal protein L2 family |
|  | 1612751_at | 2.01 | Putative translation initiation factor IF-2 |
|  | 1608867_s_at | 2 | Ribosomal protein L31e family protein |
|  | 1607415_at | 2 | Putative translation-initiation factor 3 subunit |
|  | 1622099_at | 0.48 | Guanine nucleotide-binding protein subunit beta-like protein |
|  | 1618474_at | 0.47 | Homologue to emb\|Y18934.1\|CPY18934 Solanum nigrum chloroplast tRNA-Ala, tRNA-Ile, 16S rRNA, tRNA-Val, rps12, rps7, ndhB genes |
|  | 1618547_at | 0.46 | Class II aaRS and biotin synthetases superfamily protein |
|  | 1609656_s_at | 0.45 | Homologue to emb\|Y18934.1\|CPY18934 Solanum nigrum Chloroplast tRNA-Ala, tRNA-Ile, 16S rRNA, tRNA-Val, rps12, rps7, ndhB genes |
|  | 1612453_at | 0.44 | Seryl-tRNA synthetase |
|  | 1608515_at | 0.43 | Methionine--tRNA ligase, putative / methionyl-tRNA synthetase, putative / MetRS, putative |
|  | 1617204_at | 0.37 | Ribosomal protein S5 family protein |
|  | 1616415_at | 0.36 | tRNA synthetase class I (I, L, M and V) family protein |
|  | 1616063_at | 0.35 | Class II aminoacyl-tRNA and biotin synthetases superfamily protein |
|  | 1622193_at | 0.33 | Ribosomal protein S1 |
|  | 1608467_at | 0.33 | Translation initiation factor IF-2 |
|  | 1612544_at | 0.32 | Ribosomal protein large subunit 27 |
|  | 1612258_at | 0.3 | Translation initiation factor IF-2 |
|  | 1619587_at | 0.25 | Ribosomal protein S14p/S29e family protein |
|  | 1613425_at | 0.24 | Alanyl-tRNA synthetase, class Iic |
| Protein fate | 1616145_a_at | 6.35 | Heat shock protein 18.2 |
|  | 1614330_at | 4.27 | 17.6 kDa class II heat shock protein |
|  | 1620960_a_at | 4.11 | Heat shock protein 18.2 |
|  | 1614457_at | 3.47 | Regulatory particle non-ATPase 12A |
|  | 1620260_s_at | 3.39 | AAA-type ATPase family protein |
|  | 1616811_at | 3.11 | DNAJ heat shock family protein |
|  | 1617508_a_at | 2.93 | Alpha/beta-Hydrolases superfamily protein |
|  | 1615660_at | 2.9 | Ubiquitin-protein ligase |
|  | 1621146_s_at | 2.84 | Regulatory particle non-ATPase 12A |
|  | 1607728_at | 2.83 | RING-H2 finger B1A |
|  | 1621091_at | 2.8 | RP non-ATPase subunit 8A |
|  | 1613741_at | 2.73 | Protein kinase |
|  | 1619430_s_at | 2.56 | Protein disulfide-isomerase precursor |
|  | 1607583_at | 2.51 | ClpX, ATPase regulatory subunit |
|  | 1613460_at | 2.5 | Putative heat shock protein |
|  | 1618118_a_at | 2.48 | Heat shock protein 91 |
|  | 1613042_at | 2.46 | HSP20-like chaperones superfamily protein |
|  | 1613063_at | 2.43 | Ubiquitin related modifier 1 |
|  | 1613672_at | 2.42 | WD40 repeat-like superfamily protein |
|  | 1616246_at | 2.39 | Heat shock cognate 70 kDa protein 1 |
|  | 1608103_at | 2.39 | Protein kinase superfamily protein |
|  | 1610668_at | 2.36 | HAL2-like |
|  | 1615137_at | 2.34 | Calreticulin 1b |
|  | 1619616_at | 2.28 | Heat shock protein 17.4 |
|  | 1609293_at | 2.26 | Methionine aminopeptidase |
|  | 1619075_at | 2.23 | Regulatory particle triple-A ATPase 4A |
|  | 1611053_at | 2.21 | Heat shock protein 70 (Hsp 70) family protein |
|  | 1610364_at | 2.16 | Protein kinase superfamily protein |
|  | 1606494_at | 2.16 | Ubiquitin-conjugating enzyme 32 |
|  | 1617898_at | 2.14 | RING/U-box superfamily protein |
|  | 1616733_s_at | 2.13 | Phosphotyrosine protein phosphatases superfamily protein |
|  | 1617494_at | 2.13 | Transducin/WD40 repeat-like superfamily protein |
|  | 1616170_at | 2.13 | Ubiquitin carrier protein |
|  | 1614224_at | 2.12 | Casein kinase II, alpha chain 2 |
|  | 1609502_at | 2.1 | Heat shock protein 60 |
|  | 1617932_at | 2.1 | SPFH/Band 7/PHB domain-containing membrane-associated protein family |
|  | 1612977_at | 2.09 | Casein kinase II beta chain 2 |
|  | 1609974_at | 2.09 | Serine/threonine protein kinase |
|  | 1615468_s_at | 2.08 | Ubiquitin carrier protein |
|  | 1615059_at | 2.06 | Ubiquitin carboxyl-terminal hydrolase |
|  | 1609978_at | 2.05 | 26S proteasome non-ATPase regulatory subunit 14 |
|  | 1617914_at | 2.04 | DNAJ heat shock N-terminal domain-containing protein |
|  | 1621445_at | 2.04 | Putative FtsH protease |
|  | 1617603_at | 2.02 | Signal recognition particle-related / SRP-related |
|  | 1619577_at | 2.02 | F-box protein 2 |
|  | 1614810_s_at | 2.02 | Ubiquitin-like protein 5 |
|  | 1613724_at | 0.5 | SOS3-interacting protein 1 |
|  | 1609958_at | 0.49 | Similar to protein phosphatase 2a, catalytic subunit, alpha isoform |
|  | 1614135_at | 0.49 | Protein serine/threonine phosphatase |
|  | 1611639_at | 0.49 | Alpha/beta-Hydrolases superfamily protein |
|  | 1622146_at | 0.49 | ATP-dependent Clp protease proteolytic subunit |
|  | 1619172_at | 0.49 | Prolyl oligopeptidase family protein |
|  | 1610958_at | 0.48 | Chloroplast heat shock protein 70-1 |
|  | 1609735_at | 0.48 | Target of rapamycin |
|  | 1618161_at | 0.48 | Alpha/beta-Hydrolases superfamily protein |
|  | 1615924_at | 0.47 | Calcineurin-like metallo-phosphoesterase superfamily protein |
|  | 1612240_at | 0.47 | RING/U-box superfamily protein |
|  | 1622071_a_at | 0.47 | Cytosol aminopeptidase family protein |
|  | 1610574_at | 0.47 | Aminopeptidase M1 |
|  | 1612501_at | 0.46 | ZIK1 protein |
|  | 1608628_at | 0.45 | Cyclophilin 38 |
|  | 1622857_at | 0.45 | ATP-dependent Clp protease proteolytic subunit |
|  | 1609670_at | 0.44 | T-complex protein 11 |
|  | 1608909_at | 0.44 | NEDD8-activating enzyme E1 catalytic subunit |
|  | 1616418_at | 0.44 | Serine carboxypeptidase-like 20 |
|  | 1614058_at | 0.43 | Protein kinase family protein |
|  | 1612759_at | 0.43 | ATP-dependent Clp protease proteolytic subunit |
|  | 1614152_at | 0.42 | Pyrophosphorylase 6 |
|  | 1611714_at | 0.42 | Serine/threonine protein phosphatase |
|  | 1616447_at | 0.42 | RING-H2 group F2A |
|  | 1609580_at | 0.41 | DNAJ heat shock N-terminal domain-containing protein] |
|  | 1611269_s_at | 0.41 | Putative peroxisomal membrane carrier protein |
|  | 1622470_at | 0.41 | Insulinase (Peptidase family M16) family protein |
|  | 1611342_at | 0.4 | Protein kinase family protein |
|  | 1613427_at | 0.4 | Putative phosphoinositide phosphatase |
|  | 1613390_at | 0.4 | Presequence protease 1 |
|  | 1609611_at | 0.4 | ATP-dependent Clp protease proteolytic subunit |
|  | 1620056_s_at | 0.38 | Alpha/beta-Hydrolases superfamily protein |
|  | 1613252_at | 0.37 | ACT-like protein tyrosine kinase family protein |
|  | 1622622_a_at | 0.37 | Similar to peptidase S41 family protein |
|  | 1613539_at | 0.35 | Chloroplast signal recognition particle 54 kDa subunit |
|  | 1617030_at | 0.35 | Putative serine/threonine protein kinase |
|  | 1614151_a_at | 0.35 | ATP-dependent Clp protease proteolytic subunit ClpR4 |
|  | 1609491_at | 0.35 | Similar to peptidase S41 family protein |
|  | 1612727_at | 0.35 | Similar to MAP1D (METHIONINE AMINOPEPTIDASE 1D); metalloexopeptidase |
|  | 1621672_at | 0.33 | Ethylene-dependent gravitropism-deficient and yellow-green-like 2 |
|  | 1612366_at | 0.33 | DegP protease 1 |
|  | 1616214_at | 0.33 | CAAX amino terminal protease family protein |
|  | 1614320_s_at | 0.33 | Similar to MAP1D (METHIONINE AMINOPEPTIDASE 1D); metalloexopeptidase |
|  | 1618172_at | 0.28 | RING/FYVE/PHD zinc finger superfamily protein |
|  | 1616653_at | 0.28 | Ubiquitin carrier protein |
|  | 1621271_at | 0.28 | ATP-dependent protease La (LON) domain protein |
|  | 1612218_at | 0.27 | Ser/Thr protein kinase |
|  | 1619614_at | 0.26 | STRUBBELIG-receptor family 7 |
|  | 1620371_at | 0.12 | Eukaryotic aspartyl protease family protein |
|  | 1611841_at | 0.09 | Subtilase family protein |
| Protein with binding function | 1621637_at | 2.71 | Metallopeptidase M24 family protein |
|  | 1621900_s_at | 2.69 | RNA-binding (RRM/RBD/RNP motifs) family protein |
|  | 1614858_at | 2.59 | Transcribed sequence 1087 protein |
|  | 1622528_at | 2.45 | Copper amine oxidase |
|  | 1618475_at | 2.43 | Transducin/WD40 repeat-like superfamily protein |
|  | 1608844_at | 2.37 | RNA-binding (RRM/RBD/RNP motifs) family protein |
|  | 1607611_at | 2.37 | Similarity to RNA binding protein |
|  | 1618371_at | 2.28 | DNA-binding protein S1FA2 |
|  | 1616265_s_at | 2.27 | NOP56-like pre RNA processing ribonucleoprotein |
|  | 1614879_at | 2.13 | RING/U-box superfamily protein |
|  | 1620697_at | 2.13 | Matrix attachment region binding protein |
|  | 1613596_at | 2.09 | CTC-interacting domain 11 |
|  | 1621606_at | 2.09 | Transducin/WD40 repeat-like superfamily protein |
|  | 1620588_at | 0.49 | Cupredoxin superfamily protein |
|  | 1612409_at | 0.47 | Polcalcin Bet v 4 |
|  | 1611392_at | 0.46 | Mannose-binding lectin superfamily protein |
|  | 1615048_at | 0.45 | Membrane protein |
|  | 1613451_at | 0.44 | ATP binding |
|  | 1619510_s_at | 0.43 | Nitrogen regulatory PII-like, alpha/beta |
|  | 1615761_at | 0.41 | RNA-binding (RRM/RBD/RNP motifs) family protein |
|  | 1611867_at | 0.39 | Calcium-binding EF-hand family protein |
|  | 1613097_at | 0.34 | Remorin family protein |
|  | 1614814_s_at | 0.32 | CP12 domain-containing protein 1 |
|  | 1618718_at | 0.32 | Plastid-lipid associated protein PAP / fibrillin family protein |
|  | 1609667_at | 0.32 | Similar to HCF101; ATP binding |
|  | 1606701_s_at | 0.31 | Remorin family protein |
|  | 1613826_at | 0.3 | Protein containing PDZ domain, a K-box domain, and a TPR region |
|  | 1607311_at | 0.28 | 3'-5' exonuclease domain-containing protein / K homology domain-containing protein / KH domain-containing protein |
|  | 1614022_at | 0.28 | Remorin family protein |
|  | 1615959_at | 0.24 | EF hand calcium-binding protein family |
|  | 1610210_at | 0.23 | Rubredoxin family protein |
| Protein activity regulation  transport regulation | 1615850_s_at | 1.91 | Cysteine proteinase inhibitor |
|  | 1607560_at | 4.63 | MATE efflux family protein |
|  | 1610914_at | 3.98 | Copper transporter 1 |
|  | 1619882_at | 3.41 | Syntaxin of plants 132 |
|  | 1612793_at | 3.24 | Zim17-type zinc finger protein |
|  | 1607599_at | 3.19 | Putative histidine amino acid transporter |
|  | 1616720_at | 3.11 | Acyl-CoA oxidase 4 |
|  | 1622472_at | 3.11 | Translocase of outer membrane 20 kDa subunit 3 |
|  | 1616842_s_at | 3.03 | Translocase of outer membrane 20 kDa subunit 3 |
|  | 1608838_s_at | 2.85 | Thioredoxin reductase |
|  | 1614987_at | 2.8 | MATE efflux family protein |
|  | 1608368_at | 2.73 | Translocase of outer membrane 20 kDa subunit 3 |
|  | 1619594_at | 2.62 | NOD26-like intrinsic protein 5;1 |
|  | 1621782_at | 2.58 | Syntaxin of plants 132 |
|  | 1617063_at | 2.56 | Sulfite reductase |
|  | 1606902_a_at | 2.46 | Voltage dependent anion channel 1 |
|  | 1617115_at | 2.39 | Staurosporin and temperature sensitive 3-like b |
|  | 1614976_at | 2.35 | Delta tonoplast integral protein |
|  | 1614599_at | 2.34 | Sec23/Sec24 protein transport family protein |
|  | 1613145_at | 2.33 | Plant VAMP (vesicle-associated membrane protein) family protein |
|  | 1608420_at | 2.32 | Coated vesicle membrane protein-like |
|  | 1619551_at | 2.29 | SNARE-like superfamily protein |
|  | 1608830_at | 2.28 | Putative syntaxin of plants 52 |
|  | 1611287_at | 2.26 | Thioredoxin family Trp26-like protein |
|  | 1618695_at | 2.23 | H(+)-ATPase 8 |
|  | 1610844_at | 2.21 | Syntaxin of plants 61 |
|  | 1615749_at | 2.18 | Electron transfer flavoprotein beta |
|  | 1607237_at | 2.18 | Tim10/DDP family zinc finger protein |
|  | 1607699_at | 2.17 | Inositol transporter 1 |
|  | 1614117_at | 2.14 | MATE efflux family protein |
|  | 1612169_at | 2.13 | Nascent polypeptide-associated complex (NAC), alpha subunit family protein |
|  | 1621736_at | 2.13 | Electron transport SCO1/SenC family protein |
|  | 1609012_at | 2.1 | ENTH/ANTH/VHS superfamily protein |
|  | 1609729_at | 2.1 | Syntaxin of plants 31 |
|  | 1620355_s_at | 2.08 | Adenine nucleotide translocator |
|  | 1622783_s_at | 2.06 | RAN binding protein 1 |
|  | 1613829_at | 2.04 | Emp24/gp25L/p24 family/GOLD family protein |
|  | 1608531_at | 2.03 | ER lumen protein retaining receptor |
|  | 1622840_at | 2.03 | Clathrin adaptor complexes medium subunit family protein |
|  | 1611896_at | 2.02 | Peptide transporter 2 |
|  | 1616196_at | 2.01 | Vesicle-associated membrane protein 711 |
|  | 1612407_at | 2 | Vesicle transport v-SNARE family protein |
|  | 1606872_at | 0.49 | VPS13-like protein |
|  | 1618605_at | 0.48 | Mitochondrial substrate carrier family protein |
|  | 1613421_at | 0.47 | Mitochondrial substrate carrier family protein |
|  | 1619309_at | 0.47 | P-loop containing nucleoside triphosphate hydrolases superfamily protein |
|  | 1622128_s_at | 0.46 | Putative aquaporin TIP3 |
|  | 1610128_at | 0.46 | Adenosine monophosphate kinase |
|  | 1622397_at | 0.45 | Mitochondrial substrate carrier family protein |
|  | 1615751_at | 0.45 | Non-intrinsic ABC protein 7 |
|  | 1622660_at | 0.44 | ATP binding cassette protein 1 |
|  | 1610264_at | 0.43 | Similar to putative multidrug resistance-associated protein |
|  | 1612725_at | 0.42 | Cation exchanger 3 |
|  | 1617326_at | 0.41 | Integral membrane TerC family protein |
|  | 1609005_at | 0.41 | Major facilitator superfamily protein |
|  | 1606983_at | 0.41 | YELLOW STRIPE like 8 |
|  | 1610603_at | 0.41 | Gamma tonoplast intrinsic protein |
|  | 1611312_s_at | 0.4 | Gamma tonoplast intrinsic protein |
|  | 1617237_s_at | 0.4 | Cation exchanger 3 |
|  | 1620046_at | 0.39 | Maltose excess protein 1, chloroplast precursor |
|  | 1607497_at | 0.39 | 2-cysteine peroxiredoxin B |
|  | 1614231_s_at | 0.39 | Thioredoxin M-type 4 |
|  | 1620288_s_at | 0.39 | Thioredoxin superfamily protein |
|  | 1616229_at | 0.38 | Dicarboxylate transporter 1 |
|  | 1611591_at | 0.37 | Sulfate transmembrane transporters |
|  | 1617645_s_at | 0.37 | Glucose-6-phosphate/phosphate translocator-related |
|  | 1609921_at | 0.37 | Glucose-6-phosphate/phosphate translocator-related |
|  | 1617646_at | 0.37 | Thioredoxin F2 |
|  | 1613473_at | 0.37 | ABC2 homolog 13 |
|  | 1621921_at | 0.37 | P-loop containing nucleoside triphosphate hydrolases superfamily protein |
|  | 1616662_at | 0.35 | ATPase E1-E2 type family protein / haloacid dehalogenase-like hydrolase family protein |
|  | 1621150_at | 0.34 | Transporter associated with antigen processing protein 2 |
|  | 1618001_at | 0.32 | ABC2 homolog 13 |
|  | 1611751_at | 0.32 | Nuclear transport factor 2 (NTF2) family protein |
|  | 1620461_at | 0.31 | Glucose-6-phosphate/phosphate translocator-related |
|  | 1618911_at | 0.3 | Thioredoxin H-type 1 |
|  | 1612701_at | 0.3 | ABC2 homolog 13 |
|  | 1611577_at | 0.28 | Aquaporin PIP1;4 |
|  | 1618622_at | 0.26 | Similar to MATE efflux family protein |
|  | 1614477_s_at | 0.18 | Glutaredoxin family protein |
| Signal transduction | 1609226_at | 12 | Calnexin 1 |
|  | 1619975_at | 4.31 | Leucine-rich receptor-like protein kinase family protein |
|  | 1612249_at | 3.33 | GTP-binding family protein |
|  | 1616324_s_at | 3.08 | Receptor for activated C kinase 1B |
|  | 1610660_at | 3.07 | Malectin/receptor-like protein kinase family protein |
|  | 1613881_at | 2.97 | Membrane-associated progesterone binding protein 3 |
|  | 1606958_s_at | 2.96 | Membrane-associated progesterone binding protein 3 |
|  | 1614995_at | 2.95 | Calcium-dependent protein kinase 21 |
|  | 1615332_at | 2.88 | ENTH/VHS/GAT family protein |
|  | 1608577_s_at | 2.86 | Transducin/WD40 repeat-like superfamily protein |
|  | 1609309_at | 2.73 | Membrane-associated progesterone binding protein 3 |
|  | 1608953_at | 2.6 | Receptor for activated C kinase 1C |
|  | 1618825_at | 2.55 | IQ-domain 13 |
|  | 1618341_s_at | 2.48 | Plant calmodulin-binding protein-related |
|  | 1608684_at | 2.33 | General regulatory factor 9 |
|  | 1613942_at | 2.28 | Protein kinase superfamily protein |
|  | 1622681_at | 2.18 | Protein phosphatase 2C family protein |
|  | 1612612_at | 2.18 | General regulatory factor 2 |
|  | 1616355_at | 2.13 | CBL-interacting protein kinase 23 |
|  | 1616535_at | 2.13 | Calcium-binding EF-hand family protein |
|  | 1609543_at | 2.11 | RAB GTPase homolog B1C |
|  | 1606614_at | 2.1 | Ras-related small GTP-binding family protein |
|  | 1619292_at | 2.08 | Similar to ribokinase |
|  | 1616740_s_at | 2.08 | Similar to ADP-ribosylation factor isoform 1 |
|  | 1608865_at | 2.06 | General regulatory factor 7 |
|  | 1609412_at | 2.05 | Protein phosphatase 2C |
|  | 1613908_at | 2.05 | S-methyl-5-thioribose kinase |
|  | 1607396_s_at | 2.03 | RAB GTPase homolog B18 |
|  | 1615820_at | 0.5 | GTP cyclohydrolase I |
|  | 1607281_at | 0.49 | RAS-related GTP-binding nuclear protein 2 |
|  | 1607338_at | 0.49 | Myo-inositol-1-phosphate synthase 2 |
|  | 1611669_at | 0.48 | RHO protein GDP dissociation inhibitor |
|  | 1612769_at | 0.48 | HPT phosphotransmitter 4 |
|  | 1622867_s_at | 0.47 | Elongation factor family protein |
|  | 1615651_at | 0.47 | Calcium-binding EF-hand family protein |
|  | 1620502_at | 0.44 | Leucine-rich repeat transmembrane protein kinase |
|  | 1616252_at | 0.39 | Calcium-dependent lipid-binding (CaLB domain) family protein |
|  | 1617552_at | 0.39 | RAC-like 1 |
|  | 1617796_at | 0.38 | Cornichon family protein |
|  | 1621638_at | 0.38 | Amidohydrolase family |
|  | 1614852_at | 0.35 | Leucine-rich repeat protein kinase family protein |
|  | 1617073_at | 0.34 | B-box type zinc finger family protein |
|  | 1621415_at | 0.29 | Calcium-binding EF-hand family protein |
|  | 1620671_at | 0.28 | S-locus lectin protein kinase family protein |
|  | 1622772_at | 0.26 | Homology to ABI1 |
|  | 1617247_at | 0.22 | RALF-like 34 |
|  | 1612275_at | 0.21 | Protein kinase superfamily protein |
|  | 1608189_s_at | 0.2 | Protein phosphatase 2CA |
|  | 1611127_at | 0.2 | Calmodulin-like 41 |
|  | 1617697_at | 0.19 | Thylakoid-associated phosphatase 38 |
|  | 1619894_at | 0.09 | General regulatory factor 9 |
| Cell rescue | 1617839_at | 3.17 | Glutathione-disulfide reductase |
|  | 1606514_at | 2.79 | Wound-responsive family protein |
|  | 1621336_at | 2.7 | Stromal ascorbate peroxidase |
|  | 1609231_at | 2.48 | Thylakoidal ascorbate peroxidase |
|  | 1620826_s_at | 2.3 | Thylakoidal ascorbate peroxidase |
|  | 1614035_at | 2.27 | Glutathione S-transferase tau 7 |
|  | 1611384_at | 0.5 | Late embryogenesis abundant protein, group 6 |
|  | 1615403_at | 0.49 | P-loop containing nucleoside triphosphate hydrolases superfamily protein |
|  | 1615139_at | 0.46 | NAD(P)-binding Rossmann-fold superfamily protein |
|  | 1620598_at | 0.46 | Disease resistance gene |
|  | 1609478_s_at | 0.46 | Class III peroxidase GvPx2b |
|  | 1617579_at | 0.45 | Embryonic protein DC-8 |
|  | 1611203_at | 0.45 | Dehydroascorbate reductase 1 |
|  | 1615206_s_at | 0.45 | Glutathione S-transferase |
|  | 1611993_at | 0.43 | Ascorbate peroxidase 3 |
|  | 1616882_at | 0.42 | EXORDIUM like 2 |
|  | 1611871_at | 0.41 | Dehydroascorbate reductase |
|  | 1619199_at | 0.4 | Resistance protein RGC2 |
|  | 1609167_at | 0.4 | Glutathione S-transferase zeta 1 |
|  | 1607482_at | 0.4 | Glutathione S-transferase family protein |
|  | 1611744_s_at | 0.37 | Rubber elongation factor protein (REF) |
|  | 1620356_x_at | 0.35 | Glutathione S-transferase |
|  | 1609024_s_at | 0.32 | Lactoylglutathione lyase / glyoxalase I family protein |
|  | 1609324_at | 0.32 | Glutathione S-transferase |
|  | 1610871_s_at | 0.32 | Catalase |
|  | 1617574_at | 0.29 | Similar to At3g03280 |
|  | 1614776_a_at | 0.28 | Superoxide dismutase [Cu-Zn] |
|  | 1619377_at | 0.25 | AIG2-like (avirulence induced gene) family protein |
|  | 1622115_at | 0.24 | Beta glucosidase 11 |
|  | 1614361_at | 0.24 | Peroxidase superfamily protein |
|  | 1620656_at | 0.23 | Blue-copper-binding protein |
|  | 1610192_at | 0.23 | Early-responsive to dehydration stress protein (ERD4) |
|  | 1610547_at | 0.19 | RGC2-like protein |
|  | 1607880_at | 0.19 | PR5-like receptor kinase |
|  | 1616133_at | 0.16 | Major latex protein |
|  | 1614066_at | 0.16 | Lipase/lipooxygenase |
| Plant / fungal specific systemic sensing and response | 1620574_s_at | 5.56 | Auxin efflux carrier protein |
|  | 1621521_at | 2.89 | Methyl esterase 10 |
|  | 1618880_at | 2.52 | Impaired sucrose induction 1-like protein |
|  | 1619462_at | 2.45 | Nitrilase/cyanide hydratase and apolipoprotein N-acyltransferase family protein |
|  | 1612376_a_at | 2.26 | Impaired sucrose induction 1-like protein |
|  | 1622212_at | 2.1 | TGF-beta receptor-interacting protein 1 |
|  | 1608022_at | 0.48 | 9-cis-epoxycarotenoid dioxygenase 1 |
|  | 1620476_at | 0.48 | Phototropic-responsive NPH3 family protein |
|  | 1618394_at | 0.47 | O-fucosyltransferase family protein |
|  | 1618518_at | 0.46 | Signal transduction histidine kinase, hybrid-type, ethylene sensor |
|  | 1613813_a_at | 0.45 | Auxin response factor 2 |
|  | 1618181_at | 0.44 | Alpha/beta-Hydrolases superfamily protein |
|  | 1620714_at | 0.43 | Phosphate-responsive 1 family protein |
|  | 1606788_s_at | 0.4 | 9-cis-epoxycarotenoid dioxygenase 1 |
|  | 1606913_at | 0.39 | Zeaxanthin epoxidase (ZEP) (ABA1) |
|  | 1616785_at | 0.33 | AUX/IAA transcriptional regulator family protein |
|  | 1613098_at | 0.33 | Phosphate-responsive 1 family protein |
|  | 1618171_s_at | 0.31 | Zeaxanthin epoxidase (ZEP) (ABA1) |
|  | 1615728_at | 0.29 | Indole-3-acetic acid inducible 9 |
|  | 1620309_at | 0.28 | S-adenosyl-L-methionine-dependent methyltransferases superfamily protein |
|  | 1607138_at | 0.25 | NAD(P)-binding Rossmann-fold superfamily protein |
|  | 1612090_s_at | 0.18 | Germin-like protein 6 |
|  | 1621326_at | 0.13 | Oxidoreductase 2OG-Fe(II) oxygenase family protein |
|  | 1620306_at | 0.12 | Cytokinin dehydrogenase 5 precursor |
| Cell fate | 1613952_at | 0.48 | Putative phytosulfokine peptide precursor |
|  | 1609987_at | 0.24 | Rapid alkalinization factor preproprotein precursor |
| Development | 1611296_at | 7.97 | Non-yellowing 1 |
|  | 1616413_at | 2.83 | Osmotin 34 |
|  | 1616386_s_at | 2.71 | Glyoxysomal fatty acid beta-oxidation multifunctional protein MFP-a |
|  | 1614047_s_at | 2.34 | Raffinose synthase family protein |
|  | 1614871_s_at | 0.5 | LisH dimerisation motif;WD40/YVTN repeat-like-containing domain |
|  | 1618573_at | 0.42 | CLAVATA3/ESR-related protein |
|  | 1611800_at | 0.22 | Rhodanese/Cell cycle control phosphatase superfamily protein |
| Biogenesis of cellular component | 1616308_at | 5.33 | Extensin |
|  | 1608585_x_at | 3.44 | Putative proline-rich cell wall protein |
|  | 1615469_at | 3.3 | Hydroxyproline-rich glycoprotein |
|  | 1613973_at | 2.55 | Internal transcribed spacer 2 and 26S ribosomal RNA gene |
|  | 1614832_at | 2.42 | Putative heat-shock protein |
|  | 1620382_at | 2.19 | Putative kinesin light chain |
|  | 1618515_s_at | 2.04 | Methionine sulfoxide reductase (MSS4-like) family protein |
|  | 1613062_at | 0.48 | Pherophorin-C2 protein precursor |
|  | 1617315_at | 0.47 | Kinesin-like calmodulin-binding protein (ZWICHEL) |
|  | 1619401_at | 0.44 | Arabinogalactan protein 16 |
|  | 1616923_s_at | 0.43 | Flagelliform silk protein |
|  | 1622466_at | 0.43 | Tubulin beta 8 |
|  | 1612320_a_at | 0.37 | Tubulin/FtsZ family protein |
|  | 1608124_at | 0.34 | FASCICLIN-like arabinogalactan protein 16 precursor |
|  | 1610991_at | 0.29 | Tubulin alpha-6 chain, putative |
|  | 1611973_at | 0.26 | Extensin |
